# Hypertension and the Risk of All-Cause and Cause-Specific Mortality: An Outcome-Wide Association Study of 67 Causes of Death in the National Health Interview Survey

**DOI:** 10.1155/2021/9376134

**Published:** 2021-07-12

**Authors:** Dagfinn Aune, Wentao Huang, Jing Nie, Yafeng Wang

**Affiliations:** ^1^Department of Epidemiology and Biostatistics, School of Public Health, Imperial College London, London, UK; ^2^Department of Nutrition, Bjørknes University College, Oslo, Norway; ^3^Department of Endocrinology, Morbid Obesity and Preventive Medicine, Oslo University Hospital, Oslo, Norway; ^4^Unit of Cardiovascular and Nutritional Epidemiology, Institute of Environmental Medicine, Karolinska Institutet, Stockholm, Sweden; ^5^School of Nursing, Guangdong Pharmaceutical University, Guangzhou, China; ^6^Department of Sociology & Institute for Empirical Social Science Research, School of Humanities and Social Sciences, Xi'an Jiaotong University, Xi'an, China; ^7^Department of Epidemiology and Biostatistics, School of Health Sciences, Wuhan University, Wuhan, China

## Abstract

**Background:**

Few studies have assessed the association between hypertension and risk of detailed causes of death. We investigated the association between hypertension and all-cause mortality and 67 causes of death in a large cohort.

**Methods:**

Multivariable Cox regression models were used to estimate hazard ratios (HRs) and 95% confidence intervals (95% CIs) for self-reported hypertension vs. no hypertension and mortality. Adults aged ≥18 years (*n* = 213798) were recruited in 1997-2004 and followed through December 31, 2006.

**Results:**

During 5.81 years of follow-up, 11254 deaths occurred. Self-reported hypertension vs. no hypertension was associated with increased risk of all-cause mortality (HR = 1.25, 95% CI: 1.19-1.31) and mortality from septicemia (HR =1.66, 1.06-2.59), other infectious parasitic diseases (HR = 2.67, 1.09-6.51), diabetes mellitus (HR = 1.97, 1.45-2.67), circulatory disease (HR = 1.49, 1.37-1.61), hypertensive heart disease (HR = 3.23, 2.00-5.20), ischemic heart disease (HR = 1.35, 1.23-1.49), acute myocardial infarction (HR = 1.50, 1.27-1.77), other chronic ischemic heart diseases (HR = 1.35, 1.17-1.56), all other forms of heart disease (HR = 1.51, 1.21-1.89), primary hypertension and renal disease (HR = 3.11, 1.82-5.30), cerebrovascular disease (HR = 1.64, 1.37-1.97), other circulatory system diseases (HR = 1.71, 1.09-2.69), other chronic lower respiratory diseases (HR = 1.39, 1.12-1.73), other chronic liver disease (HR = 1.89, 1.06-3.37), renal failure (HR = 1.91, 1.33-2.74), motor vehicle accidents (HR = 1.60, 1.07-2.37), and all other diseases (HR =1.30, 1.10-1.54), but with lower risk of uterine cancer (HR = 0.37, 95% CI: 0.15-0.90) and Alzheimer's disease (HR = 0.65, 95% CI: 0.47-0.92).

**Conclusion:**

Hypertension was associated with increased risk of all-cause mortality and 17 out of 67 causes of death, with most of these being circulatory disease outcomes, however, some of the remaining associations are unlikely to be causal. Further studies are needed to clarify associations with less common causes of death and potential causality across outcomes.

## 1. Introduction

Elevated blood pressure is a major risk factor for several cardiovascular disease outcomes [1, 2] and is the leading cause of death globally with an estimated 10.4 million deaths attributable to elevated systolic blood pressure in 2017 [3]. A positive association has been observed between elevated blood pressure or hypertension and a range of cardiovascular outcomes including ischemic heart disease [2], stable and unstable angina [1], myocardial infarction [1], sudden cardiac death [1, 4], ischemic stroke (1; 2), hemorrhagic stroke [1, 2], hypertensive heart disease [2], peripheral arterial disease [1], heart failure [1, 2], atrial fibrillation [5], abdominal aortic aneurysm [1, 2, 6], aortic dissection [7], and pulmonary embolism [2], as well as kidney disease [8, 9].

Whether hypertension is related to other diseases is less clear and has been less studied. Some studies have suggested associations between hypertension and certain cancers including endometrial [10] and kidney cancer [11–16], while a pooled analysis of Norwegian, Swedish, and Austrian cohort studies with 577000 participants suggested positive associations between elevated blood pressure and a range of cancers including cancers of the oral cavity and pharynx, esophagus, colon, liver, lung and larynx, corpus uteri, kidney, and melanoma as well as overall cancer [17]. Similar results were recently observed among 307000 participants in the EPIC study where an increased risk was observed for cancers of the mouth and pharynx, larynx, esophagus, lung, kidney, breast, and corpus uteri with elevated systolic blood pressure and/or diastolic blood pressure [18]. Some studies have also suggested hypertension may increase the risk of kidney disease [19].

Most previous studies on hypertension and chronic disease risk have focused on specific diseases or groups of diseases (such as cardiovascular diseases, cancer, or kidney disease), and we are not aware of any previous studies on hypertension and risk of very detailed causes of death across different disease groups. For this reason, we examined the association between self-reported hypertension and risk of mortality from all causes and 67 specific causes of death in the National Health Interview Study, to provide a more complete assessment of the potential adverse effects of hypertension across different causes of death.

## 2. Methods

### 2.1. Study Population

The National Health Interview Survey (NHIS) is a national cross-sectional survey, conducted annually by the National Center for Health Statistics in collaboration with the US Census Bureau since 1957. The study used a multistage sample design to monitor the health of the US civilian noninstitutionalized population. A total of 242952 men and women aged ≥18 years participating in the 8 waves conducted during 1997 to 2004 (linked to mortality data through December 2006) were included in the study. We excluded 360 respondents with missing data on hypertension and 28794 participants with history of coronary heart disease, stroke, or cancer at baseline, leaving 213798 participants for inclusion in the final analysis sample (Supplementary Figure 1). All data were based on self-reports and obtained via household roster section of the questionnaire completed by the participants. The design of the NHIS has been reviewed and approved by the Institutional Review Board at the Centers for Disease Control and Prevention. Written informed consent was obtained from all subjects. The current study was based on secondary analyses of publicly available and deidentified data [20].

### 2.2. Mortality

The outcomes included all-cause mortality and cause-specific deaths and were classified using the 10th revision of the International Statistical Classification of Diseases, Injuries, and Causes of Death (ICD-10). Deaths were identified by linkages to the National Death Index through 2006 [21]. Definitions of each outcome examined according to ICD-10 codes are found in Supplementary Table 1.

### 2.3. Assessment of Hypertension

Self-reported hypertension was assessed at the baseline questionnaire which included a question “Have you ever been told by a doctor or other health professional that you had hypertension, also called high blood pressure?”

### 2.4. Covariates

Covariates were selected a priori based on previous literature [22–28] and availability in the dataset. Baseline characteristics associated with hypertension and mortality were included as covariates from the survey and included demographic variables included age, sex, race (Hispanic, non-Hispanic White, non-Hispanic Black, and Other), education level (less than high school degree, high school degree, more than high school degree) and income level (low, middle, high), and lifestyle factors including BMI (<25, 25-<30, ≥30 kg/m^2^), leisure-time physical activity (inactive, insufficient, sufficient), smoking status (never, former, and current cigarette smokers), and alcohol intake (lifetime abstainer, former drinker, current drinker).

### 2.5. Statistical Analysis

Analyses accounted for the complex survey design employed in NHIS by utilizing sample weights, primary sampling, and clustering units via the Taylor series (linearization) method. Person-years of follow-up were calculated for each participant from the recruitment date to the date of death or end of the study period (31 December 2006). Hazard ratios (HRs) and 95% confidence intervals (CIs) for the association between hypertension and all-cause and cause-specific mortality were calculated using multivariable Cox proportional hazards regression model with adjustment for age, sex, race, education, income, BMI, leisure-time physical activity, alcohol, smoking status, and survey year. Missing data were handled by creating missing variable categories. The number of participants with missing data was low (1-4%) (Table 1). Analyses stratified by age, sex, ethnicity/race, alcohol, smoking status, and BMI were conducted to better be able to rule out residual confounding by these risk factors. Further sensitivity analyses were conducted by excluding the first 2 years of follow-up. The analyses were conducted using Stata 13.0 statistical software and *R*-version 3.3.3. All *p* values refer to two-tailed tests, and statistical significance was set at *p* < 0.05.

## 3. Results

From a total sample size of 242952 participants, we excluded 360 participants with missing data on hypertension and 28794 participants with prevalent coronary heart disease, stroke, or cancer at baseline. This left 213798 participants (92931 men and 120089 women) aged 18-85 years for inclusion in the current analysis (Table 1). Participants with self-reported hypertension had lower education, lower income, higher BMI, lower levels of physical activity, a higher prevalence of smokers, and lower prevalence of current drinkers when compared to participants without hypertension (Table 1).

During 1419143 person-years of follow-up (a mean [median] follow-up of 5.81 [5.88] years), 11254 deaths occurred. The most common causes of death were circulatory disease (*n* = 3821), cancer (*n* = 2361), ischemic heart disease (*n* = 2050), other chronic ischemic heart diseases (*n* = 957), acute myocardial infarction (*n* = 777), lung cancer (*n* = 733) and cerebrovascular disease (*n* = 618) (Table 2).

**Figure 1:**
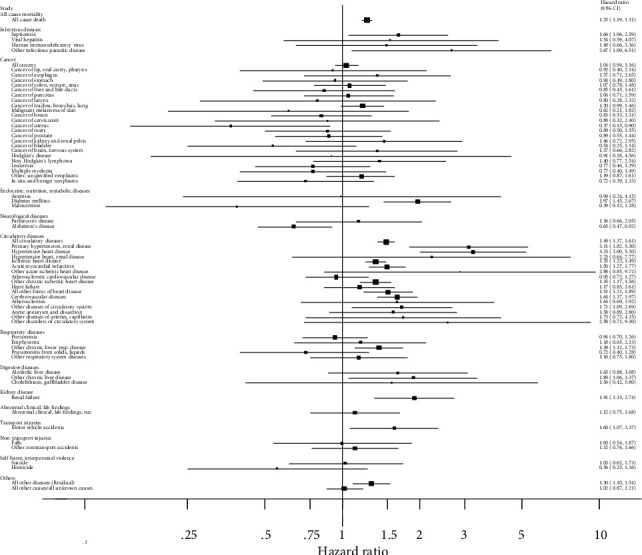
Hazard ratios and 95% confidence intervals for different causes of mortality in participants with hypertension vs. no hypertension.

Participants with hypertension vs. no hypertension were at increased risk of all-cause mortality (HR = 1.25, 95% CI: 1.19-1.31) and mortality from septicemia (HR = 1.66, 1.06-2.59), other infectious parasitic diseases (HR = 2.67, 1.09-6.51), diabetes mellitus (HR = 1.97, 1.45-2.67), circulatory disease (HR = 1.49, 95% CI: 1.37-1.61), hypertensive heart disease (HR = 3.23, 95% CI: 2.00-5.20), ischemic heart disease (HR = 1.35, 1.23-1.49), acute myocardial infarction (HR = 1.50, 1.27-1.77), other chronic ischemic heart diseases (HR = 1.35, 1.17-1.56), all other forms of heart disease (HR = 1.51, 1.21-1.89), primary hypertension, renal disease (HR = 3.11, 1.82-5.30), cerebrovascular disease (HR = 1.64, 1.37-1.97), other diseases of the circulatory system (HR = 1.71, 1.09-2.69), other chronic lower respiratory diseases (HR = 1.39, 1.12-1.73), other chronic liver diseases (HR = 1.89, 1.06-3.37), renal failure (HR = 1.91, 1.33-2.74), motor vehicle accidents (HR = 1.60, 1.07-2.37), and all other diseases (HR = 1.30, 1.10-1.54), while inverse associations were observed for mortality from uterine cancer (HR = 0.37, 95% CI: 0.15-0.90) and Alzheimer's disease (HR = 0.65, 95% CI: 0.47-0.92) (Figure 1, Table 2). No significant association was observed between hypertension and risk of any other outcome; although, nonsignificant positive associations were observed for mortality from lung cancer (HR = 1.20, 95% CI: 0.99-1.46), other ischemic heart diseases (HR = 2.88, 95% CI: 0.85-9.72), aortic aneurysm and dissection (HR = 1.58, 95% CI: 0.89-2.80), and alcoholic liver disease (HR = 1.65, 95% CI: 0.88-3.08) (Figure 1, Table 2).

In sensitivity analyses excluding first 2 years of follow-up, the association between hypertension and all-cause mortality was slightly strengthened (HR = 1.28, 95% CI: 1.21-1.35), and the associations with heart failure 1.51 (95% CI: 1.05-2.16), aortic aneurysm and dissection (HR = 2.47, 95% CI: 1.18-5.15), and kidney failure (HR = 2.23, 95% CI: 1.51-3.28) became stronger, while a few associations lost significance (septicemia, other infectious parasitic diseases, other chronic liver diseases, motor vehicle accidents, Alzheimer's disease) (Supplementary Table 2).

When results were stratified by age, the associations with all-cause mortality and mortality from circulatory disease, hypertensive heart disease, ischemic heart disease, acute myocardial infarction, other chronic ischemic heart diseases, heart failure, all other forms of heart disease, other diseases of the circulatory system, other diseases of arteries or capillaries, other diseases of the circulatory system, kidney failure, and all other diseases were stronger in the younger subjects (<65 years) than in the older participants (≥65 years) (Supplementary Table 3).

There were few differences of the association between hypertension and mortality when analyses were stratified by sex (Supplementary Table 4). The association between hypertension and all-cause mortality was slightly stronger in whites than in other ethnicities (Hispanics, non-Hispanic black, non-Hispanic others combined) (Supplementary Table 5).

The association between hypertension and all-cause mortality was stronger in more highly educated participants compared to participants with low or medium education (Supplementary Table 6). This was also observed for mortality from ischemic heart disease and for other chronic ischemic heart diseases, but for all other forms of heart disease, cerebrovascular disease, and other chronic liver diseases, the opposite was observed (Supplementary Table 6). The association between hypertension and all-cause mortality was stronger in participants with high income compared to participants with a low or medium income, but there were few differences for other outcomes (Supplementary Table 7). The association between hypertension and mortality from diabetes, renal failure, and motor vehicle accidents was restricted to overweight and/or obese participants (Supplementary Table 8). There were little differences in the association between hypertension and mortality when analyses were stratified by physical activity (Supplementary Table 9) and smoking status (Supplementary Table 10).

## 4. Discussion

In this analysis of 213798 participants from the National Health Interview Survey, we found statistically significant positive associations between hypertension and all-cause mortality and mortality from 17 out of 67 causes of death examined. Half of these causes of death were circulatory diseases, and in addition, increased risks were observed for mortality from certain infectious diseases, liver disease, respiratory disease, diabetes, kidney failure, motor vehicle accidents, and a group of all other diseases combined. Overall, the associations with all-cause mortality were stronger in younger than older participants, but similar in men and women, and slightly weaker in whites than in all other ethnicities combined. Associations were also stronger in participants with higher education and income, while few differences were observed when analyses were stratified by BMI, physical activity, and smoking status.

The current findings support some previous observations that elevated blood pressure is associated with increased risk of a large range of cardiovascular disease outcomes (1; 2; 4; 6) and all-cause mortality [29], but also raises the possibility that there may be associations with additional causes of death. The positive association between hypertension and all-cause mortality is probably to a large degree driven by the increased risk of multiple circulatory diseases, but some other causes of death may also have contributed to an elevated overall mortality.

Our findings of a 66% increased risk of mortality from septicemia among participants with hypertension are consistent with results from the REGARD study which found a 49% increased risk [30]. We also found an increased risk of mortality from other infectious parasitic diseases among hypertensive participants, but are not aware of previous studies on this outcome.

Our finding of no association between hypertension and cancer mortality is consistent with a Japanese cohort [29], but in contrast to some recent cohort studies which found positive associations between blood pressure and hypertension and incidence of cancer overall and of specific cancers (17; 18). However, we may also have had too low power to detect moderate or weak associations as most of the confidence intervals were wide. The HR for total cancer mortality was 1.04 (95% CI: 0.94-1.16) comparing hypertensives with non-hypertensives which is comparable to the findings from the EPIC study which reported a more precise HR of 1.03 (95% CI: 1.01-1.05) for total cancer incidence [18].

Our finding that hypertension is associated with increased mortality from diabetes is consistent with several other cohort studies [31–33], but not with a recent MR analysis which found no evidence for a causal association between hypertension and diabetes incidence [34]. Since hypertension is strongly associated with BMI [35] and since the association between hypertension and diabetes mortality was restricted to overweight and obese participants in the NHIS (HRs were 1.29, 2.31, and 2.85 for normal weight, overweight, and obese participants, respectively), the observed association between hypertension and diabetes mortality could be confounded by BMI.

In the current analysis, we found increased risk of all circulatory diseases combined, hypertensive heart disease, ischemic heart disease, acute myocardial infarction, other chronic ischemic heart diseases, all other forms of heart disease, primary hypertension/renal disease, cerebrovascular disease, and other diseases of the circulatory system and additionally for heart failure and aortic aneurysm and dissection after exclusion of the first 2 years of follow-up. For several other circulatory disease outcomes, associations were in the direction of increased risk, but did not reach statistical significance, possibly due to low numbers of deaths. These findings are consistent with previous studies which have consistently shown that hypertension increases the risk of a large number of circulatory diseases [1, 2, 4–7].

There was a positive association between hypertension and chronic lower respiratory disease mortality in the current analysis however, we are not aware of any previous studies which have investigated this association and given the lack of association with mortality from other respiratory diseases and the lack of plausible mechanisms; it is possible that this could have been a chance finding.

Although several studies have found that liver disease may increase blood pressure [36–38], other studies found that hypertension or elevated blood pressure was associated with increased risk of liver disease [39–41], and the latter results are consistent with our finding of an association between hypertension and increased risk of liver disease mortality [39]. Our finding of a positive association between hypertension and kidney disease mortality is consistent with several previous studies [8, 9]. The positive association between hypertension and mortality from motor vehicle accidents was restricted to obese participants, which might suggest confounding by obesity explains this association, as obesity has been associated with increased risk of fatal motor vehicle crashes [42]. The latter association was also attenuated and no longer significant after exclusion of the first two years of follow-up. The positive association with all other diseases could be due to additional diseases being linked to hypertension, misclassification of underlying cause of death, or simply be a chance finding.

A number of biological mechanisms could explain the positive association observed between hypertension and increased circulatory disease mortality. Hypertension induces endothelial dysfunction, exacerbates the atherosclerotic process, and makes the atherosclerotic plaque more unstable [43]. Hypertension also increases risk of left ventricular hypertrophy, which is a risk factor for coronary heart disease [44], heart failure [45], atrial fibrillation [46], and stroke [47]. Hypertension increases the risk of stroke by altering the endothelium and smooth muscle function in the intracerebral arteries, and the endothelial damage and altered blood cell endothelium interaction can lead to local thrombi formation and ischemic lesions.

Hypertension accelerates the atherosclerotic process, increasing the likelihood for cerebral lesions related to stenosis and embolism originating from large extracranial vessels, the aortic arch, and from the heart [48]. High blood pressure contributes to kidney failure by causing damage to the blood vessels that deliver blood to the kidneys, causing the arteries to narrow, weaken, or harden and ultimately failing in delivering sufficient blood to the kidneys [49].

The main limitation of our study is that we relied on self-reported data at baseline regarding hypertension status rather than measured blood pressure, and we were therefore not able to assess these associations across the full range of blood pressure in the population or take into account changes in hypertension status during follow-up. This is likely to have led to misclassification of the exposure, but given the prospective design of the study, such misclassification is most likely to have been non-differential and most likely would have led to attenuation toward the null or underestimation of the observed associations. Although we are not aware of a validation study of the self-reported hypertension data in the NHIS, a recent publication found a similar prevalence of hypertension in the NHIS as in the National Health and Nutrition Examination Survey, which had clinical measurements of blood pressure [50]. Although we adjusted for a range of important confounding factors including age, sex, education, income, alcohol, smoking, BMI, physical activity, and survey year, we cannot exclude the possibility of residual confounding or that additional confounders (e.g., dietary factors) could have influenced the results. We found that many of the observed associations persisted across categories of smoking, BMI, and physical activity, which might suggest that several of the observed associations are likely independent of these risk factors. However, the associations between hypertension and mortality from diabetes, renal failure, and motor vehicle accidents were only observed in the overweight and/or obese participants, and given previously documented associations between adiposity and these outcomes [42, 51, 52], it is possible that confounding by adiposity could explain these results. The duration of follow-up was relatively short, and the number of deaths was modest; so, we may not have had sufficient power to detect significant associations across all causes of death. In addition, given the multiple causes of death investigated, some of the observed associations could have been due to chance. However, the findings are still important as an indicator of outcomes that may need further study in other cohorts.

Strengths of this study include the prospective design and large nationally representative sample which allowed for analyses of detailed causes of death, many of which have been minimally studied previously. We adjusted for important confounding factors, and the results persisted in a number of stratified analyses. Many of the observed results, particularly for cardiovascular outcomes, are consistent with previous studies, lending some credibility to the observed associations.

In conclusion, we found that hypertension was associated with increased risk of all-cause mortality as well as mortality from 17 out of 67 specific causes of death that were examined. Approximately half of these causes were circulatory diseases and the remaining largely metabolic, renal and infectious diseases. Further studies with measured blood pressure are needed to confirm these findings and to assess causality across causes of death, but these results reinforce the important impact of elevated blood pressure across several causes of death.

## Figures and Tables

**Table 1:** Baseline characteristics of participants with hypertension vs. participants without hypertension.

		No hypertension		Hypertension	
		*N*	Percentage	*N*	Percentage
Total		167105	80	46693	20
Age		40.89		56.07	
Sex					
	Men	74619	49	18,627	45
	Women	92486	51	28,066	55
Race/ethnicity					
	Hispanics	31687	12	6,005	8
	Non-Hispanic white	107944	72	30,377	73
	Non-Hispanic black	20983	11	9,138	16
	Non-Hispanic other	6491	5	1,173	3
Education					
	Less than high school	30042	16	12,088	22
	High school degree	46781	29	14,645	33
	More than high school	89317	55	19,634	45
	Missing	965	1	326	1
Income					
	Low	25921	12	7,919	12
	Middle	83726	49	24,882	52
	High	57458	40	13,892	36
BMI					
	<25	76423	46	11,926	25
	25- <30	55691	34	16,421	35
	≥30	29397	17	16,647	36
	Missing	5594	3	1,699	4
Physical activity					
	Inactive	61276	34	21,802	44
	Insufficient activity	31286	19	9,298	20
	Sufficient activity	69210	43	14,222	33
	Missing	5333	3	1,371	3
Smoking status					
	Never	94949	57	24,351	51
	Former	29891	18	12,691	28
	Current	41284	24	9,426	20
	Missing	981	1	225	0
Alcohol intake					
	Never	37249	22	12,336	24
	Former	20156	12	9,351	19
	Current	107249	65	24,462	55
	Missing	2451	1	544	1

**Table 2:** Hazard ratios and 95% confidence intervals (CIs) of all-cause mortality and cause-specific mortality among participants with hypertension compared to participants without hypertension.

	Total	No hypertension		Hypertension		
	*N* (deaths)	*N* (deaths)	HR	*N* (deaths)	HR (95% CI)	*p* value
All-cause mortality	11254	6119	1.00	5135	1.25 (1.19-1.31)	<0.0001
Infections						
Septicemia	140	66	1.00	74	1.66 (1.06-2.59)	0.03
Viral hepatitis	28	18	1.00	10	1.54 (0.59-4.07)	0.38
Human immunodeficiency virus	72	47	1.00	25	1.49 (0.66-3.36)	0.33
Other infectious parasitic disease	31	13	1.00	18	2.67 (1.09-6.51)	0.03
Cancers						
All cancers	2310	1347	1.00	963	1.05 (0.95-1.17)	0.36
Oral cavity, pharynx, lip	27	17	1.00	10	0.92 (0.40-2.14)	0.85
Esophagus	55	31	1.00	24	1.37 (0.71-2.65)	0.35
Stomach	53	32	1.00	21	0.94 (0.49-1.80)	0.85
Colon, rectum, anus	215	125	1.00	90	1.07 (0.78-1.48)	0.67
Liver and bile ducts	68	42	1.00	26	0.85 (0.45-1.61)	0.62
Pancreas	150	81	1.00	69	1.06 (0.71-1.59)	0.78
Larynx	20	13	1.00	7	0.80 (0.28-2.32)	0.68
Lung, trachea, bronchus	733	424	1.00	309	1.20 (0.99-1.46)	0.06
Malignant melanoma	25	17	1.00	8	0.62 (0.21-1.82)	0.38
Breast (females)	128	79	1.00	49	0.83 (0.53-1.31)	0.43
Cervix uteri (females)	19	11	1.00	8	0.88 (0.32-2.40)	0.80
Uterus (females)	32	23	1.00	9	0.37 (0.15-0.90)	0.03
Ovaries (females)	62	36	1.00	26	0.88 (0.50-1.55)	0.66
Prostate (males)	86	49	1.00	37	0.89 (0.55-1.44)	0.63
Kidney and renal pelvis	58	32	1.00	26	1.46 (0.72-2.95)	0.30
Bladder	48	35	1.00	13	0.54 (0.25-1.14)	0.11
Brain, nervous system	42	23	1.00	19	1.37 (0.66-2.82)	0.40
Hodgkin's disease	8	6	1.00	2	0.91 (0.18-4.56)	0.90
Non-Hodgkin's lymphoma	67	34	1.00	33	1.40 (0.77-2.54)	0.27
Leukemia	88	57	1.00	31	0.77 (0.46-1.29)	0.31
Multiple myeloma	37	20	1.00	17	0.77 (0.40-1.49)	0.45
All other and unspecified neoplasms	289	160	1.00	129	1.19 (0.87-1.61)	0.27
In situ and benign neoplasms	51	30	1.00	21	0.72 (0.39-1.33)	0.29
Endocrine, nutritional, metabolic diseases						
Anaemia	17	10	1.00	7	0.99 (0.24-4.15)	0.99
Diabetes mellitus	319	113	1.00	206	1.97 (1.45-2.67)	<0.0001
Malnutrition	14	9	1.00	5	0.39 (0.12-1.28)	0.12
Nervous system						
Parkinson's disease	61	34	1.00	27	1.16 (0.66-2.05)	0.61
Alzheimer's disease	207	128	1.00	79	0.65 (0.47-0.92)	0.01
Circulatory disease						
All circulatory diseases	3821	1756	1.00	2065	1.49 (1.37-1.61)	<0.0001
Primary hypertension, renal disease	115	36	1.00	79	3.11 (1.82-5.30)	<0.0001
Hypertensive heart disease	134	46	1.00	88	3.23 (2.00-5.20)	<0.0001
Hypertensive heart and renal disease	17	4	1.00	13	2.23 (0.64-7.77)	0.21
Ischemic heart disease	2050	991	1.00	1059	1.35 (1.23-1.49)	<0.0001
Acute myocardial infarction	777	359	1.00	418	1.50 (1.27-1.77)	<0.0001
Other acute ischemic heart diseases	15	5	1.00	10	2.88 (0.85-9.72)	0.09
Atherosclerotic cardiovascular disease	301	175	1.00	126	0.95 (0.72-1.27)	0.74
Other chronic ischemic heart diseases	957	452	1.00	505	1.35 (1.17-1.56)	<0.0001
Heart failure	235	110	1.00	125	1.17 (0.85-1.61)	0.33
All other forms of heart disease	480	226	1.00	254	1.51 (1.21-1.89)	<0.0001
Cerebrovascular disease	618	266	1.00	352	1.64 (1.37-1.97)	<0.0001
Atherosclerosis	30	14	1.00	16	1.64 (0.69-3.92)	0.26
Other diseases of the circulatory system	142	63	1.00	79	1.71 (1.09-2.69)	0.02
Aortic aneurysm and dissection	74	32	1.00	42	1.58 (0.89-2.80)	0.12
Other diseases of arteries or capillaries	48	22	1.00	26	1.73 (0.72-4.15)	0.22
Other disorders of the circulatory system	20	9	1.00	11	2.58 (0.71-9.30)	0.15
Respiratory diseases						
Pneumonia	239	137	1.00	102	0.94 (0.70-1.26)	0.66
Emphysema	62	36	1.00	26	1.18 (0.65-2.13)	0.59
Other chronic lower respiratory diseases	492	258	1.00	234	1.39 (1.12-1.73)	0.003
Pneumonitis from solids and liquids	74	47	1.00	27	0.72 (0.40-1.28)	0.26
Other respiratory system diseases	141	74	1.00	67	1.16 (0.75-1.80)	0.49
Digestive diseases						
Alcoholic liver disease	80	51	1.00	29	1.65 (0.88-3.08)	0.12
Other chronic liver diseases	87	46	1.00	41	1.89 (1.06-3.37)	0.03
Cholelithiasis, gallbladder disease	14	5	1.00	9	1.56 (0.42-5.80)	0.51
Urinary tract disease						
Kidney failure	186	71	1.00	115	1.91 (1.33-2.74)	0.001
Abnormal clinical, lab findings	116	65	1.00	51	1.12 (0.75-1.68)	0.58
Transport injuries						
Motor vehicle accidents	173	126	1.00	47	1.60 (1.07-2.37)	0.02
Unintentional injuries			1.00			
Falls	81	51	1.00	30	1.00 (0.54-1.87)	0.99
Other nontransport accidents combined	186	130	1.00	56	1.20 (0.82-1.75)	0.36
Self-harm, interpersonal violence			1.00			
Suicide	151	115	1.00	36	1.03 (0.62-1.73)	0.90
Homicide	56	46	1.00	10	0.56 (0.25-1.26)	0.16
All other diseases (residual)	910	464	1.00	446	1.30 (1.10-1.54)	0.002
All other causes/all unknown causes	1135	826	1.00	309	1.00 (0.85-1.18)	0.99

Multivariable adjustment for age, sex, education, race, income, alcohol, smoking status, BMI, physical activity, and survey year.

## Data Availability

This study used publicly available data from the National Health Interview Survey, and the data are available online https://www.cdc.gov/nchs/nhis/index.htm and https://nhis.ipums.org/nhis/.

## Supplementary Materials

Supplementary Materials Supplementary Table 1: list of ICD codes for cause-specific mortality. Supplementary Table 2: hazard ratios of all-cause mortality and cause-specific mortality among participants with hypertension vs. no hypertension excluding first 2 years of follow-up. Supplementary Table 3: hazard ratios of all-cause mortality and cause-specific mortality among participants with hypertension vs. no hypertension stratified by age. Supplementary Table 4: hazard ratios of all-cause mortality and cause-specific mortality among participants with hypertension vs. no hypertension stratified by sex. Supplementary Table 5: hazard ratios of all-cause mortality and cause-specific mortality among participants with hypertension compared to participants without hypertension stratified by race/ethnicity. Supplementary Table 6: hazard ratios of all-cause mortality and cause-specific mortality among participants with hypertension compared to participants without hypertension stratified by education. Supplementary Table 7: hazard ratios of all-cause mortality and cause-specific mortality among participants with hypertension compared to participants without hypertension stratified by income. Supplementary Table 8: hazard ratios of all-cause mortality and cause-specific mortality among participants with hypertension vs. no hypertension stratified by BMI. Supplementary Table 9: hazard ratios of all-cause mortality and cause-specific mortality participants with hypertension vs. no hypertension, stratified by physical activity. Supplementary Table 10: hazard ratios of all-cause mortality and cause-specific mortality among participants with hypertension vs. no hypertension, stratified by smoking status.
