# Supplementary material for: Hypertension and the Risk of All-Cause and Cause-Specific Mortality: An Outcome-Wide Association Study of 67 Causes of Death in the National Health Interview Survey
Source: Biomed Res Int. 2021 Jul 12;2021:9376134. doi: 10.1155/2021/9376134 (PMC8292050; doi:10.1155/2021/9376134)
Supplement: Supplementary Materials — Supplementary Table 1: list of ICD codes for cause-specific mortality. Supplementary Table 2: hazard ratios of all-cause mortality and cause-specific mortality among participants with hypertension vs. no hypertension excluding first 2 years of follow-up. Supplementary Table 3: hazard ratios of all-cause mortality and cause-specific mortality among participants with hypertension vs. no hypertension stratified by age. Supplementary Table 4: hazard ratios of all-cause mortality and cause-specific mortality among participants with hypertension vs. no hypertension stratified by sex. Supplementary Table 5: hazard ratios of all-cause mortality and cause-specific mortality among participants with hypertension compared to participants without hypertension stratified by race/ethnicity. Supplementary Table 6: hazard ratios of all-cause mortality and cause-specific mortality among participants with hypertension compared to participants without hypertension stratified by education. Supplementary Table 7: hazard ratios of all-cause mortality and cause-specific mortality among participants with hypertension compared to participants without hypertension stratified by income. Supplementary Table 8: hazard ratios of all-cause mortality and cause-specific mortality among participants with hypertension vs. no hypertension stratified by BMI. Supplementary Table 9: hazard ratios of all-cause mortality and cause-specific mortality participants with hypertension vs. no hypertension, stratified by physical activity. Supplementary Table 10: hazard ratios of all-cause mortality and cause-specific mortality among participants with hypertension vs. no hypertension, stratified by smoking status. [file 9376134.f1.doc]

Supplementary Table 1. List of ICD codes for cause-specific mortality

| Cause of death (ICD-10 code) |  |
| --- | --- |
| Infections |  |
| Septicemia | A40-A41 |
| Viral hepatitis | B15-B19 |
| Human immunodeficiency virus | B20-B24 |
| Other infectious and parasitic disease | A00,A05,A20-A36,A42- A44,A48-A49,A54-A79,A81-A82,A85.0-A85.1,A85.8, A86-B04,B06-B09,B25-B49,B55-B99 |
| Cancers |  |
| All cancers | C00-C97 |
| Oral cavity, pharynx, lip | C00-C14 |
| Esophagus | C15 |
| Stomach | C16 |
| Colon, rectum, anus | C18-C21 |
| Liver and bile ducts | C22 |
| Pancreas | C25 |
| Larynx | C32 |
| Lung, trachea, bronchus | C33-C34 |
| Malignant melanoma | C43 |
| Breast females | C50 |
| Cervix uteri females | C53 |
| Uterus females | C54-C55 |
| Ovaries females | C56 |
| Prostate males | C61 |
| Kidney and renal pelvis | C64-C65 |
| Bladder | C67 |
| Brain, nervous system | C70-C72 |
| Hodgkin's disease | C81 |
| Non-Hodgkin's lymphoma | C82-C85 |
| Leukemia | C91-C95 |
| Multiple myeloma and immunoproliferative neoplasms | C88-C90 |
| All other and unspecified neoplasms | C17,C23-C24,C26-C31,C37-C41, C44- C49,C51-C52,C57-C60,C62-C63,C66,C68-C69,C73-C80,C97 |
| In situ and benign neoplasms | D00-D48 |
| Endocrine, nutritional, metabolic diseases |  |
| Anaemia | D50-D64 |
| Diabetes mellitus | E10-E14 |
| Malnutrition | E40-E46 |
| Nervous system |  |
| Parkinson's disease | G20-G21 |
| Alzheimer's disease | G30 |
| Circulatory disease |  |
| All circulatory diseases | I10-I13, I20-I25, I26-I28, I34-I38, I42-I49, I50, I51, I60-I69, I70-I78, I80-I99 |
| Primary hypertension and hypertensive renal disease | I10, I12 |
| Hypertensive heart disease | I11 |
| Hypertensive heart and renal disease | I13 |
| Ischaemic heart disease | I20-I25 |
| Acute myocardial infarction | I21-I22 |
| Other acute ischaemic heart disease | I24 |
| Atherosclerotic cardiovascular disease | I25.0 |
| Other chronic ischaemic heart disease | I20, I25.1-I25.9 |
| Heart failure | I50 |
| All other forms of heart disease | I26-I28,I34-I38,I42-I49,I51 |
| Cerebrovascular disease | I60-I69 |
| Atherosclerosis | I70 |
| Other diseases of circulatory system | I71-I78 |
| Aortic aneurysm and dissection | I71 |
| Other diseases of arteries or capillaries | I72-I78 |
| Other disorders of circulatory system | I80-I99 |
| Respiratory diseases |  |
| Pneumonia | J12-J18 |
| Emphysema | J43 |
| Other chronic lower respiratory disease | J44, J47 |
| Pneumonitis from solids, liquids | J69 |
| Other respiratory system diseases | J00-J06, J30-J39, J67, J70-J98 |
| Digestive diseases |  |
| Alcoholic liver disease | K70 |
| Other chronic liver disease | K73-K74 |
| Cholelithiasis, gallbladder disease | K80-K82 |
| Urinary tract disease |  |
| Kidney failure | N17-N19 |
| Abnormal clinical, lab findings | R00-R99 |
| Transport injuries |  |
| Motor vehicle accidents | V02-V04,V09.0,V09.2,V12-V14,V19.0-V19.2, V19.4-V19.6,V20-V79,V80.3-V80.5,V81.0-V81.1,V82.0-V82.1,V83-V86, V87.0-V87.8,V88.0-V88.8,V89.0,V89.2 |
| Unintentional injuries |  |
| Falls | W00-W19 |
| Other non-transport accidents combined | W20-W31,W35- W99, X00-X59,Y86 |
| Self-harm, interpersonal violence |  |
| Suicide | X60-X84, Y87.0 |
| Homicide | X85-Y09, Y87.1 |
| All other diseases (residual ) | D65-E07,E15-E34,E65-F99,G04-G12,G23-G25, G31-H93,K00- K22,K29-K31,K50-K66,K71-K72,K75-K76,K83-M99,N13.0-N13.5, N13.7-N13.9,N14,N15.0,N15.8- N15.9,N20-N23,N28-N39,N41-N64,N80-N98 |
| All other causes/all unknown causes | A01-A02, A03,A06, A04, A07-A09, A16-A19, A37, A38, A46, A39, A50-A53, A80, A83-A84, A85.2, B05, B50-B54, C96, E50-E64, G00, G03, I00-I09, I33, I30-I31, I40, J10-J11, J20-J21, J22, J40-J42, J45-J46, J60-J66, J68, K25-K28, K35-K38, K40-K46, N00-N07, N10-N12, N13.6, N15.1, N25-N27, N40, N70-N76, O00-O99, P00-P96, Q00-Q99, V01, V05-V06, V09.1, V09.3-V09.9, V10-V11, V15-V18, V19.3, V19.8-V19.9, V80.0-V80.2, V80.6-V80.9, V81.2-V81.9, V82.2-V82.9, V87.9, V88.9, V89.1, V89.3, V89.9, V90-V99, W32-W34, Y10-Y21-Y36, Y40-Y85, Y87.2, Y88, Y89.0, Y89.1, Y89.9 and unknown causes. |

Supplementary Table 2. Hazard ratios of all-cause mortality and cause-specific mortality among participants with hypertension vs. no hypertension excluding first 2 years of follow-up

|  | Total | No hypertension | | Hypertension | |  |
| --- | --- | --- | --- | --- | --- | --- |
|  | N  (deaths) | N  (deaths) | HR | N (deaths) | HR (95% CI) | P-value |
| All-cause mortality | 8643 | 4686 | 1.00 | 3957 | 1.28 (1.21-1.35) | <0.0001 |
| Infections |  |  |  |  |  |  |
| Septicemia | 111 | 56 | 1.00 | 55 | 1.31 (0.82-2.09) | 0.26 |
| Viral hepatitis | 19 | 13 | 1.00 | 6 | 1.27 (0.36-4.49) | 0.71 |
| Human immunodeficiency virus | 55 | 34 | 1.00 | 21 | 1.93 (0.74-5.01) | 0.18 |
| Other infectious parasitic disease | 21 | 10 | 1.00 | 11 | 2.25 (0.83-6.06) | 0.11 |
| Cancers |  |  |  |  |  |  |
| All cancers | 1871 | 1100 | 1.00 | 771 | 1.07 (0.96-1.20) | 0.22 |
| Oral cavity, pharynx, lip | 23 | 13 | 1.00 | 10 | 1.26 (0.53-2.99) | 0.60 |
| Esophagus | 45 | 25 | 1.00 | 20 | 1.23 (0.59-2.56) | 0.59 |
| Stomach | 44 | 29 | 1.00 | 15 | 0.67 (0.32-1.41) | 0.29 |
| Colon, rectum, anus | 175 | 105 | 1.00 | 70 | 1.00 (0.70-1.42) | 0.99 |
| Liver and bile ducts | 54 | 34 | 1.00 | 20 | 0.84 (0.41-1.71) | 0.63 |
| Pancreas | 118 | 65 | 1.00 | 53 | 1.08 (0.71-1.66) | 0.71 |
| Larynx | 15 | 11 | 1.00 | 4 | 0.74 (0.19-2.83) | 0.66 |
| Lung, trachea, bronchus | 594 | 350 | 1.00 | 244 | 1.22 (0.99-1.51) | 0.07 |
| Malignant melanoma | 23 | 15 | 1.00 | 8 | 0.74 (0.24-2.29) | 0.60 |
| Breast (females) | 106 | 65 | 1.00 | 41 | 0.81 (0.49-1.34) | 0.42 |
| Cervix uteri (females) | 18 | 10 | 1.00 | 8 | 0.98 (0.35-2.71) | 0.97 |
| Uterus (females) | 27 | 20 | 1.00 | 7 | 0.33 (0.12-0.92) | 0.03 |
| Ovaries (females) | 47 | 28 | 1.00 | 19 | 0.84 (0.44-1.60) | 0.59 |
| Prostate (males) | 69 | 37 | 1.00 | 32 | 1.12 (0.67-1.89) | 0.66 |
| Kidney and renal pelvis | 51 | 28 | 1.00 | 23 | 1.64 (0.77-3.49) | 0.20 |
| Bladder | 43 | 30 | 1.00 | 13 | 0.59 (0.27-1.28) | 0.18 |
| Brain, nervous system | 32 | 19 | 1.00 | 13 | 1.07 (0.49-2.31) | 0.87 |
| Hodgkin's disease | 6 | 5 | 1.00 | 1 | - | - |
| Non-Hodgkin's lymphoma | 51 | 26 | 1.00 | 25 | 1.42 (0.74-2.75) | 0.30 |
| Leukemia | 63 | 39 | 1.00 | 24 | 0.92 (0.51-1.66) | 0.78 |
| Multiple myeloma | 33 | 18 | 1.00 | 15 | 0.75 (0.37-1.49) | 0.40 |
| All other and unspecified neoplasms | 234 | 128 | 1.00 | 106 | 1.34 (0.95-1.87) | 0.09 |
| In situ and benign neoplasms | 38 | 23 | 1.00 | 15 | 0.81 (0.40-1.62) | 0.55 |
| Endocrine, nutritional, metabolic diseases |  |  |  |  |  |  |
| Anaemia | 15 | 10 | 1.00 | 5 | 0.42 (0.09-1.94) | 0.27 |
| Diabetes mellitus | 251 | 98 | 1.00 | 153 | 1.74 (1.23-2.45) | 0.002 |
| Malnutrition | 11 | 7 | 1.00 | 4 | - | - |
| Nervous system |  |  |  |  |  |  |
| Parkinson's disease | 51 | 28 | 1.00 | 23 | 1.26 (0.67-2.35) | 0.47 |
| Alzheimer's disease | 179 | 106 | 1.00 | 73 | 0.70 (0.49-1.00) | 0.05 |
| Circulatory disease |  |  |  |  |  |  |
| All circulatory diseases | 2857 | 1312 | 1.00 | 1545 | 1.54 (1.41-1.69) | <0.0001 |
| Hypertensive heart disease | 99 | 37 | 1.00 | 62 | 2.70 (1.57-4.63) | <0.0001 |
| Hypertensive heart and renal disease | 9 | 2 | 1.00 | 7 | 2.77 (0.59-13.08) | 0.20 |
| Ischaemic heart disease | 1517 | 730 | 1.00 | 787 | 1.40 (1.24-1.57) | <0.0001 |
| Acute myocardial infarction | 546 | 244 | 1.00 | 302 | 1.60 (1.29-1.98) | <0.0001 |
| Other acute ischaemic heart disease | 12 | 5 | 1.00 | 7 | 2.44 (0.60-9.93) | 0.21 |
| Atherosclerotic cardiovascular disease | 220 | 131 | 1.00 | 89 | 0.96 (0.70-1.32) | 0.79 |
| Other chronic ischaemic heart disease | 739 | 350 | 1.00 | 389 | 1.38 (1.17-1.64) | <0.0001 |
| Heart failure | 189 | 79 | 1.00 | 110 | 1.51 (1.05-2.16) | 0.03 |
| All other forms of heart disease | 359 | 177 | 1.00 | 182 | 1.46 (1.12-1.91) | 0.006 |
| Primary hypertension, renal disease | 96 | 31 | 1.00 | 65 | 3.06 (1.71-5.47) | <0.0001 |
| Cerebrovascular disease | 470 | 206 | 1.00 | 264 | 1.61 (1.28-2.02) | <0.0001 |
| Atherosclerosis | 25 | 10 | 1.00 | 15 | 2.45 (0.96-6.24) | 0.06 |
| Other diseases of circulatory system | 93 | 40 | 1.00 | 53 | 2.16 (1.20-3.90) | 0.01 |
| Aortic aneurysm and dissection | 42 | 16 | 1.00 | 26 | 2.47 (1.18-5.15) | 0.02 |
| Other diseases of arteries or capillaries | 38 | 16 | 1.00 | 22 | 2.16 (0.78-6.00) | 0.14 |
| Other disorders of circulatory system | 13 | 8 | 1.00 | 5 | 1.32 (0.25-6.92) | 0.74 |
| Respiratory diseases |  |  |  |  |  |  |
| Pneumonia | 198 | 112 | 1.00 | 86 | 0.96 (0.69-1.33) | 0.79 |
| Emphysema | 46 | 25 | 1.00 | 21 | 1.51 (0.78-2.94) | 0.23 |
| Other chronic lower respiratory disease | 380 | 199 | 1.00 | 181 | 1.34 (1.05-1.71) | 0.02 |
| Pneumonitis from solids, liquids | 64 | 39 | 1.00 | 25 | 0.84 (0.45-1.57) | 0.59 |
| Other respiratory system diseases | 114 | 65 | 1.00 | 49 | 1.02 (0.62-1.67) | 0.95 |
| Digestive diseases |  |  |  |  |  |  |
| Alcoholic liver disease | 57 | 36 | 1.00 | 21 | 1.76 (0.83-3.74) | 0.14 |
| Other chronic liver disease | 68 | 38 | 1.00 | 30 | 1.81 (0.95-3.44) | 0.07 |
| Cholelithiasis, gallbladder disease | 14 | 5 | 1.00 | 9 | 1.56 (0.42-5.80) | 0.51 |
| Urinary tract disease |  |  |  |  |  |  |
| Kidney failure | 149 | 54 | 1.00 | 95 | 2.23 (1.51-3.28) | <0.0001 |
| Abnormal clinical, lab findings | 89 | 51 | 1.00 | 38 | 1.14 (0.72-1.79) | 0.58 |
| Transport injuries |  |  |  |  |  |  |
| Motor vehicle accidents | 120 | 85 | 1.00 | 35 | 1.45 (0.91-2.32) | 0.12 |
| Unintentional injuries |  |  | 1.00 |  |  |  |
| Falls | 58 | 33 | 1.00 | 25 | 1.33 (0.74-2.36) | 0.34 |
| Other non-transport accidents combined | 140 | 96 | 1.00 | 44 | 1.24 (0.80-1.90) | 0.33 |
| Self-harm, interpersonal violence |  |  | 1.00 |  |  |  |
| Suicide | 93 | 70 | 1.00 | 23 | 1.36 (0.66-2.80) | 0.40 |
| Homicide | 42 | 35 | 1.00 | 7 | 0.48 (0.18-1.26) | 0.14 |
| All other diseases (residual) | 717 | 355 | 1.00 | 362 | 1.44 (1.19-1.74) | <0.0001 |
| All other causes/all unknown causes | 815 | 591 | 1.00 | 224 | 0.99 (0.82-1.20) | 0.93 |

Multivariable adjustment for age, sex, education, race, income, alcohol, smoking status, BMI, physical activity, and survey year

Supplementary Table 3. Hazard ratios of all-cause mortality and cause-specific mortality among participants with hypertension vs. no hypertension stratified by age

|  |  | Total | No hypertension | | Hypertension | |  |
| --- | --- | --- | --- | --- | --- | --- | --- |
|  | Age | N  (deaths) | N  (deaths) | HR | N (deaths) | HR (95% CI) | P-value |
| All-cause mortality | <65 | 4334 | 2830 | 1.00 | 1504 | 1.48 (1.36-1.61) | <0.0001 |
|  | ≥65 | 6920 | 3289 | 1.00 | 3631 | 1.17 (1.10-1.24) | <0.0001 |
| Infections |  |  |  |  |  |  |  |
| Septicemia | <65 | 54 | 27 | 1.00 | 27 | 2.53 (1.27-5.06) | 0.009 |
|  | ≥65 | 86 | 39 | 1.00 | 47 | 1.23 (0.73-2.05) | 0.43 |
| Viral hepatitis | <65 | 23 | 15 | 1.00 | 8 | 1.67 (0.59-4.74) | 0.33 |
|  | ≥65 | 5 | 3 | 1.00 | 2 | 0.94 (0.07-12.57) | 0.96 |
| Human immunodeficiency virus | <65 | 69 | 47 | 1.00 | 22 | 1.36 (0.59-3.13) | 0.47 |
|  | ≥65 | 3 | 0 | 1.00 | 3 | - | - |
| Other infectious parasitic disease | <65 | 12 | 6 | 1.00 | 6 | 3.70 (1.01-13.49) | 0.048 |
|  | ≥65 | 19 | 7 | 1.00 | 12 | 2.02 (0.75-5.40) | 0.16 |
| Cancers |  |  |  |  |  |  |  |
| All cancers | <65 | 1004 | 668 | 1.00 | 336 | 1.07 (0.91-1.26) | 0.43 |
|  | ≥65 | 1306 | 679 | 1.00 | 627 | 1.02 (0.90-1.16) | 0.77 |
| Oral cavity, pharynx, lip | <65 | 15 | 9 | 1.00 | 6 | 0.88 (0.29-2.72) | 0.83 |
|  | ≥65 | 12 | 8 | 1.00 | 4 | 0.82 (0.25-2.74) | 0.75 |
| Esophagus | <65 | 30 | 19 | 1.00 | 11 | 1.04 (0.40-2.69) | 0.93 |
|  | ≥65 | 25 | 12 | 1.00 | 13 | 1.70 (0.69-4.18) | 0.25 |
| Stomach | <65 | 22 | 14 | 1.00 | 8 | 0.94 (0.30-2.97) | 0.91 |
|  | ≥65 | 31 | 18 | 1.00 | 13 | 0.96 (0.43-2.14) | 0.93 |
| Colon, rectum, anus | <65 | 85 | 58 | 1.00 | 27 | 1.09 (0.59-1.99) | 0.78 |
|  | ≥65 | 130 | 67 | 1.00 | 63 | 1.06 (0.70-1.59) | 0.79 |
| Liver and bile ducts | <65 | 33 | 24 | 1.00 | 9 | 0.91 (0.36-2.32) | 0.84 |
|  | ≥65 | 35 | 18 | 1.00 | 17 | 0.83 (0.39-1.78) | 0.63 |
| Pancreas | <65 | 57 | 40 | 1.00 | 17 | 0.68 (0.35-1.32) | 0.26 |
|  | ≥65 | 93 | 41 | 1.00 | 52 | 1.32 (0.78-2.22) | 0.30 |
| Larynx | <65 | 9 | 5 | 1.00 | 4 | 1.50 (0.34-6.60) | 0.59 |
|  | ≥65 | 11 | 8 | 1.00 | 3 | 0.50 (0.11-2.30) | 0.37 |
| Lung, trachea, bronchus | <65 | 325 | 205 | 1.00 | 120 | 1.30 (0.98-1.72) | 0.07 |
|  | ≥65 | 408 | 219 | 1.00 | 189 | 1.05 (0.84-1.32) | 0.65 |
| Malignant melanoma | <65 | 16 | 12 | 1.00 | 4 | 0.56 (0.12-2.63) | 0.46 |
|  | ≥65 | 9 | 5 | 1.00 | 4 | 0.73 (0.18-2.95) | 0.65 |
| Breast (females) | <65 | 74 | 56 | 1.00 | 18 | 0.68 (0.37-1.25) | 0.21 |
|  | ≥65 | 54 | 23 | 1.00 | 31 | 1.07 (0.52-2.19) | 0.85 |
| Cervix uteri (females) | <65 | 15 | 10 | 1.00 | 5 | 0.73 (0.22-2.42) | 0.61 |
|  | ≥65 | 4 | 1 | 1.00 | 3 | 2.44 (0.28-21.17) | 0.42 |
| Uterus (females) | <65 | 14 | 12 | 1.00 | 2 | 0.18 (0.04-0.83) | 0.03 |
|  | ≥65 | 18 | 11 | 1.00 | 7 | 0.46 (0.16-1.33) | 0.15 |
| Ovaries (females) | <65 | 27 | 20 | 1.00 | 7 | 0.82 (0.34-1.98) | 0.65 |
|  | ≥65 | 35 | 16 | 1.00 | 19 | 0.94 (0.44-2.00) | 0.87 |
| Prostate (males) | <65 | 18 | 11 | 1.00 | 7 | - | - |
|  | ≥65 | 68 | 38 | 1.00 | 30 | 0.80 (0.47-1.37) | 0.22 |
| Kidney and renal pelvis | <65 | 27 | 14 | 1.00 | 13 | 2.44 (0.92-6.46) | 0.07 |
|  | ≥65 | 31 | 18 | 1.00 | 13 | 0.74 (0.31-1.76) | 0.50 |
| Bladder | <65 | 12 | 9 | 1.00 | 3 | 0.40 (0.08-1.92) | 0.25 |
|  | ≥65 | 36 | 26 | 1.00 | 10 | 0.59 (0.26-1.37) | 0.22 |
| Brain, nervous system | <65 | 19 | 12 | 1.00 | 7 | 1.91 (0.63-5.79) | 0.25 |
|  | ≥65 | 23 | 11 | 1.00 | 12 | 0.95 (0.41-2.23) | 0.91 |
| Hodgkin's disease | <65 | 4 | 4 | 1.00 | 0 | - | - |
|  | ≥65 | 4 | 2 | 1.00 | 2 | - | - |
| Non-Hodgkin's lymphoma | <65 | 20 | 14 | 1.00 | 6 | 1.15 (0.34-3.86) | 0.82 |
|  | ≥65 | 47 | 20 | 1.00 | 27 | 1.44 (0.74-2.80) | 0.28 |
| Leukemia | <65 | 32 | 26 | 1.00 | 6 | 0.47 (0.18-1.20) | 0.11 |
|  | ≥65 | 56 | 31 | 1.00 | 25 | 0.93 (0.51-1.70) | 0.81 |
| Multiple myeloma | <65 | 13 | 8 | 1.00 | 5 | - | - |
|  | ≥65 | 24 | 12 | 1.00 | 12 | 0.97 (0.42-2.25) | 0.95 |
| All other and unspecified neoplasms | <65 | 137 | 86 | 1.00 | 51 | 1.29 (0.84-1.99) | 0.24 |
|  | ≥65 | 152 | 74 | 1.00 | 78 | 1.12 (0.75-1.69) | 0.58 |
| In situ and benign neoplasms | <65 | 11 | 7 | 1.00 | 4 | 0.90 (0.20-4.14) | 0.89 |
|  | ≥65 | 40 | 23 | 1.00 | 17 | 0.66 (0.34-1.26) | 0.21 |
| Endocrine, nutritional, metabolic diseases |  |  |  |  |  |  |  |
| Anaemia | <65 | 6 | 5 | 1.00 | 1 | 0.45 (0.03-8.26) | 0.59 |
|  | ≥65 | 11 | 5 | 1.00 | 6 | 2.17 (0.45-10.52) | 0.33 |
| Diabetes mellitus | <65 | 131 | 61 | 1.00 | 70 | 1.83 (1.13-2.97) | 0.02 |
|  | ≥65 | 188 | 52 | 1.00 | 136 | 1.96 (1.35-2.86) | <0.0001 |
| Malnutrition | <65 | 1 | 1 | 1.00 | 0 | - | - |
|  | ≥65 | 13 | 8 | 1.00 | 5 | 0.40 (0.12-1.35) | 0.14 |
| Nervous system |  |  |  |  |  |  |  |
| Parkinson's disease | <65 | 3 | 2 | 1.00 | 1 | - | - |
|  | ≥65 | 58 | 32 | 1.00 | 26 | 1.10 (0.62-1.97) | 0.74 |
| Alzheimer's disease | <65 | 9 | 5 | 1.00 | 4 | 1.52 (0.38-6.09) | 0.56 |
|  | ≥65 | 198 | 123 | 1.00 | 75 | 0.62 (0.44-0.86) | 0.005 |
| Circulatory disease |  |  |  |  |  |  |  |
| All circulatory diseases | <65 | 973 | 487 | 1.00 | 486 | 2.23 (1.89-2.63) | <0.0001 |
|  | ≥65 | 2848 | 1269 | 1.00 | 1579 | 1.31 (1.20-1.43) | <0.0001 |
| Hypertensive heart disease | <65 | 62 | 23 | 1.00 | 39 | 4.85 (2.38-9.86) | <0.0001 |
|  | ≥65 | 72 | 23 | 1.00 | 49 | 2.27 (1.33-3.87) | 0.003 |
| Hypertensive heart and renal disease | <65 | 4 | 1 | 1.00 | 3 | 1.70 (0.27-10.74) | 0.57 |
|  | ≥65 | 13 | 3 | 1.00 | 10 | 2.61 (0.60-11.38) | 0.20 |
| Ischaemic heart disease | <65 | 539 | 284 | 1.00 | 255 | 1.78 (1.45-2.19) | <0.0001 |
|  | ≥65 | 1511 | 707 | 1.00 | 804 | 1.21 (1.08-1.36) | 0.001 |
| Acute myocardial infarction | <65 | 230 | 113 | 1.00 | 117 | 1.79 (1.31-2.46) | <0.0001 |
|  | ≥65 | 547 | 246 | 1.00 | 301 | 1.34 (1.11-1.63) | 0.002 |
| Other acute ischaemic heart disease | <65 | 6 | 3 | 1.00 | 3 | - | - |
|  | ≥65 | 9 | 2 | 1.00 | 7 | 2.24 (0.54-9.19) | 0.26 |
| Atherosclerotic cardiovascular disease | <65 | 102 | 68 | 1.00 | 34 | 1.05 (0.64-1.72) | 0.84 |
|  | ≥65 | 199 | 107 | 1.00 | 92 | 0.91 (0.64-1.30) | 0.61 |
| Other chronic ischaemic heart disease | <65 | 201 | 100 | 1.00 | 101 | 2.20 (1.53-3.17) | <0.0001 |
|  | ≥65 | 756 | 352 | 1.00 | 404 | 1.20 (1.02-1.41) | 0.03 |
| Heart failure | <65 | 27 | 12 | 1.00 | 15 | 2.70 (0.96-7.63) | 0.06 |
|  | ≥65 | 208 | 98 | 1.00 | 110 | 1.07 (0.77-1.47) | 0.70 |
| All other forms of heart disease | <65 | 140 | 70 | 1.00 | 70 | 2.88 (1.93-4.32) | <0.0001 |
|  | ≥65 | 340 | 156 | 1.00 | 184 | 1.21 (0.95-1.55) | 0.12 |
| Primary hypertension, renal disease | <65 | 27 | 10 | 1.00 | 17 | 5.55 (2.04-15.11) | 0.001 |
|  | ≥65 | 88 | 26 | 1.00 | 62 | 2.61 (1.44-4.71) | 0.002 |
| Cerebrovascular disease | <65 | 128 | 72 | 1.00 | 56 | 1.87 (1.22-2.87) | 0.004 |
|  | ≥65 | 490 | 194 | 1.00 | 296 | 1.62 (1.30-2.02) | <0.0001 |
| Atherosclerosis | <65 | 2 | 1 | 1.00 | 1 | - | - |
|  | ≥65 | 28 | 13 | 1.00 | 15 | 1.71 (0.69-4.21) | 0.25 |
| Other diseases of circulatory system | <65 | 44 | 14 | 1.00 | 30 | 5.07 (2.02-12.70) | 0.001 |
|  | ≥65 | 98 | 49 | 1.00 | 49 | 0.97 (0.62-1.52) | 0.89 |
| Aortic aneurysm and dissection | <65 | 23 | 8 | 1.00 | 15 | 2.60 (0.89-7.59) | 0.08 |
|  | ≥65 | 51 | 24 | 1.00 | 27 | 1.16 (0.61-2.22) | 0.65 |
| Other diseases of arteries or capillaries | <65 | 12 | 4 | 1.00 | 8 | 11.66 (11.96-69.27) | 0.007 |
|  | ≥65 | 36 | 18 | 1.00 | 18 | 0.88 (0.40-1.94) | 0.75 |
| Other disorders of circulatory system | <65 | 9 | 2 | 1.00 | 7 | 12.76 (2.93-55.59) | 0.001 |
|  | ≥65 | 11 | 7 | 1.00 | 4 | 0.54 (0.12-2.34) | 0.41 |
| Respiratory diseases |  |  |  |  |  |  |  |
| Pneumonia | <65 | 49 | 39 | 1.00 | 10 | 0.56 (0.24-1.31) | 0.18 |
|  | ≥65 | 190 | 98 | 1.00 | 92 | 1.07 (0.78-1.49) | 0.66 |
| Emphysema | <65 | 16 | 10 | 1.00 | 6 | - | - |
|  | ≥65 | 46 | 26 | 1.00 | 20 | 0.95 (0.49-1.83) | 0.88 |
| Other chronic lower respiratory disease | <65 | 111 | 62 | 1.00 | 49 | 1.82 (1.14-2.90) | 0.01 |
|  | ≥65 | 381 | 196 | 1.00 | 185 | 1.24 (0.96-1.59) | 0.10 |
| Pneumonitis from solids, liquids | <65 | 11 | 9 | 1.00 | 2 | 0.25 (0.04-1.52) | 0.13 |
|  | ≥65 | 63 | 38 | 1.00 | 25 | 0.81 (0.45-1.47) | 0.49 |
| Other respiratory system diseases | <65 | 44 | 26 | 1.00 | 18 | 1.29 (0.53-3.15) | 0.58 |
|  | ≥65 | 97 | 48 | 1.00 | 49 | 1.12 (0.70-1.77) | 0.64 |
| Digestive diseases |  |  |  |  |  |  |  |
| Alcoholic liver disease | <65 | 67 | 45 | 1.00 | 22 | 1.45 (0.75-2.79) | 0.27 |
|  | ≥65 | 13 | 6 | 1.00 | 7 | 2.42 (0.48-12.19) | 0.28 |
| Other chronic liver disease | <65 | 59 | 34 | 1.00 | 25 | 2.07 (1.09-3.94) | 0.03 |
|  | ≥65 | 28 | 12 | 1.00 | 16 | 1.33 (0.53-3.34) | 0.54 |
| Cholelithiasis, gallbladder disease | <65 | 1 | 0 | 1.00 | 1 | - | - |
|  | ≥65 | 13 | 5 | 1.00 | 8 | 1.45 (0.40-5.27) | 0.57 |
| Urinary tract disease |  |  |  |  |  |  |  |
| Kidney failure | <65 | 44 | 14 | 1.00 | 30 | 4.85 (2.40-9.77) | <0.0001 |
|  | ≥65 | 142 | 57 | 1.00 | 85 | 1.47 (0.98-2.20) | 0.06 |
| Abnormal clinical, lab findings | <65 | 48 | 34 | 1.00 | 14 | 0.88 (0.45-1.74) | 0.72 |
|  | ≥65 | 68 | 31 | 1.00 | 37 | 1.36 (0.78-2.35) | 0.28 |
| Transport injuries |  |  |  |  |  |  |  |
| Motor vehicle accidents | <65 | 133 | 107 | 1.00 | 26 | 1.66 (1.03-2.68) | 0.04 |
|  | ≥65 | 40 | 19 | 1.00 | 21 | 1.45 (0.71-2.96) | 0.31 |
| Unintentional injuries |  |  |  |  |  |  |  |
| Falls | <65 | 28 | 22 | 1.00 | 6 | 1.79 (0.53-6.07) | 0.35 |
|  | ≥65 | 53 | 29 | 1.00 | 24 | 0.79 (0.41-1.53) | 0.48 |
| Other non-transport accidents combined | <65 | 125 | 99 | 1.00 | 26 | 1.10 (0.63-1.91) | 0.81 |
|  | ≥65 | 61 | 31 | 1.00 | 30 | 1.40 (0.78-2.52) | 0.26 |
| Self-harm, interpersonal violence |  |  |  |  |  |  |  |
| Suicide | <65 | 131 | 105 | 1.00 | 26 | 1.02 (0.56-1.86) | 0.94 |
|  | ≥65 | 20 | 10 | 1.00 | 10 | 1.19 (0.49-2.88) | 0.70 |
| Homicide | <65 | 51 | 44 | 1.00 | 7 | 0.47 (0.18-1.28) | 0.14 |
|  | ≥65 | 5 | 2 | 1.00 | 3 | - | - |
| All other diseases (residual) | <65 | 311 | 185 | 1.00 | 126 | 1.85 (1.35-2.54) | <0.0001 |
|  | ≥65 | 599 | 279 | 1.00 | 320 | 1.11 (0.91-1.36) | 0.29 |
| All other causes/all unknown causes | <65 | 809 | 664 | 1.00 | 145 | 1.00 (0.81-1.24) | 0.99 |
|  | ≥65 | 326 | 162 | 1.00 | 164 | 1.05 (0.79-1.38) | 0.75 |

Multivariable adjustment for age, sex, education, race, income, alcohol, smoking status, BMI, physical activity, and survey year

Supplementary Table 4. Hazard ratios of all-cause mortality and cause-specific mortality among participants with hypertension vs. no hypertension stratified by sex

|  |  | Total | No diabetes | | Type 2 diabetes | |  |
| --- | --- | --- | --- | --- | --- | --- | --- |
|  | Sex | N  (deaths) | N  (deaths) | HR | N (deaths) | HR (95% CI) | P-value |
| All-cause mortality | Men | 5225 | 3179 | 1.00 | 2046 | 1.24 (1.16-1.33) | <0.0001 |
|  | Women | 6029 | 2940 | 1.00 | 3089 | 1.24 (1.15-1.33) | <0.0001 |
| Infections |  |  |  |  |  |  |  |
| Septicemia | Men | 67 | 33 | 1.00 | 34 | 2.15 (1.18-3.91) | 0.01 |
|  | Women | 73 | 33 | 1.00 | 40 | 1.27 (0.65-2.50) | 0.49 |
| Viral hepatitis | Men | 16 | 9 | 1.00 | 7 | 2.35 (0.70-7.92) | 0.17 |
|  | Women | 12 | 9 | 1.00 | 3 | 0.54 (0.12-2.53) | 0.44 |
| Human immunodeficiency virus | Men | 49 | 35 | 1.00 | 14 | 1.29 (0.46-3.58) | 0.63 |
|  | Women | 23 | 12 | 1.00 | 11 | 2.00 (0.59-6.78) | 0.26 |
| Other infectious parasitic disease | Men | 19 | 9 | 1.00 | 10 | 2.25 (0.79-6.42) | 0.13 |
|  | Women | 12 | 4 | 1.00 | 8 | 3.81 (0.73-19.90) | 0.11 |
| Cancers |  |  |  |  |  |  |  |
| All cancers | Men | 1139 | 701 | 1.00 | 438 | 1.11 (0.96-1.28) | 0.16 |
|  | Women | 1171 | 646 | 1.00 | 525 | 0.99 (0.86-1.14) | 0.86 |
| Oral cavity, pharynx, lip | Men | 19 | 12 | 1.00 | 7 | 0.98 (0.36-2.63) | 0.96 |
|  | Women | 8 | 5 | 1.00 | 3 | 0.73 (0.18-2.95) | 0.66 |
| Esophagus | Men | 50 | 28 | 1.00 | 22 | 1.28 (0.64-2.54) | 0.49 |
|  | Women | 5 | 3 | 1.00 | 2 | 2.59 (0.34-19.58) | 0.36 |
| Stomach | Men | 27 | 17 | 1.00 | 10 | 0.92 (0.33-2.51) | 0.87 |
|  | Women | 26 | 15 | 1.00 | 11 | 0.93 (0.38-2.27) | 0.87 |
| Colon, rectum, anus | Men | 92 | 63 | 1.00 | 29 | 0.92 (0.56-1.53) | 0.76 |
|  | Women | 123 | 62 | 1.00 | 61 | 1.14 (0.72-1.78) | 0.58 |
| Liver and bile ducts | Men | 44 | 26 | 1.00 | 18 | 1.04 (0.49-2.23) | 0.91 |
|  | Women | 24 | 16 | 1.00 | 8 | 0.43 (0.14-1.33) | 0.14 |
| Pancreas | Men | 72 | 47 | 1.00 | 25 | 0.88 (0.47-1.65) | 0.70 |
|  | Women | 78 | 34 | 1.00 | 44 | 1.29 (0.76-2.20) | 0.35 |
| Larynx | Men | 14 | 8 | 1.00 | 6 | 1.36 (0.50-3.71) | 0.55 |
|  | Women | 6 | 5 | 1.00 | 1 | 0.13 (0.01-1.83) | 0.13 |
| Lung, trachea, bronchus | Men | 402 | 241 | 1.00 | 161 | 1.30 (1.01-1.66) | 0.04 |
|  | Women | 331 | 183 | 1.00 | 148 | 1.09 (0.83-1.42) | 0.54 |
| Malignant melanoma | Men | 18 | 14 | 1.00 | 4 | 0.30 (0.08-1.15) | 0.08 |
|  | Women | 7 | 3 | 1.00 | 4 | 2.54 (0.25-26.09) | 0.43 |
| Breast (females) | Women | 126 | 78 | 1.00 | 48 | 0.83 (0.52-1.30) | 0.41 |
| Cervix uteri (females) | Women | 19 | 11 | 1.00 | 8 | 0.88 (0.32-2.40) | 0.80 |
| Uterus (females) | Women | 32 | 23 | 1.00 | 9 | 0.37 (0.15-0.90) | 0.03 |
| Ovaries (females) | Women | 61 | 35 | 1.00 | 26 | 0.88 (0.50-1.55) | 0.66 |
| Prostate (males) | Men | 86 | 49 | 1.00 | 37 | 0.89 (0.55-1.44) | 0.63 |
| Kidney and renal pelvis | Men | 38 | 21 | 1.00 | 17 | 1.98 (0.87-4.52) | 0.10 |
|  | Women | 20 | 11 | 1.00 | 9 | 0.71 (0.23-2.24) | 0.56 |
| Bladder | Men | 25 | 16 | 1.00 | 9 | 0.98 (0.38-2.56) | 0.97 |
|  | Women | 23 | 19 | 1.00 | 4 | 0.23 (0.07-0.77) | 0.02 |
| Brain, nervous system | Men | 16 | 12 | 1.00 | 4 | 0.79 (0.23-2.68) | 0.70 |
|  | Women | 26 | 11 | 1.00 | 15 | 1.88 (0.74-4.78) | 0.19 |
| Hodgkin's disease | Men | 3 | 2 | 1.00 | 1 | 1.04 (0.06-16.84) | 0.98 |
|  | Women | 5 | 4 | 1.00 | 1 | 0.90 (0.19-4.26) | 0.90 |
| Non-Hodgkin's lymphoma | Men | 27 | 13 | 1.00 | 14 | 2.24 (0.88-5.68) | 0.09 |
|  | Women | 40 | 21 | 1.00 | 19 | 0.83 (0.42-1.64) | 0.59 |
| Leukemia | Men | 46 | 32 | 1.00 | 14 | 0.70 (0.34-1.45) | 0.34 |
|  | Women | 42 | 25 | 1.00 | 17 | 0.84 (0.42-1.71) | 0.64 |
| Multiple myeloma | Men | 19 | 12 | 1.00 | 7 | 0.63 (0.25-1.61) | 0.33 |
|  | Women | 18 | 8 | 1.00 | 10 | 1.02 (0.36-2.90) | 0.97 |
| All other and unspecified neoplasms | Men | 138 | 86 | 1.00 | 52 | 1.15 (0.77-1.73) | 0.50 |
|  | Women | 151 | 74 | 1.00 | 77 | 1.25 (0.80-1.94) | 0.33 |
| In situ and benign neoplasms | Men | 19 | 13 | 1.00 | 6 | 0.66 (0.18-2.42) | 0.53 |
|  | Women | 32 | 17 | 1.00 | 15 | 0.76 (0.34-1.71) | 0.51 |
| Endocrine, nutritional, metabolic diseases |  |  |  |  |  |  |  |
| Anaemia | Men | 8 | 4 | 1.00 | 4 | 3.46 (0.62-19.23) | 0.16 |
|  | Women | 9 | 6 | 1.00 | 3 | 0.13 (0.03-0.53) | 0.005 |
| Diabetes mellitus | Men | 129 | 42 | 1.00 | 87 | 2.91 (1.85-4.57) | <0.0001 |
|  | Women | 190 | 71 | 1.00 | 119 | 1.41 (0.92-2.16) | 0.11 |
| Malnutrition | Men | 3 | 2 | 1.00 | 1 | 0.36 (0.04-3.25) | 0.36 |
|  | Women | 11 | 7 | 1.00 | 4 | - | - |
| Nervous system |  |  |  |  |  |  |  |
| Parkinson's disease | Men | 36 | 22 | 1.00 | 14 | 1.05 (0.49-2.25) | 0.89 |
|  | Women | 25 | 12 | 1.00 | 13 | - | - |
| Alzheimer's disease | Men | 58 | 41 | 1.00 | 17 | 0.61 (0.31-1.19) | 0.14 |
|  | Women | 149 | 87 | 1.00 | 62 | 0.68 (0.46-1.01) | 0.054 |
| Circulatory disease |  |  |  |  |  |  |  |
| All circulatory diseases | Men | 1657 | 882 | 1.00 | 775 | 1.47 (1.31-1.65) | <0.0001 |
|  | Women | 2164 | 874 | 1.00 | 1290 | 1.47 (1.32-1.64) | <0.0001 |
| Hypertensive heart disease | Men | 59 | 21 | 1.00 | 38 | 4.64 (2.24-9.60) | <0.0001 |
|  | Women | 75 | 25 | 1.00 | 50 | 2.28 (1.25-4.16) | 0.007 |
| Hypertensive heart and renal disease | Men | 5 | 1 | 1.00 | 4 | 2.87 (0.36-22.86) | 0.32 |
|  | Women | 12 | 3 | 1.00 | 9 | 1.83 (0.41-8.28) | 0.43 |
| Ischaemic heart disease | Men | 1005 | 554 | 1.00 | 451 | 1.31 (1.14-1.52) | <0.0001 |
|  | Women | 1045 | 437 | 1.00 | 608 | 1.34 (1.15-1.56) | <0.0001 |
| Acute myocardial infarction | Men | 389 | 200 | 1.00 | 189 | 1.55 (1.24-1.95) | <0.0001 |
|  | Women | 388 | 159 | 1.00 | 229 | 1.39 (1.05-1.82) | 0.02 |
| Other acute ischaemic heart disease | Men | 10 | 5 | 1.00 | 5 | - | - |
|  | Women | 5 | 0 | 1.00 | 5 | - | - |
| Atherosclerotic cardiovascular disease | Men | 168 | 114 | 1.00 | 54 | 0.69 (0.47-1.01) | 0.06 |
|  | Women | 133 | 61 | 1.00 | 72 | 1.33 (0.83-2.13) | 0.24 |
| Other chronic ischaemic heart disease | Men | 438 | 235 | 1.00 | 203 | 1.38 (1.11-1.70) | 0.004 |
|  | Women | 519 | 217 | 1.00 | 302 | 1.28 (1.04-1.59) | 0.02 |
| Heart failure | Men | 78 | 48 | 1.00 | 30 | 0.87 (0.50-1.51) | 0.62 |
|  | Women | 157 | 62 | 1.00 | 95 | 1.43 (0.96-2.11) | 0.08 |
| All other forms of heart disease | Men | 190 | 101 | 1.00 | 89 | 1.55 (1.12-2.15) | 0.008 |
|  | Women | 290 | 125 | 1.00 | 165 | 1.46 (1.09-1.95) | 0.01 |
| Primary hypertension, renal disease | Men | 35 | 11 | 1.00 | 24 | 5.31 (2.25-12.54) | <0.0001 |
|  | Women | 80 | 25 | 1.00 | 55 | 2.07 (1.10-3.90) | 0.03 |
| Cerebrovascular disease | Men | 223 | 117 | 1.00 | 106 | 1.54 (1.16-2.06) | 0.003 |
|  | Women | 395 | 149 | 1.00 | 246 | 1.71 (1.45-2.19) | <0.0001 |
| Atherosclerosis | Men | 10 | 5 | 1.00 | 5 | 2.76 (0.75-10.21) | 0.13 |
|  | Women | 20 | 9 | 1.00 | 11 | 0.99 (0.33-2.96) | 0.99 |
| Other diseases of circulatory system | Men | 52 | 24 | 1.00 | 28 | 1.95 (0.91-4.17) | 0.08 |
|  | Women | 90 | 39 | 1.00 | 51 | 1.43 (0.80-2.55) | 0.23 |
| Aortic aneurysm and dissection | Men | 34 | 14 | 1.00 | 20 | 2.25 (0.95-5.29) | 0.06 |
|  | Women | 40 | 18 | 1.00 | 22 | 0.95 (0.45-2.00) | 0.90 |
| Other diseases of arteries or capillaries | Men | 13 | 8 | 1.00 | 5 | 1.33 (0.22-8.17) | 0.76 |
|  | Women | 35 | 14 | 1.00 | 21 | 1.95 (0.72-5.27) | 0.19 |
| Other disorders of circulatory system | Men | 5 | 2 | 1.00 | 3 | 4.47 (0.16-124.71) | 0.38 |
|  | Women | 15 | 7 | 1.00 | 8 | 2.47 (0.58-10.57) | 0.22 |
| Respiratory diseases |  |  |  |  |  |  |  |
| Pneumonia | Men | 90 | 56 | 1.00 | 34 | 0.79 (0.37-1.65) | 0.52 |
|  | Women | 149 | 81 | 1.00 | 68 | 0.87 (0.60-1.26) | 0.47 |
| Emphysema | Men | 32 | 19 | 1.00 | 13 | 1.24 (0.55-2.80) | 0.61 |
|  | Women | 30 | 17 | 1.00 | 13 | 1.18 (0.52-2.71) | 0.69 |
| Other chronic lower respiratory disease | Men | 226 | 137 | 1.00 | 89 | 1.23 (0.89-1.69) | 0.22 |
|  | Women | 266 | 121 | 1.00 | 145 | 1.56 (1.13-2.16) | 0.007 |
| Pneumonitis from solids, liquids | Men | 39 | 26 | 1.00 | 13 | 0.55 (0.26-1.20) | 0.13 |
|  | Women | 35 | 21 | 1.00 | 14 | 1.01 (0.40-2.57) | 0.98 |
| Other respiratory system diseases | Men | 56 | 38 | 1.00 | 18 | 0.79 (0.37-1.65) | 0.52 |
|  | Women | 85 | 36 | 1.00 | 49 | 1.65 (1.01-2.69) | 0.045 |
| Digestive diseases |  |  |  |  |  |  |  |
| Alcoholic liver disease | Men | 63 | 41 | 1.00 | 22 | 1.86 (0.90-3.82) | 0.09 |
|  | Women | 17 | 10 | 1.00 | 7 | 1.03 (0.33-3.23) | 0.96 |
| Other chronic liver disease | Men | 45 | 28 | 1.00 | 17 | 1.33 (0.63-2.82) | 0.46 |
|  | Women | 42 | 18 | 1.00 | 24 | 2.99 (1.08-8.31) | 0.04 |
| Cholelithiasis, gallbladder disease | Men | 4 | 2 | 1.00 | 2 | 0.92 (0.10-8.72) | 0.94 |
|  | Women | 10 | 3 | 1.00 | 7 | 2.05 (0.30-14.12) | 0.47 |
| Urinary tract disease |  |  |  |  |  |  |  |
| Kidney failure | Men | 75 | 36 | 1.00 | 39 | 1.73 (1.03-2.89) | 0.04 |
|  | Women | 111 | 35 | 1.00 | 76 | 2.06 (1.23-3.42) | 0.006 |
| Abnormal clinical, lab findings | Men | 45 | 30 | 1.00 | 15 | 0.78 (0.39-1.55) | 0.48 |
|  | Women | 71 | 35 | 1.00 | 36 | 1.47 (0.83-2.60) | 0.19 |
| Transport injuries |  |  |  |  |  |  |  |
| Motor vehicle accidents | Men | 101 | 77 | 1.00 | 24 | 1.68 (1.01-2.79) | 0.04 |
|  | Women | 72 | 49 | 1.00 | 23 | 1.45 (0.76-2.77) | 0.26 |
| Unintentional injuries |  |  |  |  |  |  |  |
| Falls | Men | 39 | 26 | 1.00 | 13 | 1.54 (0.63-3.74) | 0.34 |
|  | Women | 42 | 25 | 1.00 | 17 | 0.62 (0.29-1.31) | 0.21 |
| Other non-transport accidents combined | Men | 110 | 85 | 1.00 | 25 | 1.07 (0.61-1.85) | 0.82 |
|  | Women | 76 | 45 | 1.00 | 31 | 1.34 (0.80-2.24) | 0.27 |
| Self-harm, interpersonal violence |  |  |  |  |  |  |  |
| Suicide | Men | 121 | 92 | 1.00 | 29 | 1.01 (0.55-1.83) | 0.98 |
|  | Women | 30 | 23 | 1.00 | 7 | 1.24 (0.44-3.46) | 0.68 |
| Homicide | Men | 31 | 28 | 1.00 | 3 | 0.25 (0.07-0.89) | 0.03 |
|  | Women | 25 | 18 | 1.00 | 7 | 1.05 (0.34-3.25) | 0.93 |
| All other diseases (residual) | Men | 364 | 215 | 1.00 | 149 | 1.14 (0.88-1.49) | 0.32 |
|  | Women | 546 | 249 | 1.00 | 297 | 1.41 (1.14-1.75) | 0.002 |
| All other causes/all unknown causes | Men | 584 | 450 | 1.00 | 134 | 1.08 (0.85-1.38) | 0.52 |
|  | Women | 551 | 376 | 1.00 | 175 | 0.92 (0.73-1.15) | 0.44 |

Multivariable adjustment for age, sex, education, race, income, alcohol, smoking status, BMI, physical activity, and survey year

Supplementary Table 5. Hazard ratios of all-cause mortality and cause-specific mortality among participants with hypertension compared to participants without hypertension stratified by race/ethnicity (Whites = non-Hispanic whites, others = Hispanic, non-Hispanic black, non-Hispanic other)

|  |  | Total | No hypertension | | Hypertension | |  |
| --- | --- | --- | --- | --- | --- | --- | --- |
|  | Race/ ethnicity | N  (deaths) | N  (deaths) | HR | N (deaths) | HR (95% CI) | P-value |
| All-cause mortality | Whites | 7775 | 4224 | 1.00 | 3551 | 1.25 (1.18-1.32) | <0.0001 |
|  | Others | 3479 | 1895 | 1.00 | 1584 | 1.30 (1.18-1.44) | <0.0001 |
| Infections |  |  |  |  |  |  |  |
| Septicemia | Whites | 94 | 46 | 1.00 | 48 | 1.52 (0.92-2.52) | 0.11 |
|  | Others | 46 | 20 | 1.00 | 26 | 2.08 (0.84-5.19) | 0.12 |
| Viral hepatitis | Whites | 18 | 12 | 1.00 | 6 | 1.56 (0.45-5.40) | 0.49 |
|  | Others | 10 | 6 | 1.00 | 4 | 1.28 (0.25-6.48) | 0.77 |
| Human immunodeficiency virus | Whites | 15 | 10 | 1.00 | 5 | 4.59 (1.27-16.52) | 0.02 |
|  | Others | 57 | 37 | 1.00 | 20 | 1.07 (0.42-2.73) | 0.89 |
| Other infectious parasitic disease | Whites | 20 | 10 | 1.00 | 10 | 2.21 (0.79-6.18) | 0.13 |
|  | Others | 11 | 3 | 1.00 | 8 | 5.16 (0.99-26.89) | 0.05 |
| Cancers |  |  |  |  |  |  |  |
| All cancers | Whites | 1652 | 981 | 1.00 | 671 | 1.07 (0.95-1.21) | 0.25 |
|  | Others | 658 | 366 | 1.00 | 292 | 0.98 (0.80-1.19) | 0.81 |
| Oral cavity, pharynx, lip | Whites | 21 | 12 | 1.00 | 9 | 1.11 (0.46-2.69) | 0.82 |
|  | Others | 6 | 5 | 1.00 | 1 | 0.20 (0.02-2.53) | 0.21 |
| Esophagus | Whites | 35 | 20 | 1.00 | 15 | 1.45 (0.66-3.20) | 0.35 |
|  | Others | 20 | 11 | 1.00 | 9 | 1.10 (0.36-3.34) | 0.87 |
| Stomach | Whites | 32 | 19 | 1.00 | 13 | 1.28 (0.57-2.87) | 0.55 |
|  | Others | 21 | 13 | 1.00 | 8 | 0.50 (0.17-1.49) | 0.21 |
| Colon, rectum, anus | Whites | 150 | 85 | 1.00 | 65 | 1.09 (0.74-1.59) | 0.66 |
|  | Others | 65 | 40 | 1.00 | 25 | 1.03 (0.53-2.00) | 0.94 |
| Liver and bile ducts | Whites | 39 | 25 | 1.00 | 14 | 0.93 (0.44-1.98) | 0.86 |
|  | Others | 29 | 17 | 1.00 | 12 | 0.70 (0.22-2.24) | 0.55 |
| Pancreas | Whites | 101 | 57 | 1.00 | 44 | 1.05 (0.65-1.71) | 0.84 |
|  | Others | 49 | 24 | 1.00 | 25 | 1.04 (0.51-2.13) | 0.91 |
| Larynx | Whites | 15 | 10 | 1.00 | 5 | 0.99 (0.34-2.91) | 0.98 |
|  | Others | 5 | 3 | 1.00 | 2 | - | - |
| Lung, trachea, bronchus | Whites | 554 | 333 | 1.00 | 221 | 1.15 (0.93-1.42) | 0.20 |
|  | Others | 179 | 91 | 1.00 | 88 | 1.52 (1.01-2.27) | 0.04 |
| Malignant melanoma | Whites | 23 | 16 | 1.00 | 7 | 0.59 (0.18-1.92) | 0.38 |
|  | Others | 2 | 1 | 1.00 | 1 | - | - |
| Breast (females) | Whites | 72 | 47 | 1.00 | 25 | 0.76 (0.42-1.40) | 0.38 |
|  | Others | 56 | 32 | 1.00 | 24 | 1.01 (0.59-1.73) | 0.97 |
| Cervix uteri (females) | Whites | 12 | 9 | 1.00 | 3 | 0.50 (0.11-2.27) | 0.37 |
|  | Others | 7 | 2 | 1.00 | 5 | 2.16 (0.36-13.02) | 0.40 |
| Uterus (females) | Whites | 19 | 15 | 1.00 | 4 | 0.22 (0.07-0.68) | 0.009 |
|  | Others | 13 | 8 | 1.00 | 5 | 0.98 (0.34-2.79) | 0.97 |
| Ovaries (females) | Whites | 50 | 29 | 1.00 | 21 | 0.94 (0.51-1.76) | 0.86 |
|  | Others | 12 | 7 | 1.00 | 5 | - | - |
| Prostate (males) | Whites | 50 | 30 | 1.00 | 20 | 1.00 (0.55-1.83) | 0.99 |
|  | Others | 36 | 19 | 1.00 | 17 | 0.74 (0.34-1.60) | 0.44 |
| Kidney and renal pelvis | Whites | 40 | 21 | 1.00 | 19 | 1.40 (0.64-3.10) | 0.40 |
|  | Others | 18 | 11 | 1.00 | 7 | 1.68 (0.39-7.20) | 0.48 |
| Bladder | Whites | 43 | 31 | 1.00 | 12 | 0.46 (0.22-0.98) | 0.04 |
|  | Others | 5 | 4 | 1.00 | 1 | - | - |
| Brain, nervous system | Whites | 36 | 20 | 1.00 | 16 | 1.47 (0.67-3.22) | 0.34 |
|  | Others | 6 | 3 | 1.00 | 3 | 0.84 (0.24-2.93) | 0.79 |
| Hodgkin's disease | Whites | 7 | 5 | 1.00 | 2 | 0.90 (0.17-4.61) | 0.90 |
|  | Others | 1 | 1 | 1.00 | 0 | - | - |
| Non-Hodgkin's lymphoma | Whites | 55 | 27 | 1.00 | 28 | 1.70 (0.88-3.27) | 0.11 |
|  | Others | 12 | 7 | 1.00 | 5 | 0.66 (0.23-1.92) | 0.44 |
| Leukemia | Whites | 72 | 45 | 1.00 | 27 | 0.84 (0.48-1.48) | 0.55 |
|  | Others | 16 | 12 | 1.00 | 4 | 0.41 (0.11-1.53) | 0.18 |
| Multiple myeloma | Whites | 20 | 11 | 1.00 | 9 | 0.85 (0.37-1.96) | 0.71 |
|  | Others | 17 | 9 | 1.00 | 8 | 0.70 (0.26-1.91) | 0.49 |
| All other and unspecified neoplasms | Whites | 206 | 114 | 1.00 | 92 | 1.30 (0.93-1.84) | 0.13 |
|  | Others | 83 | 46 | 1.00 | 37 | 0.84 (0.48-1.47) | 0.53 |
| In situ and benign neoplasms | Whites | 36 | 24 | 1.00 | 12 | 0.66 (0.33-1.32) | 0.24 |
|  | Others | 15 | 6 | 1.00 | 9 | 1.03 (0.27-3.90) | 0.97 |
| Endocrine, nutritional, metabolic diseases |  |  |  |  |  |  |  |
| Anaemia | Whites | 7 | 4 | 1.00 | 3 | 0.93 (0.13-6.88) | 0.95 |
|  | Others | 10 | 6 | 1.00 | 4 | 1.10 (0.15-8.04) | 0.93 |
| Diabetes mellitus | Whites | 188 | 67 | 1.00 | 121 | 2.04 (1.38-3.02) | <0.0001 |
|  | Others | 131 | 46 | 1.00 | 85 | 1.73 (1.07-2.80) | 0.03 |
| Malnutrition | Whites | 11 | 7 | 1.00 | 4 | 0.39 (0.11-1.41) | 0.15 |
|  | Others | 3 | 2 | 1.00 | 1 | 0.07 (0.02-0.22) | <0.0001 |
| Nervous system |  |  |  |  |  |  |  |
| Parkinson's disease | Whites | 53 | 32 | 1.00 | 21 | 1.06 (0.57-1.96) | 0.86 |
|  | Others | 8 | 2 | 1.00 | 6 | 2.69 (0.61-11.87) | 0.19 |
| Alzheimer's disease | Whites | 177 | 115 | 1.00 | 62 | 0.55 (0.39-0.78) | 0.001 |
|  | Others | 30 | 13 | 1.00 | 17 | 2.17 (0.77-6.13) | 0.14 |
| Circulatory disease |  |  |  |  |  |  |  |
| All circulatory diseases | Whites | 2772 | 1307 | 1.00 | 1465 | 1.46 (1.34-1.59) | <0.0001 |
|  | Others | 1049 | 449 | 1.00 | 600 | 1.63 (1.35-1.96) | <0.0001 |
| Hypertensive heart disease | Whites | 81 | 25 | 1.00 | 56 | 3.63 (2.01-6.54) | <0.0001 |
|  | Others | 53 | 21 | 1.00 | 32 | 2.54 (1.17-5.51) | 0.02 |
| Hypertensive heart and renal disease | Whites | 9 | 3 | 1.00 | 6 | - | - |
|  | Others | 8 | 1 | 1.00 | 7 | 2.89 (0.18-46.14) | 0.45 |
| Ischaemic heart disease | Whites | 1457 | 720 | 1.00 | 737 | 1.33 (1.19-1.48) | <0.0001 |
|  | Others | 593 | 271 | 1.00 | 322 | 1.44 (1.15-1.82) | 0.002 |
| Acute myocardial infarction | Whites | 568 | 275 | 1.00 | 293 | 1.44 (1.21-1.72) | <0.0001 |
|  | Others | 209 | 84 | 1.00 | 125 | 1.73 (1.09-2.74) | 0.02 |
| Other acute ischaemic heart disease | Whites | 12 | 5 | 1.00 | 7 | 1.58 (0.53-4.72) | 0.42 |
|  | Others | 3 | 0 | 1.00 | 3 | - | - |
| Atherosclerotic cardiovascular disease | Whites | 190 | 115 | 1.00 | 75 | 0.97 (0.68-1.38) | 0.88 |
|  | Others | 111 | 60 | 1.00 | 51 | 0.95 (0.57-1.60) | 0.86 |
| Other chronic ischaemic heart disease | Whites | 687 | 325 | 1.00 | 362 | 1.34 (1.14-1.58) | <0.0001 |
|  | Others | 270 | 127 | 1.00 | 143 | 1.40 (1.02-1.93) | 0.04 |
| Heart failure | Whites | 188 | 88 | 1.00 | 100 | 1.23 (0.88-1.72) | 0.22 |
|  | Others | 47 | 22 | 1.00 | 25 | 0.91 (0.43-1.94) | 0.82 |
| All other forms of heart disease | Whites | 364 | 180 | 1.00 | 184 | 1.47 (1.15-1.88) | 0.002 |
|  | Others | 116 | 46 | 1.00 | 70 | 1.72 (1.07-2.77) | 0.03 |
| Primary hypertension, renal disease | Whites | 83 | 25 | 1.00 | 58 | 2.93 (1.60-5.36) | 0.001 |
|  | Others | 32 | 11 | 1.00 | 21 | 3.85 (1.30-11.42) | 0.02 |
| Cerebrovascular disease | Whites | 453 | 202 | 1.00 | 251 | 1.63 (1.32-2.01) | <0.0001 |
|  | Others | 165 | 64 | 1.00 | 101 | 1.76 (1.17-2.65) | 0.007 |
| Atherosclerosis | Whites | 26 | 14 | 1.00 | 12 | 1.20 (0.46-3.14) | 0.71 |
|  | Others | 4 | 0 | 1.00 | 4 | - | - |
| Other diseases of circulatory system | Whites | 111 | 50 | 1.00 | 61 | 1.58 (0.97-2.59) | 0.07 |
|  | Others | 31 | 13 | 1.00 | 18 | 2.48 (0.75-8.20) | 0.14 |
| Aortic aneurysm and dissection | Whites | 60 | 28 | 1.00 | 32 | 1.29 (0.71-2.34) | 0.41 |
|  | Others | 14 | 4 | 1.00 | 10 | 4.79 (0.84-27.37) | 0.08 |
| Other diseases of arteries or capillaries | Whites | 39 | 18 | 1.00 | 21 | 1.92 (0.75-4.92) | 0.17 |
|  | Others | 9 | 4 | 1.00 | 5 | 0.58 (0.13-2.61) | 0.48 |
| Other disorders of circulatory system | Whites | 12 | 4 | 1.00 | 8 | 3.25 (0.53-19.78) | 0.20 |
|  | Others | 8 | 5 | 1.00 | 3 | 2.07 (0.21-20.33) | 0.53 |
| Respiratory diseases |  |  |  |  |  |  |  |
| Pneumonia | Whites | 169 | 106 | 1.00 | 63 | 0.84 (0.59-1.18) | 0.31 |
|  | Others | 70 | 31 | 1.00 | 39 | 1.39 (0.76-2.54) | 0.28 |
| Emphysema | Whites | 52 | 31 | 1.00 | 21 | 1.19 (0.63-2.24) | 0.59 |
|  | Others | 10 | 5 | 1.00 | 5 | 1.23 (0.27-5.58) | 0.79 |
| Other chronic lower respiratory disease | Whites | 429 | 231 | 1.00 | 198 | 1.37 (1.09-1.72) | 0.007 |
|  | Others | 63 | 27 | 1.00 | 36 | 1.68 (0.86-3.27) | 0.13 |
| Pneumonitis from solids, liquids | Whites | 64 | 40 | 1.00 | 24 | 0.83 (0.46-1.49) | 0.52 |
|  | Others | 10 | 7 | 1.00 | 3 | 0.19 (0.04-0.79) | 0.02 |
| Other respiratory system diseases | Whites | 104 | 60 | 1.00 | 44 | 0.99 (0.64-1.53) | 0.96 |
|  | Others | 37 | 14 | 1.00 | 23 | 2.62 (0.79-8.71) | 0.12 |
| Digestive diseases |  |  |  |  |  |  |  |
| Alcoholic liver disease | Whites | 44 | 30 | 1.00 | 14 | 1.26 (0.57-2.83) | 0.57 |
|  | Others | 36 | 21 | 1.00 | 15 | 2.67 (0.95-7.49) | 0.06 |
| Other chronic liver disease | Whites | 65 | 34 | 1.00 | 31 | 2.00 (1.03-3.90) | 0.04 |
|  | Others | 22 | 12 | 1.00 | 10 | 1.33 (0.42-4.28) | 0.63 |
| Cholelithiasis, gallbladder disease | Whites | 11 | 3 | 1.00 | 8 | 1.73 (0.39-7.72) | 0.47 |
|  | Others | 3 | 2 | 1.00 | 1 | 0.32 (0.02-5.61) | 0.43 |
| Urinary tract disease |  |  |  |  |  |  |  |
| Kidney failure | Whites | 124 | 53 | 1.00 | 71 | 1.58 (1.06-2.36) | 0.03 |
|  | Others | 62 | 18 | 1.00 | 44 | 3.87 (1.84-8.14) | <0.0001 |
| Abnormal clinical, lab findings | Whites | 91 | 51 | 1.00 | 40 | 1.13 (0.72-1.77) | 0.60 |
|  | Others | 25 | 14 | 1.00 | 11 | 1.23 (0.55-2.74) | 0.61 |
| Transport injuries |  |  |  |  |  |  |  |
| Motor vehicle accidents | Whites | 119 | 84 | 1.00 | 35 | 1.77 (1.12-2.78) | 0.01 |
|  | Others | 54 | 42 | 1.00 | 12 | 1.12 (0.48-2.61) | 0.79 |
| Unintentional injuries |  |  |  |  |  |  |  |
| Falls | Whites | 69 | 43 | 1.00 | 26 | 1.04 (0.54-2.04) | 0.90 |
|  | Others | 12 | 8 | 1.00 | 4 | 0.73 (0.15-3.59) | 0.70 |
| Other non-transport accidents combined | Whites | 120 | 86 | 1.00 | 34 | 1.18 (0.73-1.90) | 0.51 |
|  | Others | 66 | 44 | 1.00 | 22 | 1.25 (0.74-2.11) | 0.41 |
| Self-harm, interpersonal violence |  |  |  |  |  |  |  |
| Suicide | Whites | 120 | 88 | 1.00 | 32 | 1.33 (0.76-2.32) | 0.32 |
|  | Others | 31 | 27 | 1.00 | 4 | 0.26 (0.07-0.95) | 0.04 |
| Homicide | Whites | 19 | 17 | 1.00 | 2 | 0.19 (0.04-0.79) | 0.02 |
|  | Others | 37 | 29 | 1.00 | 8 | 1.12 (0.41-3.06) | 0.82 |
| All other diseases (residual) | Whites | 652 | 344 | 1.00 | 308 | 1.31 (1.08-1.59) | 0.007 |
|  | Others | 258 | 120 | 1.00 | 138 | 1.32 (0.96-1.81) | 0.09 |
| All other causes/all unknown causes | Whites | 480 | 308 | 1.00 | 172 | 1.05 (0.84-1.31) | 0.68 |
|  | Others | 655 | 518 | 1.00 | 137 | 0.97 (0.76-1.23) | 0.80 |

Multivariable adjustment for age, sex, education, race, income, alcohol, smoking status, BMI, physical activity, and survey year

Supplementary Table 6. Hazard ratios of all-cause mortality and cause-specific mortality among participants with hypertension compared to participants without hypertension stratified by education (low = less than high school degree, medium = high school degree, high = more than high school degree)

|  |  | Total | No hypertension | | Hypertension | |  |
| --- | --- | --- | --- | --- | --- | --- | --- |
|  | Education | N  (deaths) | N  (deaths) | HR | N (deaths) | HR (95% CI) | P-value |
| All-cause mortality | Low/medium | 7742 | 4097 | 1.00 | 3645 | 1.20 (1.14-1.27) | <0.0001 |
|  | High | 3369 | 1939 | 1.00 | 1430 | 1.37 (1.25-1.50) | <0.0001 |
| Infections |  |  |  |  |  |  |  |
| Septicemia | Low/medium | 96 | 45 | 1.00 | 51 | 1.59 (0.93-2.72) | 0.09 |
|  | High | 41 | 18 | 1.00 | 23 | 1.98 (0.92-4.25) | 0.08 |
| Viral hepatitis | Low/medium | 16 | 11 | 1.00 | 5 | 1.51 (0.30-7.64) | 0.62 |
|  | High | 12 | 7 | 1.00 | 5 | 1.65 (0.57-4.76) | 0.36 |
| Human immunodeficiency virus | Low/medium | 46 | 30 | 1.00 | 16 | 1.02 (0.42-2.50) | 0.96 |
|  | High | 25 | 16 | 1.00 | 9 | 2.56 (0.73-8.96) | 0.14 |
| Other infectious parasitic disease | Low/medium | 21 | 7 | 1.00 | 14 | 3.71 (1.18-11.66) | 0.03 |
|  | High | 9 | 6 | 1.00 | 3 | 1.40 (0.31-6.26) | 0.66 |
| Cancers |  |  |  |  |  |  |  |
| All cancers | Low/medium | 1543 | 870 | 1.00 | 673 | 1.03 (0.91-1.16) | 0.67 |
|  | High | 751 | 466 | 1.00 | 285 | 1.11 (0.93-1.33) | 0.24 |
| Oral cavity, pharynx, lip | Low/medium | 17 | 10 | 1.00 | 7 | 1.24 (0.41-3.71) | 0.70 |
|  | High | 9 | 6 | 1.00 | 3 | 0.62 (0.16-2.39) | 0.49 |
| Esophagus | Low/medium | 36 | 21 | 1.00 | 15 | 1.17 (0.51-2.69) | 0.71 |
|  | High | 19 | 10 | 1.00 | 9 | 1.77 (0.64-4.88) | 0.27 |
| Stomach | Low/medium | 37 | 24 | 1.00 | 13 | 0.57 (0.26-1.25) | 0.16 |
|  | High | 16 | 8 | 1.00 | 8 | 2.34 (0.80-6.84) | 0.12 |
| Colon, rectum, anus | Low/medium | 144 | 81 | 1.00 | 63 | 1.01 (0.67-1.52) | 0.97 |
|  | High | 68 | 42 | 1.00 | 26 | 1.25 (0.72-2.15) | 0.43 |
| Liver and bile ducts | Low/medium | 40 | 23 | 1.00 | 17 | 0.87 (0.39-1.92) | 0.73 |
|  | High | 27 | 18 | 1.00 | 9 | 0.80 (0.31-2.08) | 0.65 |
| Pancreas | Low/medium | 104 | 51 | 1.00 | 53 | 1.10 (0.66-1.84) | 0.72 |
|  | High | 45 | 30 | 1.00 | 15 | 0.85 (0.41-1.77) | 0.67 |
| Larynx | Low/medium | 14 | 11 | 1.00 | 3 | 0.41 (0.09-1.899) | 0.25 |
|  | High | 6 | 2 | 1.00 | 4 | 2.89 (0.46-17.96) | 0.25 |
| Lung, trachea, bronchus | Low/medium | 508 | 286 | 1.00 | 222 | 1.21 (0.96-1.51) | 0.10 |
|  | High | 222 | 135 | 1.00 | 87 | 1.24 (0.91-1.68) | 0.18 |
| Malignant melanoma | Low/medium | 12 | 7 | 1.00 | 5 | 0.73 (0.19-2.78) | 0.65 |
|  | High | 13 | 10 | 1.00 | 3 | 0.51 (0.09-2.79) | 0.43 |
| Breast (females) | Low/medium | 84 | 47 | 1.00 | 37 | 1.09 (0.65-1.81) | 0.75 |
|  | High | 43 | 31 | 1.00 | 12 | 0.45 (0.17-1.18) | 0.11 |
| Cervix uteri (females) | Low/medium | 15 | 8 | 1.00 | 7 | 1.01 (0.31-3.25) | 0.98 |
|  | High | 4 | 3 | 1.00 | 1 | 0.43 (0.04-4.13) | 0.46 |
| Uterus (females) | Low/medium | 24 | 16 | 1.00 | 8 | 0.35 (0.14-0.91) | 0.03 |
|  | High | 6 | 6 | 1.00 | 0 | - | - |
| Ovaries (females) | Low/medium | 45 | 25 | 1.00 | 20 | 0.76 (0.40-1.45) | 0.40 |
|  | High | 16 | 10 | 1.00 | 6 | 1.64 (0.53-5.09) | 0.39 |
| Prostate (males) | Low/medium | 61 | 34 | 1.00 | 27 | 0.98 (0.55-1.74) | 0.94 |
|  | High | 25 | 15 | 1.00 | 10 | 0.70 (0.25-1.96) | 0.50 |
| Kidney and renal pelvis | Low/medium | 36 | 20 | 1.00 | 16 | 1.21 (0.46-3.22) | 0.70 |
|  | High | 22 | 12 | 1.00 | 10 | 1.81 (0.67-4.86) | 0.24 |
| Bladder | Low/medium | 32 | 25 | 1.00 | 7 | 0.46 (0.17-1.24) | 0.12 |
|  | High | 16 | 10 | 1.00 | 6 | 0.84 (0.27-2.61) | 0.76 |
| Brain, nervous system | Low/medium | 22 | 13 | 1.00 | 9 | 1.24 (0.47-3.24) | 0.66 |
|  | High | 20 | 10 | 1.00 | 10 | 1.50 (0.51-4.38) | 0.46 |
| Hodgkin's disease | Low/medium | 4 | 2 | 1.00 | 2 | - | - |
|  | High | 4 | 4 | 1.00 | 0 | - | - |
| Non-Hodgkin's lymphoma | Low/medium | 41 | 20 | 1.00 | 21 | 1.08 (0.53-2.22) | 0.83 |
|  | High | 25 | 14 | 1.00 | 11 | 2.27 (0.90-5.74) | 0.08 |
| Leukemia | Low/medium | 55 | 35 | 1.00 | 20 | 0.77 (0.42-1.43) | 0.41 |
|  | High | 33 | 22 | 1.00 | 11 | 0.73 (0.29-1.85) | 0.51 |
| Multiple myeloma | Low/medium | 24 | 10 | 1.00 | 14 | 1.04 (0.44-2.44) | 0.93 |
|  | High | 13 | 10 | 1.00 | 3 | 0.45 (0.12-1.72) | 0.24 |
| All other and unspecified neoplasms | Low/medium | 188 | 101 | 1.00 | 87 | 1.12 (0.77-1.63) | 0.54 |
|  | High | 99 | 58 | 1.00 | 41 | 1.40 (0.85-2.29) | 0.19 |
| In situ and benign neoplasms | Low/medium | 36 | 19 | 1.00 | 17 | 0.63 (0.30-1.34) | 0.23 |
|  | High | 15 | 11 | 1.00 | 4 | 0.97 (0.24-3.98) | 0.97 |
| Endocrine, nutritional, metabolic diseases |  |  |  |  |  |  |  |
| Anaemia | Low/medium | 14 | 8 | 1.00 | 6 | 1.01 (0.21-4.86) | 0.99 |
|  | High | 3 | 2 | 1.00 | 1 | - | - |
| Diabetes mellitus | Low/medium | 237 | 79 | 1.00 | 158 | 1.73 (1.24-2.43) | 0.001 |
|  | High | 79 | 32 | 1.00 | 47 | 2.77 (1.60-4.80) | <0.0001 |
| Malnutrition | Low/medium | 9 | 6 | 1.00 | 3 | 0.25 (0.06-1.00) | 0.05 |
|  | High | 5 | 3 | 1.00 | 2 | - | - |
| Nervous system |  |  |  |  |  |  |  |
| Parkinson's disease | Low/medium | 36 | 20 | 1.00 | 16 | 1.07 (0.51-2.23) | 0.87 |
|  | High | 25 | 14 | 1.00 | 11 | 1.35 (0.57-3.23) | 0.50 |
| Alzheimer's disease | Low/medium | 143 | 89 | 1.00 | 54 | 0.61 (0.41-0.91) | 0.02 |
|  | High | 58 | 36 | 1.00 | 22 | 0.68 (0.35-1.30) | 0.24 |
| Circulatory disease |  |  |  |  |  |  |  |
| All circulatory diseases | Low/medium | 2728 | 1218 | 1.00 | 1510 | 1.46 (1.33-1.60) | <0.0001 |
|  | High | 1047 | 513 | 1.00 | 534 | 1.59 (1.36-1.85) | <0.0001 |
| Hypertensive heart disease | Low/medium | 93 | 32 | 1.00 | 61 | 2.83 (1.62-4.93) | <0.0001 |
|  | High | 39 | 14 | 1.00 | 25 | 4.39 (1.87-10.31) | 0.001 |
| Hypertensive heart and renal disease | Low/medium | 14 | 4 | 1.00 | 10 | 1.85 (0.50-6.82) | 0.35 |
|  | High | 3 | 0 | 1.00 | 3 | - | - |
| Ischaemic heart disease | Low/medium | 1472 | 675 | 1.00 | 797 | 1.43 (1.27-1.61) | <0.0001 |
|  | High | 551 | 299 | 1.00 | 252 | 1.23 (1.01-1.51) | 0.04 |
| Acute myocardial infarction | Low/medium | 547 | 247 | 1.00 | 300 | 1.47 (1.20-1.81) | <0.0001 |
|  | High | 220 | 107 | 1.00 | 113 | 1.60 (1.16-2.21) | 0.004 |
| Other acute ischaemic heart disease | Low/medium | 10 | 2 | 1.00 | 8 | 2.86 (0.62-13.31) | 0.18 |
|  | High | 5 | 3 | 1.00 | 2 | - | - |
| Atherosclerotic cardiovascular disease | Low/medium | 206 | 108 | 1.00 | 98 | 1.14 (0.82-1.58) | 0.44 |
|  | High | 91 | 64 | 1.00 | 27 | 0.68 (0.37-1.23) | 0.20 |
| Other chronic ischaemic heart disease | Low/medium | 709 | 318 | 1.00 | 391 | 1.46 (1.23-1.74) | <0.0001 |
|  | High | 235 | 125 | 1.00 | 110 | 1.16 (0.86-1.57) | 0.34 |
| Heart failure | Low/medium | 181 | 82 | 1.00 | 9 | 1.20 (0.84-1.70) | 0.32 |
|  | High | 51 | 27 | 1.00 | 24 | 1.05 (0.55-2.02) | 0.87 |
| All other forms of heart disease | Low/medium | 324 | 155 | 1.00 | 169 | 1.18 (0.91-1.52) | 0.21 |
|  | High | 151 | 68 | 1.00 | 83 | 2.41 (1.64-3.54) | <0.0001 |
| Primary hypertension, renal disease | Low/medium | 97 | 29 | 1.00 | 68 | 3.48 (1.87-6.51) | <0.0001 |
|  | High | 16 | 5 | 1.00 | 11 | 3.17 (0.97-10.36) | 0.06 |
| Cerebrovascular disease | Low/medium | 426 | 188 | 1.00 | 238 | 1.41 (1.13-1.76) | 0.002 |
|  | High | 187 | 77 | 1.00 | 110 | 2.20 (1.60-3.04) | <0.0001 |
| Atherosclerosis | Low/medium | 20 | 9 | 1.00 | 11 | 1.79 (0.67-4.77) | 0.25 |
|  | High | 8 | 4 | 1.00 | 4 | 2.46 (0.46-13.09) | 0.29 |
| Other diseases of circulatory system | Low/medium | 101 | 44 | 1.00 | 57 | 1.74 (1.04-2.91) | 0.03 |
|  | High | 41 | 19 | 1.00 | 22 | 1.61 (0.61-4.22) | 0.33 |
| Aortic aneurysm and dissection | Low/medium | 46 | 20 | 1.00 | 26 | 1.82 (0.87-3.78) | 0.11 |
|  | High | 28 | 12 | 1.00 | 16 | 1.21 (0.46-3.18) | 0.70 |
| Other diseases of arteries or capillaries | Low/medium | 39 | 17 | 1.00 | 22 | 1.40 (0.65-3.02) | 0.39 |
|  | High | 9 | 5 | 1.00 | 4 | 2.88 (0.32-26.27) | 0.35 |
| Other disorders of circulatory system | Low/medium | 16 | 7 | 1.00 | 9 | 3.01 (0.75-12.12) | 0.12 |
|  | High | 4 | 2 | 1.00 | 2 | 1.31 (0.05-35.37) | 0.87 |
| Respiratory diseases |  |  |  |  |  |  |  |
| Pneumonia | Low/medium | 154 | 85 | 1.00 | 69 | 0.89 (0.61-1.31) | 0.56 |
|  | High | 76 | 49 | 1.00 | 27 | 0.90 (0.54-1.49) | 0.68 |
| Emphysema | Low/medium | 42 | 25 | 1.00 | 17 | 0.93 (0.44-1.97) | 0.85 |
|  | High | 19 | 10 | 1.00 | 9 | - | - |
| Other chronic lower respiratory disease | Low/medium | 348 | 188 | 1.00 | 160 | 1.19 (0.92-1.53) | 0.18 |
|  | High | 137 | 66 | 1.00 | 71 | 2.00 (1.35-2.94) | 0.001 |
| Pneumonitis from solids, liquids | Low/medium | 49 | 31 | 1.00 | 18 | 0.63 (0.31-1.27) | 0.19 |
|  | High | 24 | 15 | 1.00 | 9 | 0.96 (0.37-2.50) | 0.93 |
| Other respiratory system diseases | Low/medium | 103 | 55 | 1.00 | 48 | 1.11 (0.67-1.83) | 0.69 |
|  | High | 36 | 18 | 1.00 | 18 | 1.35 (0.60-3.00) | 0.47 |
| Digestive diseases |  |  |  |  |  |  |  |
| Alcoholic liver disease | Low/medium | 52 | 32 | 1.00 | 20 | 2.01 (0.88-4.58) | 0.10 |
|  | High | 27 | 18 | 1.00 | 9 | 1.19 (0.43-3.31) | 0.74 |
| Other chronic liver disease | Low/medium | 55 | 28 | 1.00 | 27 | 2.02 (0.97-4.20) | 0.06 |
|  | High | 32 | 18 | 1.00 | 14 | 1.67 (0.60-4.66) | 0.33 |
| Cholelithiasis, gallbladder disease | Low/medium | 9 | 4 | 1.00 | 5 | 1.15 (0.20-6.59) | 0.88 |
|  | High | 5 | 1 | 1.00 | 4 | 2.34 (0.19-29.54) | 0.51 |
| Urinary tract disease |  |  |  |  |  |  |  |
| Kidney failure | Low/medium | 132 | 48 | 1.00 | 84 | 1.81 (1.16-2.82) | 0.01 |
|  | High | 53 | 23 | 1.00 | 30 | 2.06 (1.04-4.08) | 0.04 |
| Abnormal clinical, lab findings | Low/medium | 80 | 44 | 1.00 | 36 | 1.11 (0.70-1.76) | 0.66 |
|  | High | 36 | 21 | 1.00 | 15 | 1.15 (0.53-2.47) | 0.72 |
| Transport injuries |  |  |  |  |  |  |  |
| Motor vehicle accidents | Low/medium | 104 | 72 | 1.00 | 32 | 1.56 (0.95-2.58) | 0.08 |
|  | High | 69 | 54 | 1.00 | 15 | 1.68 (0.86-3.29) | 0.13 |
| Unintentional injuries |  |  |  |  |  |  |  |
| Falls | Low/medium | 53 | 32 | 1.00 | 21 | 0.88 (0.49-1.60) | 0.68 |
|  | High | 27 | 18 | 1.00 | 9 | 1.39 (0.40-4.89) | 0.60 |
| Other non-transport accidents combined | Low/medium | 128 | 88 | 1.00 | 40 | 1.09 (0.69-1.74) | 0.70 |
|  | High | 57 | 42 | 1.00 | 15 | 1.41 (0.71-2.79) | 0.32 |
| Self-harm, interpersonal violence |  |  |  |  |  |  |  |
| Suicide | Low/medium | 77 | 59 | 1.00 | 18 | 0.96 (0.43-2.15) | 0.92 |
|  | High | 73 | 55 | 1.00 | 18 | 1.25 (0.67-2.32) | 0.48 |
| Homicide | Low/medium | 38 | 29 | 1.00 | 9 | 0.83 (0.34-1.99) | 0.67 |
|  | High | 17 | 16 | 1.00 | 1 | 0.26 (0.03-2.37) | 0.23 |
| All other diseases (residual) | Low/medium | 629 | 312 | 1.00 | 317 | 1.24 (1.03-1.51) | 0.03 |
|  | High | 267 | 144 | 1.00 | 123 | 1.44 (1.06-1.96) | 0.02 |
| All other causes/all unknown causes | Low/medium | 764 | 563 | 1.00 | 201 | 0.94 (0.77-1.16) | 0.59 |
|  | High | 344 | 247 | 1.00 | 97 | 1.14 (0.85-1.52) | 0.38 |

Multivariable adjustment for age, sex, education, race, income, alcohol, smoking status, BMI, physical activity, and survey year

Supplementary Table 7. Hazard ratios of all-cause mortality and cause-specific mortality among participants with hypertension compared to participants without hypertension stratified by income

|  |  | Total | No hypertension | | Hypertension | |  |
| --- | --- | --- | --- | --- | --- | --- | --- |
|  | Income | N  (deaths) | N  (deaths) | HR | N (deaths) | HR (95% CI) | P-value |
| All-cause mortality | Low/medium | 9326 | 4977 | 1.00 | 4349 | 1.23 (1.16-1.30) | <0.0001 |
|  | High | 1928 | 1142 | 1.00 | 786 | 1.32 (1.18-1.46) | <0.0001 |
| Infections |  |  |  |  |  |  |  |
| Septicemia | Low/medium | 122 | 55 | 1.00 | 67 | 1.76 (1.07-2.89) | 0.03 |
|  | High | 18 | 11 | 1.00 | 7 | 1.20 (0.41-3.51) | 0.74 |
| Viral hepatitis | Low/medium | 24 | 17 | 1.00 | 7 | 1.25 (0.38-4.12) | 0.71 |
|  | High | 4 | 1 | 1.00 | 3 | 3.75 (0.64-21.83) | 0.14 |
| Human immunodeficiency virus | Low/medium | 63 | 42 | 1.00 | 21 | 1.21 (0.51-2.90) | 0.67 |
|  | High | 9 | 5 | 1.00 | 4 | 5.73 (0.85-38.62) | 0.07 |
| Other infectious parasitic disease | Low/medium | 25 | 10 | 1.00 | 15 | 2.55 (0.96-6.75) | 0.06 |
|  | High | 6 | 3 | 1.00 | 3 | 2.64 (0.43-16.12) | 0.29 |
| Cancers |  |  |  |  |  |  |  |
| All cancers | Low/medium | 1765 | 1006 | 1.00 | 759 | 1.02 (0.91-1.14) | 0.71 |
|  | High | 545 | 341 | 1.00 | 204 | 1.12 (0.91-1.37) | 0.28 |
| Oral cavity, pharynx, lip | Low/medium | 19 | 13 | 1.00 | 6 | 0.71 (0.24-2.04) | 0.52 |
|  | High | 8 | 4 | 1.00 | 4 | 1.30 (0.31-5.36) | 0.72 |
| Esophagus | Low/medium | 37 | 19 | 1.00 | 18 | 1.67 (0.77-3.64) | 0.19 |
|  | High | 18 | 12 | 1.00 | 6 | 0.82 (0.29-2.27) | 0.70 |
| Stomach | Low/medium | 44 | 24 | 1.00 | 20 | 1.09 (0.55-2.17) | 0.81 |
|  | High | 9 | 8 | 1.00 | 1 | 0.46 (0.05-4.52) | 0.50 |
| Colon, rectum, anus | Low/medium | 173 | 101 | 1.00 | 72 | 1.03 (0.70-1.50) | 0.89 |
|  | High | 42 | 24 | 1.00 | 18 | 1.19 (0.58-2.44) | 0.63 |
| Liver and bile ducts | Low/medium | 49 | 30 | 1.00 | 19 | 0.79 (0.39-1.56) | 0.49 |
|  | High | 19 | 12 | 1.00 | 7 | 0.97 (0.31-3.01) | 0.96 |
| Pancreas | Low/medium | 107 | 55 | 1.00 | 52 | 1.04 (0.66-1.64) | 0.85 |
|  | High | 43 | 26 | 1.00 | 17 | 1.04 (0.50-2.18) | 0.91 |
| Larynx | Low/medium | 17 | 12 | 1.00 | 5 | 0.60 (0.18-2.06) | 0.42 |
|  | High | 3 | 1 | 1.00 | 2 | 3.01 (0.32-28.50) | 0.34 |
| Lung, trachea, bronchus | Low/medium | 564 | 319 | 1.00 | 245 | 1.22 (0.98-1.53) | 0.08 |
|  | High | 169 | 105 | 1.00 | 64 | 1.15 (0.83-1.60) | 0.40 |
| Malignant melanoma | Low/medium | 17 | 12 | 1.00 | 5 | 0.41 (0.13-1.27) | 0.12 |
|  | High | 8 | 5 | 1.00 | 3 | 1.72 (0.17-17.06) | 0.64 |
| Breast (females) | Low/medium | 106 | 63 | 1.00 | 43 | 0.79 (0.49-1.26) | 0.32 |
|  | High | 22 | 16 | 1.00 | 6 | 1.10 (0.35-3.45) | 0.87 |
| Cervix uteri (females) | Low/medium | 15 | 8 | 1.00 | 7 | 0.96 (0.31-2.95) | 0.94 |
|  | High | 4 | 3 | 1.00 | 1 | 0.59 (0.06-5.89) | 0.65 |
| Uterus (females) | Low/medium | 25 | 17 | 1.00 | 8 | 0.42 (0.15-1.15) | 0.09 |
|  | High | 7 | 6 | 1.00 | 1 | 0.18 (0.02-1.87) | 0.15 |
| Ovaries (females) | Low/medium | 48 | 25 | 1.00 | 23 | 0.97 (0.51-1.85) | 0.92 |
|  | High | 14 | 11 | 1.00 | 3 | - | - |
| Prostate (males) | Low/medium | 66 | 37 | 1.00 | 29 | 0.84 (0.50-1.41) | 0.52 |
|  | High | 20 | 12 | 1.00 | 8 | - | - |
| Kidney and renal pelvis | Low/medium | 46 | 24 | 1.00 | 22 | 1.44 (0.65-3.17) | 0.37 |
|  | High | 12 | 8 | 1.00 | 4 | 1.72 (0.44-6.68) | 0.43 |
| Bladder | Low/medium | 38 | 29 | 1.00 | 9 | 0.36 (0.16-0.84) | 0.02 |
|  | High | 10 | 6 | 1.00 | 4 | 1.59 (0.41-6.09) | 0.50 |
| Brain, nervous system | Low/medium | 24 | 11 | 1.00 | 13 | 1.92 (0.70-5.32) | 0.21 |
|  | High | 18 | 12 | 1.00 | 6 | 0.90 (0.30-2.72) | 0.86 |
| Hodgkin's disease | Low/medium | 7 | 6 | 1.00 | 1 | 0.34 (0.04-3.11) | 0.34 |
|  | High | 1 | 0 | 1.00 | 1 | - | - |
| Non-Hodgkin's lymphoma | Low/medium | 50 | 25 | 1.00 | 25 | 1.18 (0.59-2.37) | 0.63 |
|  | High | 17 | 9 | 1.00 | 8 | 2.07 (0.71-6.03) | 0.18 |
| Leukemia | Low/medium | 69 | 45 | 1.00 | 24 | 0.65 (0.37-1.15) | 0.14 |
|  | High | 19 | 12 | 1.00 | 7 | 1.22 (0.45-3.27) | 0.70 |
| Multiple myeloma | Low/medium | 25 | 10 | 1.00 | 15 | 1.50 (0.62-3.63) | 0.37 |
|  | High | 12 | 10 | 1.00 | 2 | 0.23 (0.06-0.94) | 0.04 |
| All other and unspecified neoplasms | Low/medium | 219 | 121 | 1.00 | 98 | 1.09 (0.76-1.55) | 0.64 |
|  | High | 70 | 39 | 1.00 | 31 | 1.44 (0.84-2.47) | 0.18 |
| In situ and benign neoplasms | Low/medium | 44 | 27 | 1.00 | 17 | 0.53 (0.26-1.09) | 0.08 |
|  | High | 7 | 3 | 1.00 | 4 | 2.07 (0.25-16.85) | 0.49 |
| Endocrine, nutritional, metabolic diseases |  |  |  |  |  |  |  |
| Anaemia | Low/medium | 15 | 8 | 1.00 | 7 | 1.63 (0.40-6.65) | 0.49 |
|  | High | 2 | 2 | 1.00 | 0 | - | - |
| Diabetes mellitus | Low/medium | 280 | 94 | 1.00 | 186 | 2.05 (1.48-2.83) | <0.0001 |
|  | High | 39 | 19 | 1.00 | 20 | 1.70 (0.73-3.95) | 0.21 |
| Malnutrition | Low/medium | 12 | 8 | 1.00 | 4 | - | - |
|  | High | 2 | 1 | 1.00 | 1 | - | - |
| Nervous system |  |  |  |  |  |  |  |
| Parkinson's disease | Low/medium | 48 | 29 | 1.00 | 19 | 0.85 (0.44-1.64) | 0.63 |
|  | High | 13 | 5 | 1.00 | 8 | 2.45 (0.71-8.42) | 0.15 |
| Alzheimer's disease | Low/medium | 178 | 106 | 1.00 | 72 | 0.74 (0.52-1.05) | 0.09 |
|  | High | 29 | 22 | 1.00 | 7 | 0.30 (0.11-0.84) | 0.02 |
| Circulatory disease |  |  |  |  |  |  |  |
| All circulatory diseases | Low/medium | 3246 | 1474 | 1.00 | 1772 | 1.44 (1.32-1.58) | <0.0001 |
|  | High | 575 | 282 | 1.00 | 293 | 1.64 (1.34-2.01) | <0.0001 |
| Hypertensive heart disease | Low/medium | 112 | 36 | 1.00 | 76 | 3.31 (1.95-5.62) | <0.0001 |
|  | High | 22 | 10 | 1.00 | 12 | 2.92 (0.97-8.77) | 0.06 |
| Hypertensive heart and renal disease | Low/medium | 16 | 4 | 1.00 | 12 | 2.02 (0.58-7.10) | 0.27 |
|  | High | 1 | 0 | 1.00 | 1 | - | - |
| Ischaemic heart disease | Low/medium | 1747 | 832 | 1.00 | 915 | 1.32 (1.19-1.47) | <0.0001 |
|  | High | 303 | 159 | 1.00 | 144 | 1.40 (1.08-1.82) | 0.01 |
| Acute myocardial infarction | Low/medium | 654 | 296 | 1.00 | 358 | 1.43 (1.19-1.71) | <0.0001 |
|  | High | 123 | 63 | 1.00 | 60 | 1.72 (1.13-2.62) | 0.01 |
| Other acute ischaemic heart disease | Low/medium | 10 | 2 | 1.00 | 8 | 7.02 (1.03-47.75) | 0.05 |
|  | High | 5 | 3 | 1.00 | 2 | 0.88 (0.17-4.56) | 0.88 |
| Atherosclerotic cardiovascular disease | Low/medium | 258 | 148 | 1.00 | 110 | 0.95 (0.69-1.30) | 0.73 |
|  | High | 43 | 27 | 1.00 | 16 | 1.01 (0.47-2.16) | 0.98 |
| Other chronic ischaemic heart disease | Low/medium | 825 | 386 | 1.00 | 439 | 1.35 (1.15-1.58) | <0.0001 |
|  | High | 132 | 66 | 1.00 | 66 | 1.27 (0.84-1.91) | 0.26 |
| Heart failure | Low/medium | 213 | 103 | 1.00 | 110 | 1.09 (0.78-1.52) | 0.61 |
|  | High | 22 | 7 | 1.00 | 15 | 2.21 (0.70-7.02) | 0.18 |
| All other forms of heart disease | Low/medium | 400 | 180 | 1.00 | 220 | 1.53 (1.20-1.94) | 0.001 |
|  | High | 80 | 46 | 1.00 | 34 | 1.43 (0.82-2.49) | 0.21 |
| Primary hypertension, renal disease | Low/medium | 103 | 34 | 1.00 | 69 | 2.61 (1.50-4.51) | 0.001 |
|  | High | 12 | 2 | 1.00 | 10 | 13.29 (2.02-87.43) | 0.007 |
| Cerebrovascular disease | Low/medium | 513 | 222 | 1.00 | 291 | 1.56 (1.28-1.90) | <0.0001 |
|  | High | 105 | 44 | 1.00 | 61 | 1.89 (1.18-3.02) | 0.008 |
| Atherosclerosis | Low/medium | 25 | 10 | 1.00 | 15 | 2.69 (1.09-6.66) | 0.03 |
|  | High | 5 | 4 | 1.00 | 1 | - | - |
| Other diseases of circulatory system | Low/medium | 117 | 53 | 1.00 | 64 | 1.48 (0.89-2.47) | 0.13 |
|  | High | 25 | 10 | 1.00 | 15 | 2.63 (0.92-7.56) | 0.07 |
| Aortic aneurysm and dissection | Low/medium | 56 | 25 | 1.00 | 31 | 1.25 (0.64-2.42) | 0.52 |
|  | High | 18 | 7 | 1.00 | 11 | 2.63 (0.82-8.43) | 0.10 |
| Other diseases of arteries or capillaries | Low/medium | 44 | 21 | 1.00 | 23 | 1.50 (0.61-3.70) | 0.38 |
|  | High | 4 | 1 | 1.00 | 3 | - | - |
| Other disorders of circulatory system | Low/medium | 17 | 7 | 1.00 | 10 | 3.38 (0.79-14.49) | 0.10 |
|  | High | 3 | 2 | 1.00 | 1 | - | - |
| Respiratory diseases |  |  |  |  |  |  |  |
| Pneumonia | Low/medium | 207 | 118 | 1.00 | 89 | 0.92 (0.67-1.26) | 0.59 |
|  | High | 32 | 19 | 1.00 | 13 | 1.17 (0.54-2.53) | 0.68 |
| Emphysema | Low/medium | 52 | 27 | 1.00 | 25 | 1.60 (0.84-3.06) | 0.15 |
|  | High | 10 | 9 | 1.00 | 1 | 0.23 (0.03-1.92) | 0.18 |
| Other chronic lower respiratory disease | Low/medium | 433 | 226 | 1.00 | 207 | 1.38 (1.10-1.73) | 0.006 |
|  | High | 59 | 32 | 1.00 | 27 | 1.33 (0.68-2.61) | 0.41 |
| Pneumonitis from solids, liquids | Low/medium | 64 | 40 | 1.00 | 24 | 0.70 (0.36-1.36) | 0.29 |
|  | High | 10 | 7 | 1.00 | 3 | 0.86 (0.18-4.15) | 0.85 |
| Other respiratory system diseases | Low/medium | 125 | 67 | 1.00 | 58 | 1.03 (0.65-1.62) | 0.91 |
|  | High | 16 | 7 | 1.00 | 9 | 2.20 (0.71-6.79) | 0.17 |
| Digestive diseases |  |  |  |  |  |  |  |
| Alcoholic liver disease | Low/medium | 67 | 43 | 1.00 | 24 | 1.86 (0.93-3.70) | 0.08 |
|  | High | 13 | 8 | 1.00 | 5 | 1.14 (0.24-5.32) | 0.87 |
| Other chronic liver disease | Low/medium | 60 | 32 | 1.00 | 28 | 2.06 (1.04-4.07) | 0.04 |
|  | High | 27 | 14 | 1.00 | 13 | 1.54 (0.57-4.20) | 0.39 |
| Cholelithiasis, gallbladder disease | Low/medium | 11 | 4 | 1.00 | 7 | 1.87 (0.38-9.16) | 0.44 |
|  | High | 3 | 1 | 1.00 | 2 | 0.94 (0.09-10.16) | 0.96 |
| Urinary tract disease |  |  |  |  |  |  |  |
| Kidney failure | Low/medium | 162 | 61 | 1.00 | 101 | 1.82 (1.22-2.73) | 0.004 |
|  | High | 24 | 10 | 1.00 | 14 | 2.15 (0.86-5.37) | 0.10 |
| Abnormal clinical, lab findings | Low/medium | 95 | 51 | 1.00 | 44 | 1.10 (0.72-1.69) | 0.65 |
|  | High | 21 | 14 | 1.00 | 7 | 1.13 (0.40-3.19) | 0.82 |
| Transport injuries |  |  |  |  |  |  |  |
| Motor vehicle accidents | Low/medium | 147 | 105 | 1.00 | 42 | 1.67 (1.10-2.53) | 0.02 |
|  | High | 26 | 21 | 1.00 | 5 | 1.30 (0.40-4.25) | 0.66 |
| Unintentional injuries |  |  |  |  |  |  |  |
| Falls | Low/medium | 60 | 36 | 1.00 | 24 | 0.88 (0.51-1.50) | 0.64 |
|  | High | 21 | 15 | 1.00 | 6 | 1.26 (0.31-5.19) | 0.74 |
| Other non-transport accidents combined | Low/medium | 149 | 105 | 1.00 | 44 | 1.03 (0.67-1.56) | 0.91 |
|  | High | 37 | 25 | 1.00 | 12 | 1.79 (0.82-3.90) | 0.14 |
| Self-harm, interpersonal violence |  |  |  |  |  |  |  |
| Suicide | Low/medium | 102 | 74 | 1.00 | 28 | 1.34 (0.71-2.53) | 0.37 |
|  | High | 49 | 41 | 1.00 | 8 | 0.63 (0.25-1.58) | 0.33 |
| Homicide | Low/medium | 49 | 39 | 1.00 | 10 | 0.74 (0.33-1.67) | 0.47 |
|  | High | 7 | 7 | 1.00 | 0 | - | - |
| All other diseases (residual) | Low/medium | 791 | 401 | 1.00 | 390 | 1.23 (1.03-1.48) | 0.02 |
|  | High | 119 | 63 | 1.00 | 56 | 1.64 (1.10-2.47) | 0.02 |
| All other causes/all unknown causes | Low/medium | 930 | 672 | 1.00 | 258 | 0.99 (0.82-1.19) | 0.91 |
|  | High | 205 | 154 | 1.00 | 51 | 1.05 (0.73-1.53) | 0.79 |

Multivariable adjustment for age, sex, education, race, income, alcohol, smoking status, BMI, physical activity, and survey year

Supplementary Table 8. Hazard ratios of all-cause mortality and cause-specific mortality among participants with hypertension vs. no hypertension stratified by BMI

|  |  | Total | No hypertension | | Hypertension | |  |
| --- | --- | --- | --- | --- | --- | --- | --- |
|  | BMI | N  (deaths) | N  (deaths) | HR | N (deaths) | HR (95% CI) | P-value |
| All-cause mortality | <25 | 5131 | 3139 | 1.00 | 1992 | 1.23 (1.14-1.33) | <0.0001 |
|  | 25-<30 | 3556 | 1875 | 1.00 | 1681 | 1.27 (1.16-1.38) | <0.0001 |
|  | ≥30 | 2139 | 853 | 1.00 | 1286 | 1.34 (1.21-1.50) | <0.0001 |
| Infections |  |  |  |  |  |  |  |
| Septicemia | <25 | 59 | 32 | 1.00 | 27 | 1.94 (1.04-3.64) | 0.04 |
|  | 25-<30 | 40 | 15 | 1.00 | 25 | 2.20 (0.98-4.94) | 0.06 |
|  | ≥30 | 33 | 13 | 1.00 | 20 | 1.58 (0.62-4.04) | 0.34 |
| Viral hepatitis | <25 | 13 | 10 | 1.00 | 3 | 1.10 (0.19-6.37) | 0.92 |
|  | 25-<30 | 9 | 4 | 1.00 | 5 | 2.20 (0.55-8.80) | 0.27 |
|  | ≥30 | 4 | 4 | 1.00 | 0 | - | - |
| Human immunodeficiency virus | <25 | 42 | 32 | 1.00 | 10 | 1.31 (0.53-3.28) | 0.56 |
|  | 25-<30 | 20 | 10 | 1.00 | 10 | 2.90 (0.41-20.37) | 0.28 |
|  | ≥30 | 10 | 5 | 1.00 | 5 | 0.91 (0.16-5.20) | 0.91 |
| Other infectious parasitic disease | <25 | 15 | 10 | 1.00 | 5 | - | - |
|  | 25-<30 | 10 | 1 | 1.00 | 9 | 12.80 (1.10-149.44) | 0.04 |
|  | ≥30 | 4 | 1 | 1.00 | 3 | - | - |
| Cancers |  |  |  |  |  |  |  |
| All cancers | <25 | 1006 | 682 | 1.00 | 324 | 0.99 (0.84-1.17) | 0.95 |
|  | 25-<30 | 814 | 438 | 1.00 | 376 | 1.13 (0.96-1.33) | 0.15 |
|  | ≥30 | 424 | 184 | 1.00 | 234 | 1.00 (0.79-1.27) | 1.00 |
| Oral cavity, pharynx, lip | <25 | 16 | 13 | 1.00 | 3 | 0.38 (0.10-1.46) | 0.16 |
|  | 25-<30 | 6 | 3 | 1.00 | 3 | 0.74 (0.16-3.58) | 0.71 |
|  | ≥30 | 5 | 1 | 1.00 | 4 | - | - |
| Esophagus | <25 | 22 | 15 | 1.00 | 7 | 1.28 (0.52-3.11) | 0.59 |
|  | 25-<30 | 21 | 12 | 1.00 | 9 | 1.33 (0.41-4.26) | 0.63 |
|  | ≥30 | 12 | 4 | 1.00 | 8 | 1.52 (0.41-5.62) | 0.53 |
| Stomach | <25 | 23 | 18 | 1.00 | 5 | 0.77 (0.27-2.18) | 0.63 |
|  | 25-<30 | 19 | 9 | 1.00 | 10 | 1.64 (0.56-4.83) | 0.37 |
|  | ≥30 | 9 | 4 | 1.00 | 5 | 0.48 (0.10-2.32) | 0.36 |
| Colon, rectum, anus | <25 | 82 | 56 | 1.00 | 26 | 0.85 (0.48-1.48) | 0.56 |
|  | 25-<30 | 79 | 44 | 1.00 | 35 | 1.24 (0.76-2.04) | 0.39 |
|  | ≥30 | 43 | 21 | 1.00 | 22 | 0.82 (0.39-1.69) | 0.59 |
| Liver and bile ducts | <25 | 19 | 15 | 1.00 | 4 | 0.37 (0.10-1.32) | 0.12 |
|  | 25-<30 | 31 | 20 | 1.00 | 11 | 0.75 (0.28-1.97) | 0.55 |
|  | ≥30 | 17 | 6 | 1.00 | 11 | 2.08 (0.59-7.31) | 0.25 |
| Pancreas | <25 | 63 | 38 | 1.00 | 25 | 1.26 (0.66-2.43) | 0.49 |
|  | 25-<30 | 47 | 26 | 1.00 | 21 | 0.69 (0.35-1.36) | 0.28 |
|  | ≥30 | 36 | 16 | 1.00 | 20 | 1.02 (0.47-2.20) | 0.96 |
| Larynx | <25 | 14 | 9 | 1.00 | 5 | 0.90 (0.30-2.68) | 0.85 |
|  | 25-<30 | 3 | 1 | 1.00 | 2 | 1.91 (0.09-42.25) | 0.68 |
|  | ≥30 | 2 | 2 | 1.00 | 0 | - | - |
| Lung, trachea, bronchus | <25 | 377 | 249 | 1.00 | 128 | 1.18 (0.90-1.55) | 0.24 |
|  | 25-<30 | 240 | 116 | 1.00 | 124 | 1.47 (1.07-2.02) | 0.02 |
|  | ≥30 | 93 | 41 | 1.00 | 52 | 0.96 (0.59-1.54) | 0.85 |
| Malignant melanoma | <25 | 7 | 4 | 1.00 | 3 | 1.22 (0.25-5.86) | 0.80 |
|  | 25-<30 | 16 | 12 | 1.00 | 4 | 0.61 (0.14-2.64) | 0.51 |
|  | ≥30 | 2 | 1 | 1.00 | 1 | - | - |
| Breast (females) | <25 | 57 | 41 | 1.00 | 16 | 0.79 (0.42-1.47) | 0.46 |
|  | 25-<30 | 27 | 14 | 1.00 | 13 | - | - |
|  | ≥30 | 34 | 17 | 1.00 | 17 | 0.58 (0.24-1.43) | 0.24 |
| Cervix uteri (females) | <25 | 8 | 7 | 1.00 | 1 | 0.26 (0.03-2.02) | 0.20 |
|  | 25-<30 | 6 | 3 | 1.00 | 3 | 1.44 (0.23-8.89) | 0.69 |
|  | ≥30 | 4 | 1 | 1.00 | 3 | 1.12 (0.11-11.15) | 0.92 |
| Uterus (females) | <25 | 7 | 6 | 1.00 | 1 | 0.25 (0.03-2.26) | 0.22 |
|  | 25-<30 | 9 | 7 | 1.00 | 2 | 0.10 (0.02-0.63) | 0.01 |
|  | ≥30 | 15 | 10 | 1.00 | 5 | 0.59 (0.15-2.40) | 0.46 |
| Ovaries (females) | <25 | 27 | 19 | 1.00 | 8 | 0.98 (0.39-2.47) | 0.96 |
|  | 25-<30 | 22 | 10 | 1.00 | 12 | 0.99 (0.40-2.45) | 0.99 |
|  | ≥30 | 12 | 7 | 1.00 | 5 | 0.62 (0.17-2.27) | 0.47 |
| Prostate (males) | <25 | 40 | 25 | 1.00 | 15 | 0.84 (0.43-1.66) | 0.62 |
|  | 25-<30 | 35 | 21 | 1.00 | 14 | 0.78 (0.33-1.81) | 0.56 |
|  | ≥30 | 10 | 2 | 1.00 | 8 | 2.30 (0.63-8.39) | 0.21 |
| Kidney and renal pelvis | <25 | 18 | 13 | 1.00 | 5 | 0.73 (0.26-2.05) | 0.54 |
|  | 25-<30 | 21 | 11 | 1.00 | 10 | 1.60 (0.50-5.11) | 0.42 |
|  | ≥30 | 19 | 8 | 1.00 | 11 | 1.84 (0.55-6.16) | 0.32 |
| Bladder | <25 | 22 | 18 | 1.00 | 4 | 0.59 (0.17-2.02) | 0.40 |
|  | 25-<30 | 15 | 10 | 1.00 | 5 | 0.51 (0.15-1.72) | 0.28 |
|  | ≥30 | 10 | 6 | 1.00 | 4 | 0.45 (0.11-1.90) | 0.28 |
| Brain, nervous system | <25 | 15 | 11 | 1.00 | 4 | 0.99 (0.23-4.27) | 0.99 |
|  | 25-<30 | 17 | 10 | 1.00 | 7 | 0.86 (0.31-2.40) | 0.77 |
|  | ≥30 | 8 | 2 | 1.00 | 6 | 4.44 (0.75-26.08) | 0.10 |
| Hodgkin's disease | <25 | 1 | 1 | 1.00 | 0 | - | - |
|  | 25-<30 | 7 | 5 | 1.00 | 2 | 0.90 (0.18-4.50) | 0.89 |
|  | ≥30 | 0 | 0 | 1.00 | 0 | - | - |
| Non-Hodgkin's lymphoma | <25 | 29 | 15 | 1.00 | 14 | 1.93 (0.80-4.62) | 0.14 |
|  | 25-<30 | 26 | 14 | 1.00 | 12 | 1.22 (0.48-3.07) | 0.68 |
|  | ≥30 | 11 | 5 | 1.00 | 6 | 0.75 (0.18-3.04) | 0.68 |
| Leukemia | <25 | 29 | 22 | 1.00 | 7 | 0.70 (0.27-1.78) | 0.45 |
|  | 25-<30 | 37 | 21 | 1.00 | 16 | 0.90 (0.41-1.95) | 0.78 |
|  | ≥30 | 16 | 10 | 1.00 | 6 | - | - |
| Multiple myeloma | <25 | 18 | 12 | 1.00 | 6 | 0.65 (0.22-1.91) | 0.43 |
|  | 25-<30 | 11 | 7 | 1.00 | 4 | 0.37 (0.11-1.28) | 0.12 |
|  | ≥30 | 8 | 1 | 1.00 | 7 | 4.53 (0.51-40.21) | 0.18 |
| All other and unspecified neoplasms | <25 | 112 | 75 | 1.00 | 37 | 1.06 (0.65-1.71) | 0.82 |
|  | 25-<30 | 119 | 62 | 1.00 | 57 | 1.29 (0.82-2.02) | 0.27 |
|  | ≥30 | 52 | 19 | 1.00 | 33 | 1.50 (0.75-2.99) | 0.25 |
| In situ and benign neoplasms | <25 | 23 | 17 | 1.00 | 6 | 0.38 (0.12-1.20) | 0.10 |
|  | 25-<30 | 20 | 10 | 1.00 | 10 | 1.12 (0.45-2.77) | 0.81 |
|  | ≥30 | 6 | 2 | 1.00 | 4 | - | - |
| Endocrine, nutritional, metabolic diseases |  |  |  |  |  |  |  |
| Anaemia | <25 | 6 | 3 | 1.00 | 3 | 1.58 (0.24-10.27) | 0.63 |
|  | 25-<30 | 8 | 5 | 1.00 | 3 | - | - |
|  | ≥30 | 3 | 2 | 1.00 | 1 | 0.08 (0.01-0.92) | 0.04 |
| Diabetes mellitus | <25 | 95 | 49 | 1.00 | 46 | 1.29 (0.75-2.21) | 0.36 |
|  | 25-<30 | 105 | 37 | 1.00 | 68 | 2.31 (1.42-3.76) | 0.001 |
|  | ≥30 | 102 | 19 | 1.00 | 83 | 2.85 (1.53-5.30) | 0.001 |
| Malnutrition | <25 | 10 | 6 | 1.00 | 4 | - | - |
|  | 25-<30 | 3 | 2 | 1.00 | 1 | 0.32 (0.02-4.91) | 0.41 |
|  | ≥30 | 1 | 1 | 1.00 | 0 | - | - |
| Nervous system |  |  |  |  |  |  |  |
| Parkinson's disease | <25 | 36 | 22 | 1.00 | 14 | 1.08 (0.51-2.32) | 0.84 |
|  | 25-<30 | 16 | 10 | 1.00 | 6 | 0.87 (0.29-2.63) | 0.81 |
|  | ≥30 | 6 | 1 | 1.00 | 5 | 6.42 (0.62-66.41) | 0.12 |
| Alzheimer's disease | <25 | 132 | 83 | 1.00 | 49 | 0.83 (0.55-1.24) | 0.36 |
|  | 25-<30 | 44 | 28 | 1.00 | 16 | 0.39 (0.21-0.73) | 0.003 |
|  | ≥30 | 21 | 11 | 1.00 | 10 | 0.50 (0.17-1.46) | 0.20 |
| Circulatory disease |  |  |  |  |  |  |  |
| All circulatory diseases | <25 | 1752 | 898 | 1.00 | 854 | 1.49 (1.32-1.68) | <0.0001 |
|  | 25-<30 | 1180 | 537 | 1.00 | 643 | 1.50 (1.30-1.73) | <0.0001 |
|  | ≥30 | 766 | 257 | 1.00 | 509 | 1.53 (1.25-1.86) | <0.0001 |
| Hypertensive heart disease | <25 | 46 | 17 | 1.00 | 29 | 3.33 (1.63-6.84) | 0.001 |
|  | 25-<30 | 42 | 17 | 1.00 | 25 | 2.37 (0.98-5.76) | 0.06 |
|  | ≥30 | 42 | 10 | 1.00 | 32 | 4.49 (1.88-10.69) | 0.001 |
| Hypertensive heart and renal disease | <25 | 6 | 2 | 1.00 | 4 | - | - |
|  | 25-<30 | 2 | 0 | 1.00 | 2 | - | - |
|  | ≥30 | 7 | 1 | 1.00 | 6 | 2.51 (0.22-28.85) | 0.46 |
| Ischaemic heart disease | <25 | 920 | 496 | 1.00 | 424 | 1.35 (1.15-1.58) | <0.0001 |
|  | 25-<30 | 647 | 313 | 1.00 | 334 | 1.45 (1.20-1.76) | <0.0001 |
|  | ≥30 | 419 | 144 | 1.00 | 275 | 1.30 (1.01-1.68) | 0.04 |
| Acute myocardial infarction | <25 | 334 | 177 | 1.00 | 157 | 1.45 (1.11-1.91) | 0.008 |
|  | 25-<30 | 255 | 111 | 1.00 | 144 | 1.78 (1.25-2.54) | 0.002 |
|  | ≥30 | 163 | 54 | 1.00 | 109 | 1.39 (0.95-2.03) | 0.09 |
| Other acute ischaemic heart disease | <25 | 6 | 3 | 1.00 | 3 | 1.06 (0.16-7.16) | 0.95 |
|  | 25-<30 | 7 | 2 | 1.00 | 5 | - | - |
|  | ≥30 | 2 | 0 | 1.00 | 2 | - | - |
| Atherosclerotic cardiovascular disease | <25 | 136 | 83 | 1.00 | 53 | 1.14 (0.76-1.71) | 0.53 |
|  | 25-<30 | 85 | 59 | 1.00 | 26 | 0.53 (0.29-0.97) | 0.04 |
|  | ≥30 | 74 | 29 | 1.00 | 45 | 1.37 (0.73-2.59) | 0.33 |
| Other chronic ischaemic heart disease | <25 | 444 | 233 | 1.00 | 211 | 1.35 (1.10-1.65) | 0.004 |
|  | 25-<30 | 300 | 141 | 1.00 | 159 | 1.50 (1.12-2.00) | 0.006 |
|  | ≥30 | 180 | 61 | 1.00 | 119 | 1.20 (0.80-1.78) | 0.38 |
| Heart failure | <25 | 117 | 61 | 1.00 | 56 | 1.09 (0.74-1.62) | 0.65 |
|  | 25-<30 | 70 | 29 | 1.00 | 41 | 1.33 (0.77-2.30) | 0.30 |
|  | ≥30 | 38 | 15 | 1.00 | 23 | 1.31 (0.57-2.98) | 0.53 |
| All other forms of heart disease | <25 | 205 | 109 | 1.00 | 96 | 1.52 (1.11-2.08) | 0.01 |
|  | 25-<30 | 148 | 68 | 1.00 | 80 | 1.24 (0.82-1.89) | 0.31 |
|  | ≥30 | 113 | 43 | 1.00 | 70 | 1.73 (1.05-2.83) | 0.03 |
| Primary hypertension, renal disease | <25 | 55 | 18 | 1.00 | 37 | 3.49 (1.62-7.51) | 0.002 |
|  | 25-<30 | 29 | 9 | 1.00 | 20 | 2.97 (1.19-7.44) | 0.02 |
|  | ≥30 | 29 | 9 | 1.00 | 20 | 2.17 (0.74-6.38) | 0.16 |
| Cerebrovascular disease | <25 | 316 | 151 | 1.00 | 165 | 1.63 (1.26-2.11) | <0.0001 |
|  | 25-<30 | 194 | 82 | 1.00 | 112 | 1.57 (1.12-2.19) | 0.009 |
|  | ≥30 | 91 | 27 | 1.00 | 64 | 1.77 (1.04-3.00) | 0.04 |
| Atherosclerosis | <25 | 19 | 9 | 1.00 | 10 | 1.87 (0.64-5.45) | 0.25 |
|  | 25-<30 | 6 | 4 | 1.00 | 2 | - | - |
|  | ≥30 | 3 | 0 | 1.00 | 3 | - | - |
| Other diseases of circulatory system | <25 | 68 | 35 | 1.00 | 33 | 1.79 (0.94-3.39) | 0.07 |
|  | 25-<30 | 42 | 15 | 1.00 | 27 | 2.33 (0.89-6.10) | 0.08 |
|  | ≥30 | 24 | 8 | 1.00 | 16 | 1.54 (0.59-4.05) | 0.38 |
| Aortic aneurysm and dissection | <25 | 35 | 18 | 1.00 | 17 | 1.78 (0.78-4.07) | 0.17 |
|  | 25-<30 | 23 | 8 | 1.00 | 15 | 1.44 (0.49-4.26) | 0.51 |
|  | ≥30 | 13 | 4 | 1.00 | 9 | 2.40 (0.77-7.50) | 0.13 |
| Other diseases of arteries or capillaries | <25 | 21 | 11 | 1.00 | 10 | 1.37 (0.47-3.98) | 0.57 |
|  | 25-<30 | 13 | 5 | 1.00 | 8 | 5.32 (0.78-36.18) | 0.09 |
|  | ≥30 | 9 | 3 | 1.00 | 6 | 1.03 (0.21-5.09) | 0.97 |
| Other disorders of circulatory system | <25 | 12 | 6 | 1.00 | 6 | 3.19 (0.69-14.66) | 0.14 |
|  | 25-<30 | 6 | 2 | 1.00 | 4 | 2.76 (0.21-35.48) | 0.44 |
|  | ≥30 | 2 | 1 | 1.00 | 1 | 0.38 (0.01-17.01) | 0.62 |
| Respiratory diseases |  |  |  |  |  |  |  |
| Pneumonia | <25 | 128 | 85 | 1.00 | 43 | 0.78 (0.51-1.18) | 0.24 |
|  | 25-<30 | 61 | 26 | 1.00 | 35 | 1.34 (0.73-2.46) | 0.35 |
|  | ≥30 | 31 | 14 | 1.00 | 17 | 0.95 (0.47-1.93) | 0.89 |
| Emphysema | <25 | 39 | 23 | 1.00 | 16 | 1.57 (0.78-3.16) | 0.21 |
|  | 25-<30 | 19 | 10 | 1.00 | 9 | - | - |
|  | ≥30 | 3 | 2 | 1.00 | 1 | - | - |
| Other chronic lower respiratory disease | <25 | 296 | 176 | 1.00 | 120 | 1.34 (1.00-1.80) | 0.048 |
|  | 25-<30 | 114 | 53 | 1.00 | 61 | 1.30 (0.87-1.96) | 0.20 |
|  | ≥30 | 66 | 22 | 1.00 | 44 | 1.73 (0.91-3.30) | 0.10 |
| Pneumonitis from solids, liquids | <25 | 38 | 22 | 1.00 | 16 | 1.19 (0.52-2.72) | 0.68 |
|  | 25-<30 | 21 | 15 | 1.00 | 6 | 0.49 (0.17-1.42) | 0.19 |
|  | ≥30 | 10 | 5 | 1.00 | 5 | 0.37 (0.08-1.79) | 0.22 |
| Other respiratory system diseases | <25 | 65 | 39 | 1.00 | 26 | 1.45 (0.78-2.70) | 0.24 |
|  | 25-<30 | 45 | 25 | 1.00 | 20 | 0.79 (0.39-1.60) | 0.51 |
|  | ≥30 | 28 | 9 | 1.00 | 19 | 1.24 (0.47-3.28) | 0.66 |
| Digestive diseases |  |  |  |  |  |  |  |
| Alcoholic liver disease | <25 | 33 | 22 | 1.00 | 11 | 1.57 (0.78-3.16) | 0.21 |
|  | 25-<30 | 29 | 18 | 1.00 | 11 | 2.54 (0.96-6.73) | 0.06 |
|  | ≥30 | 16 | 9 | 1.00 | 7 | 1.22 (0.37-4.02) | 0.75 |
| Other chronic liver disease | <25 | 36 | 26 | 1.00 | 10 | 1.41 (0.51-3.93) | 0.51 |
|  | 25-<30 | 26 | 11 | 1.00 | 15 | 2.42 (1.01-5.80) | 0.047 |
|  | ≥30 | 21 | 6 | 1.00 | 15 | 3.16 (0.91-10.97) | 0.07 |
| Cholelithiasis, gallbladder disease | <25 | 7 | 4 | 1.00 | 3 | 1.38 (0.49-3.90) | 0.54 |
|  | 25-<30 | 3 | 1 | 1.00 | 2 | 0.44 (0.06-3.22) | 0.42 |
|  | ≥30 | 3 | 0 | 1.00 | 3 | - | - |
| Urinary tract disease |  |  |  |  |  |  |  |
| Kidney failure | <25 | 84 | 36 | 1.00 | 48 | 1.58 (0.35-7.24) | 0.55 |
|  | 25-<30 | 53 | 23 | 1.00 | 30 | 1.29 (0.64-2.60) | 0.47 |
|  | ≥30 | 45 | 8 | 1.00 | 37 | 4.45 (1.66-11.91) | 0.003 |
| Abnormal clinical, lab findings | <25 | 52 | 29 | 1.00 | 23 | 1.99 (1.19-3.33) | 0.009 |
|  | 25-<30 | 37 | 26 | 1.00 | 11 | 0.60 (0.27-1.35) | 0.22 |
|  | ≥30 | 22 | 8 | 1.00 | 14 | 1.66 (0.68-4.08) | 0.27 |
| Transport injuries |  |  |  |  |  |  |  |
| Motor vehicle accidents | <25 | 61 | 52 | 1.00 | 9 | 1.49 (0.81-2.73) | 0.20 |
|  | 25-<30 | 63 | 47 | 1.00 | 16 | 1.11 (0.65-1.90) | 0.71 |
|  | ≥30 | 43 | 22 | 1.00 | 21 | 2.48 (1.14-5.40) | 0.02 |
| Unintentional injuries |  |  |  |  |  |  |  |
| Falls | <25 | 43 | 28 | 1.00 | 15 | 1.75 (0.67-4.59) | 0.25 |
|  | 25-<30 | 22 | 16 | 1.00 | 6 | 1.50 (0.31-7.29) | 0.62 |
|  | ≥30 | 12 | 4 | 1.00 | 8 | 2.53 (0.63-10.09) | 0.19 |
| Other non-transport accidents combined | <25 | 72 | 57 | 1.00 | 15 | 0.89 (0.49-1.59) | 0.68 |
|  | 25-<30 | 74 | 48 | 1.00 | 26 | 1.90 (1.02-3.53) | 0.04 |
|  | ≥30 | 34 | 23 | 1.00 | 11 | 0.77 (0.34-1.73) | 0.52 |
| Self-harm, interpersonal violence |  |  |  |  |  |  |  |
| Suicide | <25 | 68 | 55 | 1.00 | 13 | 1.52 (0.53-4.30) | 0.43 |
|  | 25-<30 | 50 | 40 | 1.00 | 10 | 0.75 (0.39-1.42) | 0.38 |
|  | ≥30 | 31 | 18 | 1.00 | 13 | 1.09 (0.57-2.09) | 0.79 |
| Homicide | <25 | 23 | 21 | 1.00 | 2 | 0.46 (0.09-2.34) | 0.35 |
|  | 25-<30 | 13 | 9 | 1.00 | 4 | 0.88 (0.19-4.02) | 0.87 |
|  | ≥30 | 19 | 15 | 1.00 | 4 | 0.46 (0.15-1.46) | 0.19 |
| All other diseases (residual) | <25 | 412 | 234 | 1.00 | 178 | 1.36 (1.04-1.77) | 0.02 |
|  | 25-<30 | 272 | 133 | 1.00 | 139 | 1.27 (0.94-1.71) | 0.12 |
|  | ≥30 | 180 | 67 | 1.00 | 113 | 1.45 (1.00-2.10) | 0.049 |
| All other causes/all unknown causes | <25 | 485 | 386 | 1.00 | 99 | 0.92 (0.68-1.23) | 0.57 |
|  | 25-<30 | 385 | 277 | 1.00 | 108 | 1.04 (0.81-1.35) | 0.36 |
|  | ≥30 | 201 | 121 | 1.00 | 80 | 1.20 (0.81-1.80) | 0.71 |

Multivariable adjustment for age, sex, education, race, income, alcohol, smoking status, BMI, physical activity, and survey year

Supplementary Table 9. Hazard ratios of all-cause mortality and cause-specific mortality participants with hypertension vs. no hypertension, stratified by physical activity

|  |  | Total | No hypertension | | Hypertension | |  |
| --- | --- | --- | --- | --- | --- | --- | --- |
|  | Physical activity | N  (deaths) | N  (deaths) | HR | N (deaths) | HR (95% CI) | P-value |
| All-cause mortality | Inactive | 7065 | 3688 | 1.00 | 3377 | 1.20 (1.13-1.27) | <0.0001 |
|  | Insufficient | 1679 | 886 | 1.00 | 793 | 1.35 (1.19-1.54) | <0.0001 |
|  | Sufficient | 2228 | 1379 | 1.00 | 849 | 1.34 (1.21-1.49) | <0.0001 |
| Infections |  |  |  |  |  |  |  |
| Septicemia | Inactive | 90 | 43 | 1.00 | 47 | 1.20 (0.68-2.13) | 0.53 |
|  | Insufficient | 23 | 11 | 1.00 | 12 | 1.94 (0.66-5.70) | 0.23 |
|  | Sufficient | 26 | 12 | 1.00 | 14 | 3.53 (1.38-8.99) | 0.008 |
| Viral hepatitis | Inactive | 14 | 8 | 1.00 | 6 | 1.22 (0.40-3.76) | 0.73 |
|  | Insufficient | 4 | 4 | 1.00 | 0 | - | - |
|  | Sufficient | 8 | 4 | 1.00 | 4 | 7.20 (1.16-44.65) | 0.03 |
| Human immunodeficiency virus | Inactive | 44 | 28 | 1.00 | 16 | 1.50 (0.60-3.72) | 0.39 |
|  | Insufficient | 14 | 10 | 1.00 | 4 | 0.93 (0.13-6.77) | 0.94 |
|  | Sufficient | 13 | 8 | 1.00 | 5 | 4.41 (1.05-18.45) | 0.04 |
| Other infectious parasitic disease | Inactive | 20 | 7 | 1.00 | 13 | 3.80 (1.20-12.08) | 0.02 |
|  | Insufficient | 5 | 2 | 1.00 | 3 | 1.23 (0.13-11.37) | 0.86 |
|  | Sufficient | 6 | 4 | 1.00 | 2 | 2.07 (0.42-10.22) | 0.37 |
| Cancers |  |  |  |  |  |  |  |
| All cancers | Inactive | 1265 | 716 | 1.00 | 549 | 0.99 (0.87-1.13) | 0.90 |
|  | Insufficient | 391 | 219 | 1.00 | 172 | 1.16 (0.89-1.52) | 0.27 |
|  | Sufficient | 598 | 376 | 1.00 | 222 | 1.11 (0.91-1.37) | 0.30 |
| Oral cavity, pharynx, lip | Inactive | 14 | 8 | 1.00 | 6 | 1.25 (0.42-3.73) | 0.68 |
|  | Insufficient | 2 | 0 | 1.00 | 2 | - | - |
|  | Sufficient | 7 | 7 | 1.00 | 0 | - | - |
| Esophagus | Inactive | 28 | 14 | 1.00 | 14 | 2.17 (0.84-5.63) | 0.11 |
|  | Insufficient | 10 | 7 | 1.00 | 3 | - | - |
|  | Sufficient | 12 | 7 | 1.00 | 5 | 1.08 (0.25-4.70) | 0.91 |
| Stomach | Inactive | 29 | 18 | 1.00 | 11 | 0.70 (0.29-1.67) | 0.42 |
|  | Insufficient | 10 | 6 | 1.00 | 4 | 1.48 (0.29-7.50) | 0.64 |
|  | Sufficient | 12 | 7 | 1.00 | 5 | 1.44 (0.42-4.88) | 0.56 |
| Colon, rectum, anus | Inactive | 122 | 69 | 1.00 | 53 | 0.89 (0.60-1.33) | 0.57 |
|  | Insufficient | 33 | 19 | 1.00 | 14 | 1.47 (0.58-3.70) | 0.41 |
|  | Sufficient | 54 | 34 | 1.00 | 20 | 1.28 (0.67-2.42) | 0.45 |
| Liver and bile ducts | Inactive | 33 | 21 | 1.00 | 12 | 0.55 (0.23-1.29) | 0.17 |
|  | Insufficient | 11 | 7 | 1.00 | 4 | 0.83 (0.21-3.29) | 0.79 |
|  | Sufficient | 21 | 11 | 1.00 | 10 | 1.86 (0.60-5.79) | 0.28 |
| Pancreas | Inactive | 69 | 36 | 1.00 | 33 | 0.86 (0.50-1.49) | 0.60 |
|  | Insufficient | 26 | 14 | 1.00 | 12 | 0.81 (0.36-1.82) | 0.61 |
|  | Sufficient | 52 | 30 | 1.00 | 22 | 1.33 (0.64-2.77) | 0.44 |
| Larynx | Inactive | 16 | 11 | 1.00 | 5 | 0.67 (0.19-2.28) | 0.52 |
|  | Insufficient | 1 | 0 | 1.00 | 1 | - | - |
|  | Sufficient | 3 | 2 | 1.00 | 1 | - | - |
| Lung, trachea, bronchus | Inactive | 435 | 245 | 1.00 | 190 | 1.18 (0.93-1.51) | 0.18 |
|  | Insufficient | 104 | 52 | 1.00 | 52 | 1.68 (1.04-2.71) | 0.03 |
|  | Sufficient | 179 | 115 | 1.00 | 64 | 1.13 (0.80-1.62) | 0.48 |
| Malignant melanoma | Inactive | 10 | 7 | 1.00 | 3 | 0.53 (0.16-1.71) | 0.29 |
|  | Insufficient | 6 | 5 | 1.00 | 1 | - | - |
|  | Sufficient | 9 | 5 | 1.00 | 4 | - | - |
| Breast (females) | Inactive | 77 | 43 | 1.00 | 34 | 0.93 (0.55-1.56) | 0.77 |
|  | Insufficient | 25 | 18 | 1.00 | 7 | 0.37 (0.11-1.25) | 0.11 |
|  | Sufficient | 25 | 17 | 1.00 | 8 | 1.57 (0.64-3.84) | 0.32 |
| Cervix uteri (females) | Inactive | 9 | 6 | 1.00 | 3 | 0.41 (0.10-1.76) | 0.23 |
|  | Insufficient | 4 | 2 | 1.00 | 2 | - | - |
|  | Sufficient | 6 | 3 | 1.00 | 3 | 1.77 (0.48-6.56) | 0.39 |
| Uterus (females) | Inactive | 19 | 13 | 1.00 | 6 | 0.47 (0.16-1.40) | 0.17 |
|  | Insufficient | 7 | 5 | 1.00 | 2 | - | - |
|  | Sufficient | 6 | 5 | 1.00 | 1 | 0.13 (0.01-1.42) | 0.09 |
| Ovaries (females) | Inactive | 26 | 14 | 1.00 | 12 | 1.00 (0.40-2.48) | 0.99 |
|  | Insufficient | 18 | 9 | 1.00 | 9 | 1.04 (0.34-3.17) | 0.94 |
|  | Sufficient | 17 | 12 | 1.00 | 5 | 0.65 (0.20-2.05) | 0.46 |
| Prostate (males) | Inactive | 49 | 27 | 1.00 | 22 | 0.80 (0.40-1.61) | 0.53 |
|  | Insufficient | 10 | 6 | 1.00 | 4 | 0.72 (0.14-3.81) | 0.70 |
|  | Sufficient | 26 | 15 | 1.00 | 11 | 1.18 (0.50-2.78) | 0.71 |
| Kidney and renal pelvis | Inactive | 34 | 22 | 1.00 | 12 | 0.66 (0.27-1.62) | 0.36 |
|  | Insufficient | 14 | 6 | 1.00 | 8 | 3.20 (0.79-13.00) | 0.10 |
|  | Sufficient | 9 | 3 | 1.00 | 6 | 4.71 (0.91-24.43) | 0.07 |
| Bladder | Inactive | 30 | 21 | 1.00 | 9 | 0.63 (0.24-1.64) | 0.34 |
|  | Insufficient | 7 | 6 | 1.00 | 1 | 0.11 (0.01-0.97) | 0.047 |
|  | Sufficient | 9 | 7 | 1.00 | 2 | 0.56 (0.12-2.57) | 0.46 |
| Brain, nervous system | Inactive | 20 | 8 | 1.00 | 12 | 2.06 (0.82-5.18) | 0.13 |
|  | Insufficient | 7 | 6 | 1.00 | 1 | 0.76 (0.05-11.45) | 0.85 |
|  | Sufficient | 12 | 7 | 1.00 | 5 | 1.17 (0.30-4.57) | 0.82 |
| Hodgkin's disease | Inactive | 1 | 1 | 1.00 | 0 | - | - |
|  | Insufficient | 5 | 3 | 1.00 | 2 | 1.07 (0.20-5.64) | 0.94 |
|  | Sufficient | 3 | 3 | 1.00 | 0 | - | - |
| Non-Hodgkin's lymphoma | Inactive | 34 | 15 | 1.00 | 19 | 1.49 (0.63-3.49) | 0.36 |
|  | Insufficient | 17 | 9 | 1.00 | 8 | 1.56 (0.54-4.50) | 0.41 |
|  | Sufficient | 15 | 10 | 1.00 | 5 | - | - |
| Leukemia | Inactive | 46 | 30 | 1.00 | 16 | 0.79 (0.40-1.56) | 0.50 |
|  | Insufficient | 12 | 8 | 1.00 | 4 | 0.96 (0.22-4.21) | 0.96 |
|  | Sufficient | 26 | 17 | 1.00 | 9 | 0.63 (0.24-1.66) | 0.35 |
| Multiple myeloma | Inactive | 20 | 9 | 1.00 | 11 | 0.88 (0.38-2.02) | 0.76 |
|  | Insufficient | 5 | 2 | 1.00 | 3 | 2.48 (0.25-24.68) | 0.44 |
|  | Sufficient | 12 | 9 | 1.00 | 3 | 0.37 (0.10-1.29) | 0.12 |
| All other and unspecified neoplasms | Inactive | 145 | 79 | 1.00 | 66 | 1.03 (0.70-1.52) | 0.88 |
|  | Insufficient | 57 | 29 | 1.00 | 28 | 1.61 (0.78-3.32) | 0.20 |
|  | Sufficient | 83 | 50 | 1.00 | 33 | 1.23 (0.71-2.15) | 0.46 |
| In situ and benign neoplasms | Inactive | 29 | 16 | 1.00 | 13 | 0.62 (0.26-1.51) | 0.29 |
|  | Insufficient | 8 | 4 | 1.00 | 4 | 1.40 (0.25-7.89) | 0.70 |
|  | Sufficient | 11 | 8 | 1.00 | 3 | 0.55 (0.12-2.49) | 0.43 |
| Endocrine, nutritional, metabolic diseases |  |  |  |  |  |  |  |
| Anaemia | Inactive | 10 | 8 | 1.00 | 2 | 0.06 (0.01-0.32) | 0.001 |
|  | Insufficient | 4 | 0 | 1.00 | 4 | - | - |
|  | Sufficient | 3 | 2 | 1.00 | 1 | 3.13 (0.26-37.81) | 0.37 |
| Diabetes mellitus | Inactive | 217 | 71 | 1.00 | 146 | 1.77 (1.23-2.53) | 0.002 |
|  | Insufficient | 42 | 20 | 1.00 | 22 | 1.34 (0.63-2.82) | 0.45 |
|  | Sufficient | 52 | 21 | 1.00 | 31 | 3.59 (1.80-7.18) | <0.0001 |
| Malnutrition | Inactive | 10 | 7 | 1.00 | 3 | 0.29 (0.07-1.11) | 0.07 |
|  | Insufficient | 3 | 1 | 1.00 | 2 | - | - |
|  | Sufficient | 1 | 1 | 1.00 | 0 | - | - |
| Nervous system |  |  |  |  |  |  |  |
| Parkinson's disease | Inactive | 33 | 14 | 1.00 | 19 | 1.72 (0.80-3.66) | 0.16 |
|  | Insufficient | 13 | 9 | 1.00 | 4 | 0.37 (0.12-1.09) | 0.07 |
|  | Sufficient | 14 | 11 | 1.00 | 3 | 0.56 (0.15-2.10) | 0.39 |
| Alzheimer's disease | Inactive | 134 | 84 | 1.00 | 50 | 0.64 (0.41-1.00) | 0.05 |
|  | Insufficient | 36 | 22 | 1.00 | 14 | 0.66 (0.28-1.59) | 0.36 |
|  | Sufficient | 35 | 20 | 1.00 | 15 | 0.75 (0.36-1.59) | 0.45 |
| Circulatory disease |  |  |  |  |  |  |  |
| All circulatory diseases | Inactive | 2542 | 1149 | 1.00 | 1393 | 1.38 (1.26-1.53) | <0.0001 |
|  | Insufficient | 558 | 249 | 1.00 | 309 | 1.69 (1.37-2.09) | <0.0001 |
|  | Sufficient | 626 | 312 | 1.00 | 314 | 1.74 (1.42-2.13) | <0.0001 |
| Hypertensive heart disease | Inactive | 82 | 25 | 1.00 | 57 | 2.50 (1.40-4.46) | 0.002 |
|  | Insufficient | 21 | 8 | 1.00 | 13 | 4.09 (1.29-12.98) | 0.02 |
|  | Sufficient | 25 | 12 | 1.00 | 13 | 3.18 (0.97-10.46) | 0.06 |
| Hypertensive heart and renal disease | Inactive | 12 | 3 | 1.00 | 9 | 2.12 (0.49-9.28) | 0.32 |
|  | Insufficient | 2 | 1 | 1.00 | 1 | - | - |
|  | Sufficient | 2 | 0 | 1.00 | 2 | - | - |
| Ischaemic heart disease | Inactive | 1364 | 648 | 1.00 | 716 | 1.30 (1.14-1.47) | <0.0001 |
|  | Insufficient | 296 | 143 | 1.00 | 153 | 1.37 (1.03-1.81) | 0.03 |
|  | Sufficient | 341 | 177 | 1.00 | 164 | 1.50 (1.17-1.92) | 0.001 |
| Acute myocardial infarction | Inactive | 526 | 238 | 1.00 | 288 | 1.43 (1.16-1.77) | 0.001 |
|  | Insufficient | 108 | 53 | 1.00 | 55 | 1.58 (0.96-2.62) | 0.07 |
|  | Sufficient | 124 | 58 | 1.00 | 66 | 1.77 (1.16-2.69) | 0.008 |
| Other acute ischaemic heart disease | Inactive | 7 | 3 | 1.00 | 4 | 1.28 (0.31-5.31) | 0.73 |
|  | Insufficient | 1 | 0 | 1.00 | 1 | - | - |
|  | Sufficient | 6 | 1 | 1.00 | 5 | - | - |
| Atherosclerotic cardiovascular disease | Inactive | 188 | 108 | 1.00 | 80 | 0.92 (0.64-1.33) | 0.67 |
|  | Insufficient | 49 | 28 | 1.00 | 21 | 0.94 (0.50-1.75) | 0.84 |
|  | Sufficient | 56 | 36 | 1.00 | 20 | 0.75 (0.40-1.41) | 0.38 |
| Other chronic ischaemic heart disease | Inactive | 643 | 299 | 1.00 | 344 | 1.31 (1.09-1.58) | 0.004 |
|  | Insufficient | 138 | 62 | 1.00 | 76 | 1.36 (0.94-1.97) | 0.10 |
|  | Sufficient | 155 | 82 | 1.00 | 73 | 1.45 (1.00-2.11) | 0.05 |
| Heart failure | Inactive | 172 | 80 | 1.00 | 92 | 1.20 (0.83-1.73) | 0.32 |
|  | Insufficient | 33 | 15 | 1.00 | 18 | 1.09 (0.52-2.30) | 0.82 |
|  | Sufficient | 28 | 14 | 1.00 | 14 | 1.27 (0.52-3.12) | 0.59 |
| All other forms of heart disease | Inactive | 307 | 142 | 1.00 | 165 | 1.37 (1.03-1.83) | 0.03 |
|  | Insufficient | 78 | 34 | 1.00 | 44 | 1.88 (1.14-3.11) | 0.01 |
|  | Sufficient | 82 | 43 | 1.00 | 39 | 1.76 (0.95-3.27) | 0.07 |
| Primary hypertension, renal disease | Inactive | 89 | 29 | 1.00 | 60 | 2.36 (1.32-4.22) | 0.004 |
|  | Insufficient | 12 | 4 | 1.00 | 8 | 12.16 (2.05-72.24) | 0.006 |
|  | Sufficient | 11 | 2 | 1.00 | 9 | 8.64 (2.14-34.98) | 0.003 |
| Cerebrovascular disease | Inactive | 397 | 166 | 1.00 | 231 | 1.50 (1.18-1.92) | 0.001 |
|  | Insufficient | 94 | 35 | 1.00 | 59 | 2.49 (1.56-3.97) | <0.0001 |
|  | Sufficient | 112 | 55 | 1.00 | 57 | 1.79 (1.15-2.77) | 0.009 |
| Atherosclerosis | Inactive | 22 | 11 | 1.00 | 11 | 1.37 (0.50-3.78) | 0.54 |
|  | Insufficient | 3 | 1 | 1.00 | 2 | - | - |
|  | Sufficient | 4 | 2 | 1.00 | 2 | - | - |
| Other diseases of circulatory system | Inactive | 97 | 45 | 1.00 | 52 | 1.38 (0.84-2.27) | 0.21 |
|  | Insufficient | 19 | 8 | 1.00 | 11 | 1.51 (0.41-5.52) | 0.54 |
|  | Sufficient | 21 | 7 | 1.00 | 14 | 4.84 (1.27-18.43) | 0.02 |
| Aortic aneurysm and dissection | Inactive | 50 | 22 | 1.00 | 28 | 1.42 (0.72-2.80) | 0.31 |
|  | Insufficient | 12 | 5 | 1.00 | 7 | 1.27 (0.26-6.19) | 0.76 |
|  | Sufficient | 11 | 4 | 1.00 | 7 | 4.01 (1.17-13.73) | 0.03 |
| Other diseases of arteries or capillaries | Inactive | 36 | 18 | 1.00 | 18 | 1.07 (0.49-2.36) | 0.86 |
|  | Insufficient | 4 | 1 | 1.00 | 3 | 2.51 (0.17-38.06) | 0.51 |
|  | Sufficient | 6 | 2 | 1.00 | 4 | 9.41 (0.56-159.24) | 0.12 |
| Other disorders of circulatory system | Inactive | 11 | 5 | 1.00 | 6 | 2.85 (0.51-15.73) | 0.23 |
|  | Insufficient | 3 | 2 | 1.00 | 1 | - | - |
|  | Sufficient | 4 | 1 | 1.00 | 3 | - | - |
| Respiratory diseases |  |  |  |  |  |  |  |
| Pneumonia | Inactive | 177 | 96 | 1.00 | 81 | 1.01 (0.70-1.45) | 0.97 |
|  | Insufficient | 24 | 13 | 1.00 | 11 | 0.95 (0.39-2.31) | 0.91 |
|  | Sufficient | 28 | 21 | 1.00 | 7 | 0.70 (0.30-1.64) | 0.41 |
| Emphysema | Inactive | 45 | 27 | 1.00 | 18 | 1.17 (0.56-2.45) | 0.68 |
|  | Insufficient | 7 | 4 | 1.00 | 3 | 0.74 (0.15-3.58) | 0.71 |
|  | Sufficient | 9 | 4 | 1.00 | 5 | 1.88 (0.30-11.94) | 0.50 |
| Other chronic lower respiratory disease | Inactive | 370 | 195 | 1.00 | 175 | 1.36 (1.07-1.73) | 0.01 |
|  | Insufficient | 54 | 24 | 1.00 | 30 | 1.71 (0.89-3.27) | 0.11 |
|  | Sufficient | 57 | 34 | 1.00 | 23 | 1.11 (0.60-2.04) | 0.74 |
| Pneumonitis from solids, liquids | Inactive | 48 | 33 | 1.00 | 15 | 0.60 (0.26-1.35) | 0.22 |
|  | Insufficient | 18 | 8 | 1.00 | 10 | 1.31 (0.45-3.82) | 0.62 |
|  | Sufficient | 6 | 5 | 1.00 | 1 | - | - |
| Other respiratory system diseases | Inactive | 93 | 48 | 1.00 | 45 | 1.01 (0.60-1.69) | 0.98 |
|  | Insufficient | 20 | 8 | 1.00 | 12 | 1.97 (0.72-5.40) | 0.19 |
|  | Sufficient | 20 | 13 | 1.00 | 7 | 1.15 (0.24-5.46) | 0.86 |
| Digestive diseases |  |  |  |  |  |  |  |
| Alcoholic liver disease | Inactive | 49 | 31 | 1.00 | 18 | 1.82 (0.79-4.18) | 0.16 |
|  | Insufficient | 15 | 8 | 1.00 | 7 | 3.23 (1.02-10.21) | 0.045 |
|  | Sufficient | 13 | 9 | 1.00 | 4 | 0.83 (0.18-3.77) | 0.81 |
| Other chronic liver disease | Inactive | 60 | 30 | 1.00 | 30 | 1.61 (0.82-3.19) | 0.17 |
|  | Insufficient | 11 | 9 | 1.00 | 2 | 1.23 (0.25-5.96) | 0.80 |
|  | Sufficient | 15 | 7 | 1.00 | 8 | 4.10 (0.83-20.20) | 0.08 |
| Cholelithiasis, gallbladder disease | Inactive | 12 | 4 | 1.00 | 8 | 1.36 (0.31-6.06) | 0.68 |
|  | Insufficient | 0 | 0 | 1.00 | 0 | - | - |
|  | Sufficient | 0 | 0 | 1.00 | 0 | - | - |
| Urinary tract disease |  |  |  |  |  |  |  |
| Kidney failure | Inactive | 140 | 54 | 1.00 | 86 | 1.86 (1.23-2.80) | 0.003 |
|  | Insufficient | 20 | 6 | 1.00 | 14 | 2.24 (0.70-7.14) | 0.17 |
|  | Sufficient | 20 | 9 | 1.00 | 11 | 1.76 (0.68-4.55) | 0.24 |
| Abnormal clinical, lab findings | Inactive | 77 | 37 | 1.00 | 40 | 1.45 (0.87-2.41) | 0.15 |
|  | Insufficient | 17 | 9 | 1.00 | 8 | 1.11 (0.39-3.14) | 0.85 |
|  | Sufficient | 20 | 18 | 1.00 | 2 | 0.17 (0.04-0.74) | 0.02 |
| Transport injuries |  |  |  |  |  |  |  |
| Motor vehicle accidents | Inactive | 69 | 46 | 1.00 | 23 | 1.75 (0.95-3.21) | 0.07 |
|  | Insufficient | 27 | 18 | 1.00 | 9 | 2.49 (1.19-5.23) | 0.02 |
|  | Sufficient | 72 | 58 | 1.00 | 14 | 1.21 (0.56-2.64) | 0.62 |
| Unintentional injuries |  |  |  |  |  |  |  |
| Falls | Inactive | 46 | 26 | 1.00 | 20 | 0.95 (0.52-1.72) | 0.86 |
|  | Insufficient | 11 | 9 | 1.00 | 2 | 0.28 (0.06-1.36) | 0.12 |
|  | Sufficient | 22 | 15 | 1.00 | 7 | 1.99 (0.49-8.06) | 0.33 |
| Other non-transport accidents combined | Inactive | 116 | 79 | 1.00 | 37 | 1.07 (0.69-1.67) | 0.75 |
|  | Insufficient | 25 | 14 | 1.00 | 11 | 1.72 (0.75-3.93) | 0.20 |
|  | Sufficient | 42 | 34 | 1.00 | 8 | 1.53 (0.58-4.03) | 0.39 |
| Self-harm, interpersonal violence |  |  |  |  |  |  |  |
| Suicide | Inactive | 69 | 53 | 1.00 | 16 | 0.81 (0.38-1.72) | 0.58 |
|  | Insufficient | 23 | 13 | 1.00 | 10 | 1.26 (0.56-2.85) | 0.58 |
|  | Sufficient | 54 | 45 | 1.00 | 9 | 1.16 (0.38-3.60) | 0.79 |
| Homicide | Inactive | 33 | 25 | 1.00 | 8 | 0.54 (0.22-1.36) | 0.19 |
|  | Insufficient | 6 | 4 | 1.00 | 2 | 2.37 (0.22-25.66) | 0.48 |
|  | Sufficient | 14 | 14 | 1.00 | 0 | - | - |
| All other diseases (residual) | Inactive | 602 | 296 | 1.00 | 306 | 1.27 (1.03-1.57) | 0.03 |
|  | Insufficient | 131 | 67 | 1.00 | 64 | 1.27 (0.83-1.94) | 0.27 |
|  | Sufficient | 151 | 83 | 1.00 | 68 | 1.49 (0.98-2.28) | 0.06 |
| All other causes/all unknown causes | Inactive | 651 | 457 | 1.00 | 194 | 0.98 (0.79-1.21) | 0.85 |
|  | Insufficient | 169 | 121 | 1.00 | 48 | 1.05 (0.71-1.55) | 0.80 |
|  | Sufficient | 282 | 231 | 1.00 | 61 | 1.01 (0.74-1.39) | 0.92 |

Multivariable adjustment for age, sex, education, race, income, alcohol, smoking status, BMI, physical activity, and survey year

Supplementary Table 10. Hazard ratios of all-cause mortality and cause-specific mortality among participants with hypertension vs. no hypertension, stratified by smoking status

|  |  | Total | No hypertension | | Hypertension | |  |
| --- | --- | --- | --- | --- | --- | --- | --- |
|  | Smoking status | N  (deaths) | N  (deaths) | HR | N (deaths) | HR (95% CI) | P-value |
| All-cause mortality | Never | 4951 | 2625 | 1.00 | 2326 | 1.21 (1.12-1.30) | <0.0001 |
|  | Former | 3398 | 1721 | 1.00 | 1677 | 1.19 (1.10-1.29) | <0.0001 |
|  | Current | 2824 | 1725 | 1.00 | 1099 | 1.43 (1.30-1.57) | <0.0001 |
| Infections |  |  |  |  |  |  |  |
| Septicemia | Never | 49 | 23 | 1.00 | 26 | 1.85 (0.76-4.48) | 0.17 |
|  | Former | 57 | 27 | 1.00 | 30 | 1.41 (0.77-2.61) | 0.27 |
|  | Current | 33 | 15 | 1.00 | 18 | 2.20 (0.92-5.25) | 0.08 |
| Viral hepatitis | Never | 8 | 5 | 1.00 | 3 | 2.51 (0.29-21.66) | 0.40 |
|  | Former | 7 | 6 | 1.00 | 1 | - | - |
|  | Current | 12 | 7 | 1.00 | 5 | 1.75 (0.45-6.89) | 0.42 |
| Human immunodeficiency virus | Never | 21 | 13 | 1.00 | 8 | 2.44 (0.41-14.60) | 0.33 |
|  | Former | 13 | 12 | 1.00 | 1 | 0.06 (0.01-0.47) | 0.008 |
|  | Current | 38 | 22 | 1.00 | 16 | 2.38 (0.89-6.40) | 0.09 |
| Other infectious parasitic disease | Never | 14 | 6 | 1.00 | 8 | 1.24 (0.39-3.88) | 0.72 |
|  | Former | 8 | 2 | 1.00 | 6 | 8.07 (1.64-39.65) | 0.01 |
|  | Current | 9 | 5 | 1.00 | 4 | 3.21 (0.51-20.11) | 0.21 |
| Cancers |  |  |  |  |  |  |  |
| All cancers | Never | 804 | 452 | 1.00 | 352 | 0.98 (0.82-1.17) | 0.81 |
|  | Former | 766 | 409 | 1.00 | 338 | 1.05 (0.88-1.25) | 0.62 |
|  | Current | 761 | 481 | 1.00 | 267 | 1.12 (0.92-1.37) | 0.29 |
| Oral cavity, pharynx, lip | Never | 5 | 2 | 1.00 | 3 | - | - |
|  | Former | 8 | 6 | 1.00 | 2 | 0.36 (0.05-2.30) | 0.28 |
|  | Current | 14 | 9 | 1.00 | 5 | 1.22 (0.42-3.56) | 0.72 |
| Esophagus | Never | 17 | 11 | 1.00 | 6 | 1.05 (0.32-3.46) | 0.94 |
|  | Former | 20 | 10 | 1.00 | 10 | 1.36 (0.49-3.73) | 0.55 |
|  | Current | 18 | 10 | 1.00 | 8 | 1.92 (0.48-7.62) | 0.35 |
| Stomach | Never | 22 | 15 | 1.00 | 7 | 0.50 (0.17-1.49) | 0.21 |
|  | Former | 17 | 9 | 1.00 | 8 | 0.99 (0.36-2.77) | 0.99 |
|  | Current | 13 | 8 | 1.00 | 5 | 2.17 (0.62-7.54) | 0.22 |
| Colon, rectum, anus | Never | 102 | 55 | 1.00 | 47 | 1.10 (0.68-1.76) | 0.70 |
|  | Former | 64 | 36 | 1.00 | 28 | 0.97 (0.53-1.78) | 0.92 |
|  | Current | 47 | 34 | 1.00 | 13 | 0.93 (0.45-1.90) | 0.84 |
| Liver and bile ducts | Never | 26 | 16 | 1.00 | 10 | 1.31 (0.45-3.78) | 0.62 |
|  | Former | 25 | 14 | 1.00 | 11 | 0.66 (0.25-1.76) | 0.40 |
|  | Current | 17 | 12 | 1.00 | 5 | 0.65 (0.22-1.96) | 0.45 |
| Pancreas | Never | 71 | 34 | 1.00 | 37 | 1.03 (0.59-1.80) | 0.91 |
|  | Former | 45 | 25 | 1.00 | 20 | 1.18 (0.60-2.32) | 0.62 |
|  | Current | 33 | 22 | 1.00 | 11 | 0.73 (0.29-1.83) | 0.50 |
| Larynx | Never | 1 | 1 | 1.00 | 0 | - | - |
|  | Former | 7 | 3 | 1.00 | 4 | 2.14 (0.32-14.31) | 0.43 |
|  | Current | 12 | 9 | 1.00 | 3 | 0.43 (0.10-1.87) | 0.26 |
| Lung, trachea, bronchus | Never | 98 | 48 | 1.00 | 50 | 1.64 (0.98-2.75) | 0.06 |
|  | Former | 247 | 130 | 1.00 | 117 | 1.18 (0.89-1.57) | 0.25 |
|  | Current | 386 | 244 | 1.00 | 142 | 1.18 (0.90-1.56) | 0.23 |
| Malignant melanoma | Never | 10 | 7 | 1.00 | 3 | 1.82 (0.34-9.79) | 0.48 |
|  | Former | 12 | 8 | 1.00 | 4 | 0.44 (0.12-1.61) | 0.21 |
|  | Current | 3 | 2 | 1.00 | 1 | - | - |
| Breast (females) | Never | 76 | 45 | 1.00 | 31 | 0.90 (0.50-1.62) | 0.72 |
|  | Former | 24 | 16 | 1.00 | 8 | 0.46 (0.18-1.16) | 0.10 |
|  | Current | 28 | 18 | 1.00 | 10 | 1.19 (0.45-3.14) | 0.72 |
| Cervix uteri (females) | Never | 7 | 4 | 1.00 | 3 | 0.53 (0.11-2.56) | 0.43 |
|  | Former | 2 | 0 | 1.00 | 2 | - | - |
|  | Current | 10 | 7 | 1.00 | 3 | 0.68 (0.18-2.63) | 0.58 |
| Uterus (females) | Never | 25 | 17 | 1.00 | 8 | 0.45 (0.17-1.19) | 0.11 |
|  | Former | 5 | 5 | 1.00 | 0 | - | - |
|  | Current | 2 | 1 | 1.00 | 1 | 1.33 (0.22-7.89) | 0.75 |
| Ovaries (females) | Never | 36 | 23 | 1.00 | 13 | 0.80 (0.35-1.84) | 0.60 |
|  | Former | 19 | 12 | 1.00 | 7 | 0.54 (0.24-1.24) | 0.15 |
|  | Current | 7 | 1 | 1.00 | 6 | 6.85 (1.36-34.53) | 0.02 |
| Prostate (males) | Never | 31 | 20 | 1.00 | 11 | 0.46 (0.19-1.11) | 0.08 |
|  | Former | 38 | 20 | 1.00 | 18 | 1.13 (0.53-2.37) | 0.75 |
|  | Current | 17 | 9 | 1.00 | 8 | - | - |
| Kidney and renal pelvis | Never | 21 | 11 | 1.00 | 10 | 1.93 (0.68-6.40) | 0.28 |
|  | Former | 23 | 12 | 1.00 | 11 | 0.82 (0.30-2.24) | 0.70 |
|  | Current | 14 | 9 | 1.00 | 5 | 2.78 (0.73-10.54) | 0.13 |
| Bladder | Never | 13 | 11 | 1.00 | 2 | 0.12 (0.03-0.55) | 0.007 |
|  | Former | 22 | 13 | 1.00 | 9 | 0.88 (0.32-2.41) | 0.80 |
|  | Current | 13 | 11 | 1.00 | 2 | 0.48 (0.10-2.30) | 0.36 |
| Brain, nervous system | Never | 17 | 9 | 1.00 | 8 | 0.82 (0.32-2.12) | 0.68 |
|  | Former | 14 | 9 | 1.00 | 5 | 1.16 (0.32-4.18) | 0.82 |
|  | Current | 10 | 5 | 1.00 | 5 | 3.34 (0.96-11.61) | 0.06 |
| Hodgkin's disease | Never | 5 | 3 | 1.00 | 2 | 1.05 (0.16-6.77) | 0.96 |
|  | Former | 0 | 1 | 0 | - | - | - |
|  | Current | 3 | 3 | 1.00 | 0 | - | - |
| Non-Hodgkin's lymphoma | Never | 36 | 19 | 1.00 | 17 | 0.98 (0.49-1.98) | 0.96 |
|  | Former | 21 | 10 | 1.00 | 11 | 2.68 (0.92-7.75) | 0.07 |
|  | Current | 10 | 5 | 1.00 | 5 | 1.09 (0.24-4.82) | 0.91 |
| Leukemia | Never | 41 | 25 | 1.00 | 16 | 0.78 (0.38-1.63) | 0.51 |
|  | Former | 37 | 22 | 1.00 | 15 | 0.93 (0.44-1.96) | 0.84 |
|  | Current | 9 | 9 | 1.00 | 0 | - | - |
| Multiple myeloma | Never | 22 | 11 | 1.00 | 11 | 0.88 (0.38-2.08) | 0.78 |
|  | Former | 12 | 7 | 1.00 | 5 | 0.56 (0.20-1.55) | 0.26 |
|  | Current | 3 | 2 | 1.00 | 1 | - | - |
| All other and unspecified neoplasms | Never | 122 | 65 | 1.00 | 57 | 1.11 (0.69-1.80) | 0.66 |
|  | Former | 85 | 42 | 1.00 | 43 | 1.34 (0.81-2.24) | 0.26 |
|  | Current | 79 | 51 | 1.00 | 28 | 1.06 (0.60-1.85) | 0.85 |
| In situ and benign neoplasms | Never | 18 | 8 | 1.00 | 10 | 1.11 (0.40-3.09) | 0.85 |
|  | Former | 19 | 13 | 1.00 | 6 | 0.42 (0.10-1.71) | 0.22 |
|  | Current | 13 | 9 | 1.00 | 4 | 0.81 (0.17-3.74) | 0.78 |
| Endocrine, nutritional, metabolic diseases |  |  |  |  |  |  |  |
| Anaemia | Never | 12 | 9 | 1.00 | 3 | 0.25 (0.04-1.70) | 0.16 |
|  | Former | 5 | 1 | 1.00 | 4 | 15.64 (1.28-191.07) | 0.03 |
|  | Current | 0 | 0 | 1.00 | 0 | - | - |
| Diabetes mellitus | Never | 154 | 54 | 1.00 | 100 | 1.78 (1.10-2.89) | 0.02 |
|  | Former | 104 | 33 | 1.00 | 71 | 2.15 (1.34-3.46) | 0.002 |
|  | Current | 58 | 24 | 1.00 | 34 | 2.29 (1.15-4.55) | 0.02 |
| Malnutrition | Never | 8 | 6 | 1.00 | 2 | 0.17 (0.03-1.02) | 0.05 |
|  | Former | 6 | 3 | 1.00 | 3 | - | - |
|  | Current | 0 | 0 | 1.00 | 0 | - | - |
| Nervous system |  |  |  |  |  |  |  |
| Parkinson's disease | Never | 38 | 23 | 1.00 | 15 | 0.91 (0.45-1.84) | 0.79 |
|  | Former | 20 | 10 | 1.00 | 10 | 1.49 (0.54-4.10) | 0.43 |
|  | Current | 3 | 1 | 1.00 | 2 | - | - |
| Alzheimer's disease | Never | 124 | 76 | 1.00 | 48 | 0.70 (0.46-1.07) | 0.10 |
|  | Former | 68 | 43 | 1.00 | 25 | 0.51(0.27-0.96) | 0.04 |
|  | Current | 14 | 9 | 1.00 | 5 | 0.82 (0.24-2.83) | 0.75 |
| Circulatory disease |  |  |  |  |  |  |  |
| All circulatory diseases | Never | 1851 | 813 | 1.00 | 1038 | 1.48 (1.31-1.67) | <0.0001 |
|  | Former | 1164 | 531 | 1.00 | 633 | 1.37 (1.19-1.57) | <0.0001 |
|  | Current | 782 | 399 | 1.00 | 383 | 1.76 (1.48-2.10) | <0.0001 |
| Hypertensive heart disease | Never | 69 | 26 | 1.00 | 43 | 1.98 (1.06-3.67) | 0.03 |
|  | Former | 33 | 10 | 1.00 | 23 | 3.74 (1.48-9.48) | 0.006 |
|  | Current | 31 | 9 | 1.00 | 22 | 8.12 (3.17-20.80) | <0.0001 |
| Hypertensive heart and renal disease | Never | 10 | 0 | 1.00 | 10 | - | - |
|  | Former | 4 | 4 | 1.00 | 0 | - | - |
|  | Current | 2 | 0 | 1.00 | 2 | - | - |
| Ischaemic heart disease | Never | 920 | 432 | 1.00 | 488 | 1.31 (1.11-1.55) | 0.002 |
|  | Former | 660 | 315 | 1.00 | 345 | 1.24 (1.05-1.47) | 0.01 |
|  | Current | 458 | 237 | 1.00 | 221 | 1.65 (1.34-2.04) | <0.0001 |
| Acute myocardial infarction | Never | 339 | 145 | 1.00 | 194 | 1.64 (1.22-2.22) | 0.001 |
|  | Former | 247 | 115 | 1.00 | 132 | 1.27 (0.94-1.71) | 0.11 |
|  | Current | 187 | 96 | 1.00 | 91 | 1.67 (1.17-2.39) | 0.005 |
| Other acute ischaemic heart disease | Never | 4 | 2 | 1.00 | 2 | 0.72 (0.06-8.28) | 0.79 |
|  | Former | 5 | 1 | 1.00 | 4 | 3.15 (0.40-25.03) | 0.28 |
|  | Current | 6 | 2 | 1.00 | 4 | - | - |
| Atherosclerotic cardiovascular disease | Never | 131 | 74 | 1.00 | 57 | 0.93 (0.60-1.45) | 0.76 |
|  | Former | 87 | 51 | 1.00 | 36 | 0.87 (0.49-1.57) | 0.65 |
|  | Current | 82 | 50 | 1.00 | 32 | 1.01 (0.60-1.71) | 0.005 |
| Other chronic ischaemic heart disease | Never | 446 | 211 | 1.00 | 235 | 1.22 (0.98-1.53) | 0.08 |
|  | Former | 321 | 148 | 1.00 | 173 | 1.30 (1.01-1.68) | 0.04 |
|  | Current | 183 | 89 | 1.00 | 94 | 1.91 (1.36-2.67) | <0.0001 |
| Heart failure | Never | 136 | 60 | 1.00 | 76 | 1.34 (0.90-1.99) | 0.14 |
|  | Former | 72 | 32 | 1.00 | 40 | 1.39 (0.80-2.40) | 0.24 |
|  | Current | 24 | 16 | 1.00 | 8 | 0.49 (0.18-1.34) | 0.17 |
| All other forms of heart disease | Never | 254 | 110 | 1.00 | 144 | 1.68 (1.24-2.28) | 0.001 |
|  | Former | 138 | 71 | 1.00 | 67 | 1.02 (0.70-1.49) | 0.92 |
|  | Current | 85 | 43 | 1.00 | 42 | 2.43 (1.43-4.12) | 0.001 |
| Primary hypertension, renal disease | Never | 66 | 23 | 1.00 | 43 | 2.22 (1.07-4.64) | 0.03 |
|  | Former | 34 | 10 | 1.00 | 24 | 4.10 (1.72-9.78) | 0.002 |
|  | Current | 14 | 3 | 1.00 | 11 | 6.11 (1.74-21.50) | 0.005 |
| Cerebrovascular disease | Never | 319 | 131 | 1.00 | 188 | 1.52 (1.14-2.02) | 0.005 |
|  | Former | 171 | 63 | 1.00 | 108 | 2.00 (1.37-2.92) | <0.0001 |
|  | Current | 126 | 71 | 1.00 | 55 | 1.61 (1.04-2.51) | 0.03 |
| Atherosclerosis | Never | 15 | 5 | 1.00 | 10 | 2.71 (0.91-8.06) | 0.07 |
|  | Former | 13 | 8 | 1.00 | 5 | 1.08 (0.24-4.80) | 0.92 |
|  | Current | 2 | 1 | 1.00 | 1 | - | - |
| Other diseases of circulatory system | Never | 62 | 26 | 1.00 | 36 | 2.02 (0.97-4.18) | 0.06 |
|  | Former | 39 | 18 | 1.00 | 21 | 1.52 (0.58-3.96) | 0.39 |
|  | Current | 40 | 19 | 1.00 | 21 | 1.54 (0.70-3.38) | 0.28 |
| Aortic aneurysm and dissection | Never | 25 | 9 | 1.00 | 34 | 2.22 (0.72-6.83) | 0.16 |
|  | Former | 25 | 13 | 1.00 | 12 | 0.92 (0.35-2.41) | 0.87 |
|  | Current | 24 | 10 | 1.00 | 14 | 1.86 (0.72-4.83) | 0.20 |
| Other diseases of arteries or capillaries | Never | 21 | 8 | 1.00 | 13 | 2.39 (0.71-8.04) | 0.16 |
|  | Former | 11 | 5 | 1.00 | 6 | 2.93 (0.36-23.64) | 0.31 |
|  | Current | 15 | 9 | 1.00 | 6 | 0.56 (0.13-2.41) | 0.44 |
| Other disorders of circulatory system | Never | 16 | 9 | 1.00 | 7 | 1.25 (0.32-4.97) | 0.75 |
|  | Former | 3 | 0 | 1.00 | 3 | - | - |
|  | Current | 1 | 0 | 1.00 | 1 | - | - |
| Respiratory diseases |  |  |  |  |  |  |  |
| Pneumonia | Never | 124 | 72 | 1.00 | 52 | 0.79 (0.52-1.19) | 0.25 |
|  | Former | 63 | 28 | 1.00 | 35 | 1.53 (0.82-2.86) | 0.18 |
|  | Current | 49 | 35 | 1.00 | 14 | 0.73 (0.37-1.46) | 0.37 |
| Emphysema | Never | 6 | 4 | 1.00 | 2 | 0.38 (0.06-2.59) | 0.33 |
|  | Former | 29 | 17 | 1.00 | 12 | 0.87 (0.34-2.26) | 0.78 |
|  | Current | 27 | 15 | 1.00 | 12 | 1.80 (0.74-4.41) | 0.20 |
| Other chronic lower respiratory disease | Never | 65 | 29 | 1.00 | 36 | 2.07 (1.08-3.99) | 0.03 |
|  | Former | 242 | 131 | 1.00 | 111 | 1.04 (0.74-1.45) | 0.83 |
|  | Current | 183 | 97 | 1.00 | 86 | 1.88 (1.34-2.63) | <0.0001 |
| Pneumonitis from solids, liquids | Never | 35 | 22 | 1.00 | 13 | 0.63 (0.28-1.42) | 0.27 |
|  | Former | 28 | 18 | 1.00 | 10 | 0.85 (0.34-2.13) | 0.73 |
|  | Current | 8 | 6 | 1.00 | 2 | - | - |
| Other respiratory system diseases | Never | 59 | 26 | 1.00 | 33 | 1.26 (0.73-2.18) | 0.40 |
|  | Former | 53 | 33 | 1.00 | 20 | 0.65 (0.32-1.32) | 0.23 |
|  | Current | 28 | 15 | 1.00 | 13 | 3.09 (1.05-9.09) | 0.04 |
| Digestive diseases |  |  |  |  |  |  |  |
| Alcoholic liver disease | Never | 20 | 14 | 1.00 | 6 | 1.39 (0.37-5.25) | 0.63 |
|  | Former | 14 | 6 | 1.00 | 8 | 0.87 (0.34-2.26) | 0.78 |
|  | Current | 46 | 31 | 1.00 | 15 | 1.37 (0.59-3.15) | 0.46 |
| Other chronic liver disease | Never | 26 | 14 | 1.00 | 12 | 1.84 (0.64-5.28) | 0.25 |
|  | Former | 27 | 11 | 1.00 | 16 | 3.69 (0.85-16.08) | 0.08 |
|  | Current | 34 | 21 | 1.00 | 13 | 1.99 (0.70-5.64) | 0.20 |
| Cholelithiasis, gallbladder disease | Never | 10 | 5 | 1.00 | 5 | 0.89 (0.20-3.88) | 0.88 |
|  | Former | 2 | 0 | 1.00 | 2 | 1.90 (0.78-4.61) | 0.16 |
|  | Current | 1 | 0 | 1.00 | 1 | - | - |
| Urinary tract disease |  |  |  |  |  |  |  |
| Kidney failure | Never | 93 | 34 | 1.00 | 59 | 1.77 (1.02-3.07) | 0.04 |
|  | Former | 64 | 27 | 1.00 | 37 | 1.91 (1.04-3.50) | 0.04 |
|  | Current | 28 | 9 | 1.00 | 19 | 2.95 (1.12-7.77) | 0.03 |
| Abnormal clinical, lab findings | Never | 61 | 32 | 1.00 | 29 | 1.35 (0.74-2.45) | 0.33 |
|  | Former | 24 | 14 | 1.00 | 10 | 0.60 (0.26-1.42) | 0.25 |
|  | Current | 31 | 19 | 1.00 | 12 | 1.42 (0.66-3.06) | 0.37 |
| Transport injuries |  |  |  |  |  |  |  |
| Motor vehicle accidents | Never | 82 | 62 | 1.00 | 20 | 1.23 (0.67-2.23) | 0.50 |
|  | Former | 36 | 19 | 1.00 | 17 | 2.29 (1.09-4.78) | 0.03 |
|  | Current | 55 | 45 | 1.00 | 10 | 1.58 (0.69-3.59) | 0.28 |
| Unintentional injuries |  |  |  |  |  |  |  |
| Falls | Never | 38 | 22 | 1.00 | 16 | 1.04 (0.34-3.14) | 0.95 |
|  | Former | 25 | 13 | 1.00 | 12 | 1.17 (0.51-2.64) | 0.71 |
|  | Current | 18 | 16 | 1.00 | 2 | 0.56 (0.11-2.71) | 0.47 |
| Other non-transport accidents combined | Never | 71 | 48 | 1.00 | 23 | 0.80 (0.45-1.40) | 0.42 |
|  | Former | 33 | 21 | 1.00 | 12 | 1.41 (0.62-3.21) | 0.41 |
|  | Current | 82 | 61 | 1.00 | 21 | 1.60 (0.85-3.00) | 0.15 |
| Self-harm, interpersonal violence |  |  |  |  |  |  |  |
| Suicide | Never | 47 | 40 | 1.00 | 7 | 0.98 (0.26-3.64) | 0.98 |
|  | Former | 34 | 22 | 1.00 | 12 | 0.85 (0.41-1.74) | 0.65 |
|  | Current | 69 | 52 | 1.00 | 17 | 1.24 (0.65-2.34) | 0.51 |
| Homicide | Never | 29 | 22 | 1.00 | 7 | 0.97 (0.35-2.65) | 0.95 |
|  | Former | 6 | 4 | 1.00 | 2 | 0.31 (0.05-1.88) | 0.20 |
|  | Current | 20 | 19 | 1.00 | 1 | 0.27 (0.03-2.23) | 0.22 |
| All other diseases (residual) | Never | 443 | 208 | 1.00 | 235 | 1.29 (1.01-1.65) | 0.04 |
|  | Former | 263 | 123 | 1.00 | 140 | 1.25 (0.94-1.67) | 0.13 |
|  | Current | 195 | 125 | 1.00 | 70 | 1.48 (0.99-2.20) | 0.06 |
| All other causes/all unknown causes | Never | 641 | 483 | 1.00 | 158 | 0.95 (0.77-1.18) | 0.65 |
|  | Former | 237 | 144 | 1.00 | 93 | 1.08 (0.79-1.48) | 0.62 |
|  | Current | 240 | 187 | 1.00 | 53 | 1.06 (0.71-1.58) | 0.69 |

Multivariable adjustment for age, sex, education, race, income, alcohol, smoking status, BMI, physical activity, and survey year
